# Synthesis, quantitative structure–property relationship study of novel fluorescence active 2-pyrazolines and application

**DOI:** 10.1098/rsos.171964

**Published:** 2018-03-14

**Authors:** Adel S. Girgis, Altaf H. Basta, Houssni El-Saied, Mohamed A. Mohamed, Ahmad H. Bedair, Ahmad S. Salim

**Affiliations:** 1 Pesticide Chemistry Department, National Research Centre, Dokki, Giza 12622, Egypt; 2 Cellulose and Paper Department, National Research Centre, Dokki, Giza 12622, Egypt; 3 Forgery Research Department, Medico-legal Sector, Ministry of Justice, Cairo, Egypt; 4 Chemistry Department, Faculty of Science, Al-Azhar University, Cairo, Egypt

**Keywords:** pyrazoline derivatives, fluorescence compounds, fluorescence quantum yield, quantitative structure–property relationship studies, security marker for documents

## Abstract

A variety of fluorescence-active fluorinated pyrazolines **13–33** was synthesized in good yields through cyclocondensation reaction of propenones **1–9** with aryl hydrazines **10–12**. Some of the synthesized compounds provided promising fluorescence properties with quantum yield (*Φ*) higher than that of quinine sulfate (standard reference). Quantitative structure–property relationship studies were undertaken supporting the exhibited fluorescence properties and estimating the parameters governing properties. Five synthesized fluorescence-active pyrazolines (**13**, **15**, **18**, **19** and **23**) with variable *Φ* were selected for treating two types of paper sheets (Fabriano and Bible paper). These investigated fluorescence compounds, especially compounds **19** and **23**, provide improvements in strength properties of paper sheets. Based on the observed performance they can be used as markers in security documents.

## 1. Introduction

Fluorescence-active compounds are important materials due to their potential application in various fields, such as medicine, dyes, fluorescent labelling, biological detectors and cosmic-ray detection. Moreover, they are most commonly used in fluorescent lamps, quantum dots and as security markers for safety documents [1–3]. They can be divided into two categories: organic and inorganic-based fluorescence compounds [3–8]. The inorganic-based fluorescence compounds are zinc sulfide, cadmium sulfide, silica nanoparticles, carbon dots, as well as upconversion nanoparticles, gold or silver nanoparticles, etc. [8]. Unfortunately, some of these inorganic compounds, e.g. zinc sulfide and cadmium sulfide, have number of disadvantages that restrict their use. These compounds have toxicity and low quantum efficiency.

Organic fluorescent compounds are considered superior to inorganic ones due to their broad range of emission wavelengths and high luminous efficiency. 1,3,5-Triaryl-2-pyrazolines are important fluorescence-active heterocycles characterized by blue fluorescence with high quantum yield [9]. These properties make them accessible for many applications including photoconductive and emitting materials [10], as well as brightening agents for synthetic fibres, plastics and paper [10–12].

Paper is a major product of lignocellulosic materials (wood and non-wood). Functional or special papers are used for specific purposes, such as waterproof paper, carbon paper, cast-coated barrier paper, durable documents, decorative papers, electrical and magnetic paper, etc. Much literature is concerned with the role of cellulosic fibres, sizing agent, metal complexes, fire retardant additives, coating by biopolymers, surrounding environment in the quality and durability of paper. For forgery purposes, safety paper must be durable, and include safety marker resistance to forgery and counterfeiting [13–20].

In our previous work on the subject of fluorescence heterocyclic compounds for safety paper purposes, we reported the synthesis of 2-alkoxy-3-pyridinecarbonitrile derivatives, as well as heterocyclic compounds gathering the whole functional moieties responsible for fluorescence properties, via a variety of pyridine derivatives possessing both amino and alkoxy groups oriented o- and o′-positions of the pyridine nucleus and neighbouring to nitrile functions [3]. The behaviour of these compounds in nanoparticle form as security markers for production of unfalsifiable documents by erasure technique (chemical and mechanical) was also studied [20].

This study is directed towards synthesis of novel 1,3,5-triaryl-2-pyrazolines with fluorine substituent. Interest in these analogues is attributed to the unique properties of fluorine-containing compounds, such as thermal stability and lipophilicity [21]. Quantitative structure–property relationship (2D-QSPR) studies [22] are also undertaken in this work, to validate the fluorescence behaviour of the synthesized pyrazolines and identify the parameters controlling properties (mentioned in the electronic supplementary material). Moreover, the beneficial role of these fluorescence compounds as surface treating agents to paper sheets was studied, via estimating the paper strength properties and fluorescence view of paper sheets under UV lamp. This study was carried out as a precursor for the forthcoming work on evaluating the paper resistance to erasure as safety value documents.

## 2. Experimental set-up

### 2.1. Synthesis of 4,5-dihydro-1*H*-pyrazoles (general procedure)

**Scheme 1.**
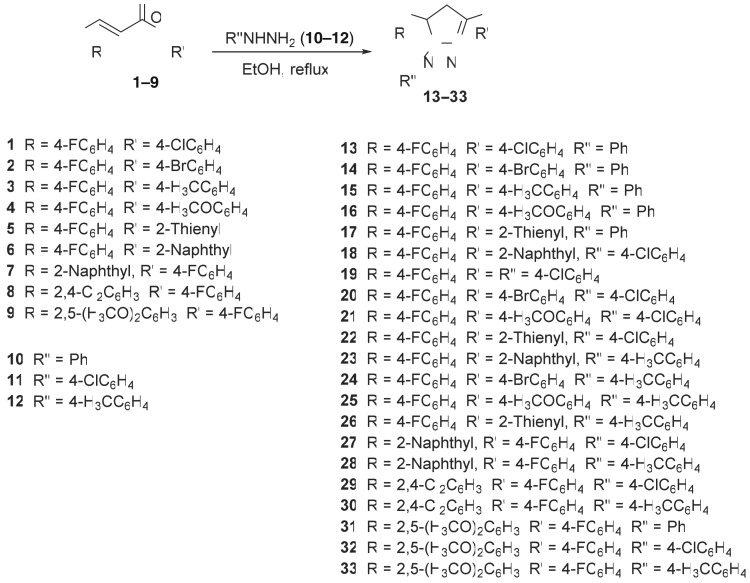
Synthetic route towards 2-pyrazolines **13**–**33**.

1,3,5-Triaryl-4,5-dihydro-1*H*-pyrazoles **13–33** were synthesized through cyclocondensation reaction of equimolar amounts of 1,3-diaryl-2-propen-1-ones **1–9** with aryl hydrazines **10–12** in refluxing ethanol. The solid that separated upon storing the reaction mixture at room temperature overnight was collected and crystallized from a suitable solvent affording the corresponding pyrazolines **13–33**. The obtained yields are in the range from 69 to 89%. The synthetic route towards 2-pyrazolines **13–33** is shown in scheme 1; while the methods of synthesizing these fluorescence compounds are detailed in the electronic supplementary material (Material and methods).

Melting points were recorded on a Stuart SMP3 melting point apparatus. IR spectra (KBr) were recorded on a Shimadzu FT-IR 8400S spectrophotometer. ^1^H-NMR spectra were recorded on Varian Mercury 300 (300 MHz) and Bruker Ascend 400/R (400 MHz) spectrometers. ^13^C-NMR spectra were recorded on a Bruker Ascend 400/R (100 MHz) spectrometer. This analysis was only conducted for four promising compounds with greatest quantum yield, *Φ*_s_ (**13**, **15**, **18** and **23**). Compounds **1–9** [21,23–28] were prepared according to the reported procedures. UV spectra were recorded on a Shimadzu UV-1800 spectrophotometer with slit width of 1.0 nm. Emission spectra were determined on a LUMINA fluorescence spectrometer with excitation slit of 2.5 nm and emission slit of 2.5 nm.

### 2.2. Quantitative structure–property relationship studies

2D-QSPR studies were undertaken using comprehensive descriptors for structural and statistical analysis (CODESSA-Pro) software. Florescence active pyrazolines **13–33** were used as training set for constructing the 2D-QSPR model. Geometry of the training set compounds was initially optimized by AM1 technique [29,30], then exported to CODESSA-Pro for the final geometry optimization (MOPAC software). CODESSA-Pro calculated 821 molecular descriptors (constitutional, topological, geometrical, charge-related, semi-empirical, thermodynamical, molecular-type, atomic-type and bond-type descriptors) for the exported 21 training set fluorescence-active pyrazolines. Different mathematical transformations (including property (quantum yield), 1/property, log(property) and 1/log(property)) of the experimentally observed training set compounds were used for searching for the best QSPR model. The best multi-linear regression (BMLR) technique was used which is a stepwise search for the best *n*-parameter regression equations (where *n* stands for the number of descriptors used), based on the highest *R*^2^ (squared correlation coefficient), *R*^2^cvOO (squared cross-validation ‘leave one-out, LOO’ coefficient), *R*^2^cvMO (squared cross-validation ‘leave many-out, LMO’ coefficient), *F* (Fisher statistical significance criteria) values and *s*^2^ (standard deviation). The QSPR models up to 5-descriptor model describing the properties of the fluorescence-active agents were generated (obeying the thumb rule of 5 : 1, which is the ratio between the data points and the number of QSPR descriptors).

Another validation technique was also considered to examine the robustness of the 2D-QSPR model, where part of the available data (two-thirds of the data) was used for determining a QSPR model and the remaining data points (one-third of the data) was used as a test set (external validation) [31].

(i) All the available data points (21 fluorescence-active pyrazolines **13–33**) were arranged in the descending order of *Φ*_s_ values and separated into three subsets (A, B and C) by selection of every third point from the original dataset in order to obtain fair distribution of the investigated property values for each subset.
(ii) Three new datasets were constructed using the three binary sums combinations: A + B, A + C and B + C. Then, BMLR-QSPR modelling procedure was applied to the three datasets obtained.
(iii) The complementary parts to each of the three datasets (C, B and A, respectively) were used as external validation datasets by considering their consistency.

### 2.3. Pyrazoline derivative-treated paper sheets and tests

In this study, two different types of paper sheets were used, to examine the behaviour of the synthesized fluorescence heterocycles on paper sheets. The first type was Fabriano® paper (80 g m^−2^), produced according to the ISO 9706 regulation. The paper sheets are wood free, with a neutral-alkaline sizing and an alkaline buffer content consisting of at least 2.5% of carbonate calcium to neutralize the acidic action of the environment. The cellulose used for Fabriano paper is bleached without chlorine, no dioxin is generated. The second paper type was Bible paper (45 g m^−2^), made from 25% cotton and linen in combination with chemical wood pulp. Both types of papers had long life and more durable compared to wood pulp papers. The purchased papers were surface treated with representative fluorescence-active compounds **13**, **15**, **18**, **19** and **23** dissolved in CHCl_3_ in ratio 0.1% (w/v). To avoid crumbling of paper sheets due to treatment, they were subjected to placement between heavy loading materials. The dried paper sheets were subjected to conditioning before testing, at relative humidity 50–55% and temperature 21 ± 2°C, for 24 h.

Strength properties, such as tensile index, burst index and tear index, were tested for untreated and fluorescence-treated paper sheets. For each test, at least five measurements were carried out.

The fluorescence behaviour of paper sheets was measured via a Thermo Scientific Lumina spectrometer and VSC®6000. The inspecting of active ultraviolet area was via a video spectral comparator (VSC®6000).

## 3. Results and discussion

### 3.1. Synthesis of 4,5-dihydro-1*H*-pyrazoles

Cyclocondensation reaction of 1,3-diaryl-2-propen-1-ones **1–9** with aryl hydrazines **10–12** in refluxing ethanol gave the corresponding 1,3,5-triaryl-4,5-dihydro-1*H*-pyrazoles **13–33** in good yields (69–89%) (scheme 1). Spectroscopic (IR, ^1^H-NMR, ^13^C-NMR; electronic supplementary material, figures S1–S46) and elemental analysis data support the structures of **13–33**. ^1^H-NMR spectrum of compound **13** (a representative of the synthesized family) shows the diastereotopic pyrazolinyl *H_2_C*-4 as double doublet signals at *δ*_H_ = 3.08, 3.81 and the methine *HC*-5 at *δ*_H_ = 5.28 (double doublet signal due to its mutual vicinal coupling with the diasteretopic protons of pyrazolinyl *H_2_C*-4). ^13^C-NMR of compound **13** exhibits the pyrazolinyl H_2_*C*-4 and H*C*-5 at *δ*_C_ = 43.4, 64.0, respectively.

### 3.2. UV–visible absorption and fluorescence spectra

#### 3.2.1. UV–visible spectra

The absorption spectra of the synthesized pyrazolines **13–33** were determined in chloroform with constant concentration (4 mg l^−1^). The data of maximum absorption wavelength (*λ*_max_) and molar extinction coefficient (*ε*_max_) are recorded in table 1. All synthesized pyrazolines **13–33** revealed two prominent peaks around 238 and 358 nm, attributed to the π–π* and n–π* transitions, respectively. The effect of introducing different electronic donating (EDG) and withdrawing groups (EWG) in R, R’ and R’ groups of 1,3,5- triaryl-4,5-dihydro-1*H*-pyrazoline had a remarkable effect on the differences in both the position and intensity of absorption peaks. The relatively higher absorption intensity is noticed for compound **29**. This is probably ascribed to the combination of 4-chlorophenyl and 2,4-dichlorophenyl at 1- and 5-positions of pyrazoline heterocycle. The three chlorine atoms (auxochromes) in addition to the presence fluorine atom at phenyl groups attached to the 1-, 3- and 5-positions of the pyrazoline may explain this observation. It is also noticed that the presence of 2-naphthyl group in compound **18** provided red shifted absorption peak relative to the other synthesized analogues.

**Table 1.** Absorption, excitation and emission spectral properties of the prepared compounds in chloroform and fluorescence measurements of paper sheets.

	absorption		fluorescent measurements of active pyrazolines		
fluorescence-active pyrazolines	*λ*_max_ (nm)	*ε*_max_ × 10^3^	excitation *λ*_max_ (nm)	emission *λ*_max_ (nm)	фs
**13**	241	117.002	267	455.8	0.819
	367	67.096	369.3^a^		
**14**	241	139.435	268	458.2	0.49
	283	81.428	300		
	368	78.562	370.8^a^		
**15**	242	45.431	265	443.7	0.858
	358	37.171	363.2^a^		
**16**	253	57.851	268	440.5	0.155
	357	49.450	362.3^a^		
**17**	241	105.106	275.4	469.8	0.455
	273	73.751			
	373	37.399	372.6^a^		
**18**	243	189.521	294	453.7	0.553
	277	116.559			
	375	83.686	373.7^a^		
**19**	241	189.938	276.7	455.4	0.802
	274	132.051			
	367	72.045	369.2^a^		
**20**	241	133.785	274.3	456.5	0.546
	370	80.249	370.6^a^		
**21**	238	141.010	272	438.7	0.269
	283	94.070	320		
	359	53.509	363^a^		
**22**	240	103.754	272	466.7	0.473
	261	93.941			
	374	59.594	374.3^a^		
**23**	242	104.820	300	471.6	0.844
	380	61.256	376.3^a^		
**24**	241	167.301	271.5	472.6	0.521
	270	111.432	325		
	373	54.232	373.9^a^		
**25**	240	163.369	268.5	459.4	0.267
	274	143.275	312		
	370	24.510	366.5^a^		
**26**	241	13.0535	271	479.4	0.333
	277	99.583			
	376	40.540	375.4^a^		
**27**	242	152.238	277	443.6	0.335
	287	74.065	318		
	361	71.158	365.2^a^		
**28**	241	159.988	316	464.1	0.21
	281	79.613			
	363	50.888	367^a^		
**29**	243	250.153	268	442.8	0.324
	280	187300.05			
	356	83.419	363.3^a^		
**30**	241	79.461	265	461.7	0.271
	360	50.911	364.3^a^		
**31**	240	133.444	308.9	444.3	0.297
	286	78.956			
	359	57.217	363.7^a^		
**32**	241	150.279	309	444.3	0.628
	281	96.454			
	361	56.393	365.1^a^		
**33**	242	191.911	306	469	0.588
	283	137.442			
	363	51.443	366.1^a^		

^a^Wavelength used in the calculation of fluorescence quantum yield of compounds **13–33.**

#### 3.2.2. Fluorescence spectra

**Figure 1.**
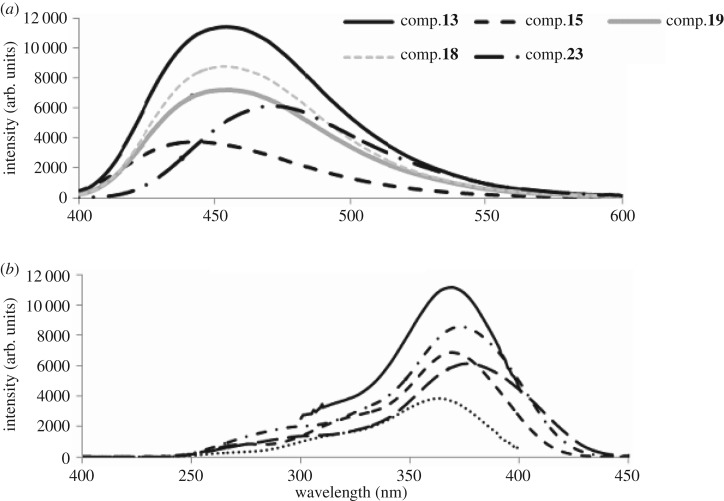
(*a*) Emission and (*b*) excitation spectra of representative fluorescent compounds with different quantum yield.

The maximum excitation wavelengths and the emission wavelengths for fluorescence spectra of the synthesized pyrazolines **13–33** were also measured in chloroform with constant concentration (1 × 10^−5^ mol l^−1^) (table 1). The spectra of the representative fluorescence compounds, with different quantum yields are illustrated in figure 1. From the observed data (table 1), it has been noticed that the synthesized pyrazolines are excited at 360–370 nm (corresponding to the high wavelength absorption band) affording fluorescence emission in the blue to green regions (emission peak wavelengths 438–471 nm). It has also been noticed that the quantum yield (*Φ*_sample_) value is greatly affected by the substitution type at 1-, 3- and 4-positions of the synthesized pyrazolines. The fluorescence quantum yield of the synthesized pyrazolines **13–33** was compared to that of quinine sulfate (used as reference compound, $\Phi_{\mathrm{ref}}$ = 0.546), and was calculated using the following equation (table 1):

3.1 $$\Phi_{\mathrm{sample}}=\Phi_{\mathrm{ref}}\left(\frac{F_{\mathrm{sample}}}{F_{\mathrm{ref}}}\right)\;\left(\frac{A_{\mathrm{ref}}}{A_{\mathrm{sample}}}\right)\;\left(\frac{{n^{2}}_{\mathrm{sample}}}{{n^{2}}_{\mathrm{ref}}}\right),$$

where *A* denotes absorbance, *F* denotes fluorescence, *F*_sample_, *A*_sample_, *n*_sample_ and *F*_ref_, *A*_ref_, *n*_ref_ are relative integrated fluorescence intensities, absorbance at excitation wavelength and refractive index of the sample and reference, respectively.

From the experimental data calculated and recorded in table 1, it is evident that the different substituents with EDG and EWG on both R’ and R'‘ groups, as well as their different positions at the 1,3-pyrazoline moiety had a great effect on both the intensity and the emission maxima wavelengths compared to the effect of the substituents on R groups. As can be seen, the presence of EWG and bulky groups in R’ such as present in compounds **13**, **14**, **18**, **19**, **20**, **27** and **32** led to higher emission intensity together with blue shifting of emission spectra than the other synthesized compounds. On the contrary, the presence of EDG on both R’ and R'‘ (e.g. compounds **25, 16, 28 and 33**) led to remarkable decreasing of the emission intensity without remarkable effect on the position of emission spectra.

From the foregoing data, it could be concluded that all the synthesized fluorescence-active fluorinated pyrazolines **13–33** exhibited vivid fluorescence properties with different quantum yields, ranging from 0.155 to 0.858. Five compounds with quantum yields of 0.858, 0.844, 0.819, 0.802, 0.553 (**13**, **15**, **18**, **19** and **23**, respectively) are candidates for further studies regarding the possibility of surface-applying these active fluorinated pyrazolines for production of functional paper sheets (strength and fluorescence performance).

### 3.3. Quantitative structure–property relationship

#### 3.3.1. Modelling

**Figure 2.**
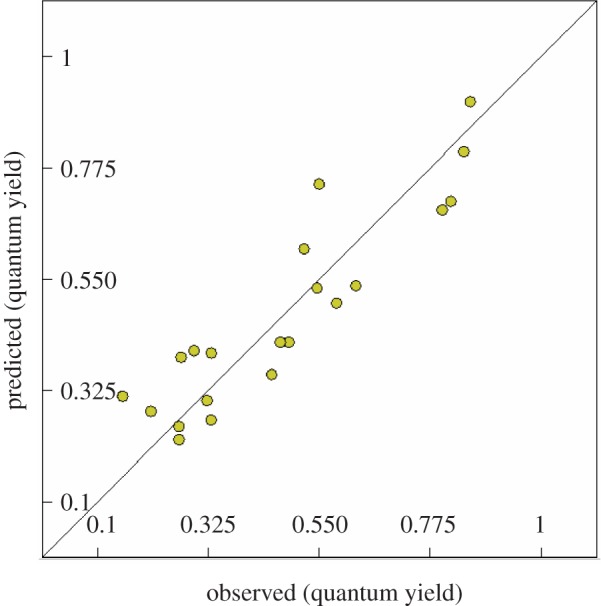
BMLR-QSPR model plot of correlations representing the observed versus predicted quantum yield (*Φ*_s_) values for the synthesized pyrazolines **13–33**.

Quantitative structure–property relationships have been a major part of many important scientific studies attracting attention of researchers not only for designing and developing agents of better behavioural manifestation but also to validate the experimental observed properties. This is due to the capability of QSPR to represent mathematical relationships between the property of interest and descriptors (physico-chemical parameters) based on the molecular structure. This study deals with QSPR study of fluorescence properties for the synthesized pyrazolines to explore the controlling parameters governing properties. CODESSA-Pro software was used for conducting the present QSPR study employing the 21 synthesized fluorescence-active fluorinated pyrazolines **13–33** which exhibit variable properties (quantum yields). The BMLR-QSPR model obtained for the present study is statistically significant (table 2 and figure 2) representing the observed versus predicted/estimated quantum yield values (observed 0.155–0.858, predicted 0.226–0.905). The observed and estimated quantum yield values of the fluorescence-active pyrazolines are exhibited in table 3.

**Table 2:** Descriptors of the BMLR-QSPR model for the fluorescence-active fluorinated pyrazolines (**13–33**)^a^.

entry	ID	coefficient	*s*	*t*	descriptor
1	0	41.8763	6.981	5.998	intercept
2	D_1_	1.74869	0.236	7.403	LUMO + 1 energy
3	D_2_	−0.342689	0.082	−4.199	number of Br atoms
4	D_3_	−9.96951	1.777	−5.609	max. coulombic interaction for bond H–C
5	D_4_	−165.672	23.466	−7.060	positively charged part of partial charged surface area (MOPAC PC)

^a^*N* = 21, *n* = 4, *R*^2^ = 0.817, *R*^2^cvOO = 0.708, *R*^2^cvMO = 0.730, *F* = 17.881, *s*^2^ = 0.011. Quantum yield (*Φ*_s_) = 41.8763 + (1.74869 × *D*_1_) *Φ*_s_ (0.342689 × *D*_2_) (*Φ*_s_ (9.96951 × *D*_3_) *Φ*_s_ (165.672 × *D*_4_).

**Table 3:** Observed and estimated/predicted quantum yield values of the fluorescence-active pyrazolines (**13–33**) according to the BMLR-QSPR model.

entry	compound	observed *Φ*_s_^a^	estimated *Φ*_s_	error^b^
1	**13**	0.819	0.704	0.115
2	**14**	0.490	0.420	0.070
3	**15**	0.858	0.905	−0.047
4	**16**	0.155	0.310	−0.155
5	**17**	0.455	0.355	0.100
6	**18**	0.553	0.742	−0.189
7	**19**	0.802	0.686	0.116
8	**20**	0.546	0.529	0.017
9	**21**	0.269	0.250	0.019
10	**22**	0.473	0.422	0.051
11	**23**	0.844	0.806	0.038
12	**24**	0.521	0.607	−0.086
13	**25**	0.267	0.226	0.041
14	**26**	0.333	0.398	−0.065
15	**27**	0.335	0.261	0.074
16	**28**	0.210	0.282	−0.072
17	**29**	0.324	0.303	0.021
18	**30**	0.271	0.389	−0.118
19	**31**	0.297	0.403	−0.106
20	**32**	0.628	0.535	0.093
21	**33**	0.588	0.501	0.087

^a^Measured in chloroform.

^b^Error is the difference between the observed and estimated quantum yield values.

#### 3.3.2. Molecular descriptors

The first descriptor controlling the BMLR-QSPR (*t* = 7.403) is LUMO + 1 energy, which is a semi-empirical descriptor. Lowest unoccupied molecular orbital (LUMO) energy is determined by

3.2 $$\varepsilon_{\mathrm{LUMO}}=\langle \;\phi_{\mathrm{LUMO}}|\hat{F}|\phi_{\mathrm{LUMO}}\rangle ,$$

where $\phi_{\mathrm{LUMO}}$ stands for lowest unoccupied molecular orbital and $\hat{F}$ for Fock operator.

Number of Br atoms (second descriptor controlling the QSPR model, *t* = −4.199) is a constitutional descriptor that characterizes the atomic constitution. Its inductive effect (–*I*) influences the aryl group electrophilicity attached at the pyrazoline heterocycle. Maximum coulombic interaction for bond H–C (*t* = −5.609) is bond-type descriptor. Coulombic force is determined by the following equation:

3.3 $$E_{\mathrm{coulombic}}=\frac{q_{i}\times q_{j}}{D\times r_{ij}},$$

where *q_i_* and *q_j_* represent the point charges on atoms *i* and *j*, respectively, with *r_ij_* being the distance between them. *D* denotes the dielectric constant of the medium.

Positively charged part of partial charged surface area (MOPAC PC) is a charge-related descriptor. The partial positively charged surface area (PPSA) is determined by

3.4 $$\text{PPSA}1=\underset{A}{\sum }S_{A}\;A\in \{\delta_{A}>0),$$

where *S*_A_ stands for the positively charged solvent-accessible atomic surface area (electronic supplementary material, table S1, shows the descriptor value for each tested compound).

#### 3.3.3. Validation

Good statistical parameters were exhibited by the internal validation of the attained QSPP including squared cross-validation of LOO and LMO (*R*^2^cvOO, *R*^2^cvMO = 0.708, 0.730, respectively), which are comparable with the squared cross-validation of the QSPR model (*R*^2^ = 0.817). Standard deviation of the regression (*s*^2^ = 0.011) and Fisher criterion (*F* = 17.881) also support the robustness of the QSPR model.

The predicted quantum yield values shown by the high fluorescence-active pyrazolines **13**, **15**, **19** and **23** are correlated with the experimentally observed values (*Φ*_s(observed)_ = 0.858–0.802, *Φ*_s(estimated)_ = 0.905–0.686, error = −0.047–0.116). The same observations for the promising (**14**, **17**, **18**, **20**, **22**, **24**, **32** and **33**; *Φ*_s (observed)_ = 0.628–0.455, *Φ*_s (estimated)_ = 0.742–0.355, error = −0.189–0.100) and low fluorescence-active agents (**16**, **21**, **25**–**31**; *Φ*_s(observed)_ = 0.335–0.155, *Φ*_s (estimated)_ = 0.403–0.226, error = −0.118–0.074). These observations support that the BMLR-QSPR model is applicable for wide range of fluorescence-active fluorinated pyrazolines with high, mild and low properties.

Another validation technique was also considered to examine the robustness of the 2D-QSPR model, where part of the available data (two-thirds of the data) were used for determining a QSPR model and the remaining data points (one-third of the data) were used as a test set (external validation). The observed 3 descriptor BMLR-QSPR models due to this technique (*N* = 14) are statistically significant (*R*^2^ = 0.820, 0.786, 0.910; *R*^2^cv = 0.703, 0.631, 0.830; *R*^2^cvMO = 0.726, 0.659, 0.845; *F *= 15.221, 12.210, 33.770; *s*^2^ = 4.908, 0.014, 0.005 for the subset groups A + B, A + C and B + C, respectively) (electronic supplementary material, tables S1–S10, figures S48–S50). The predicted properties (quantum yield) for most of the agents are close to the experimentally observed values.

### 3.4. Evaluation of fluorescent pyrazoline derivative-treated paper sheets

**Figure 3.**
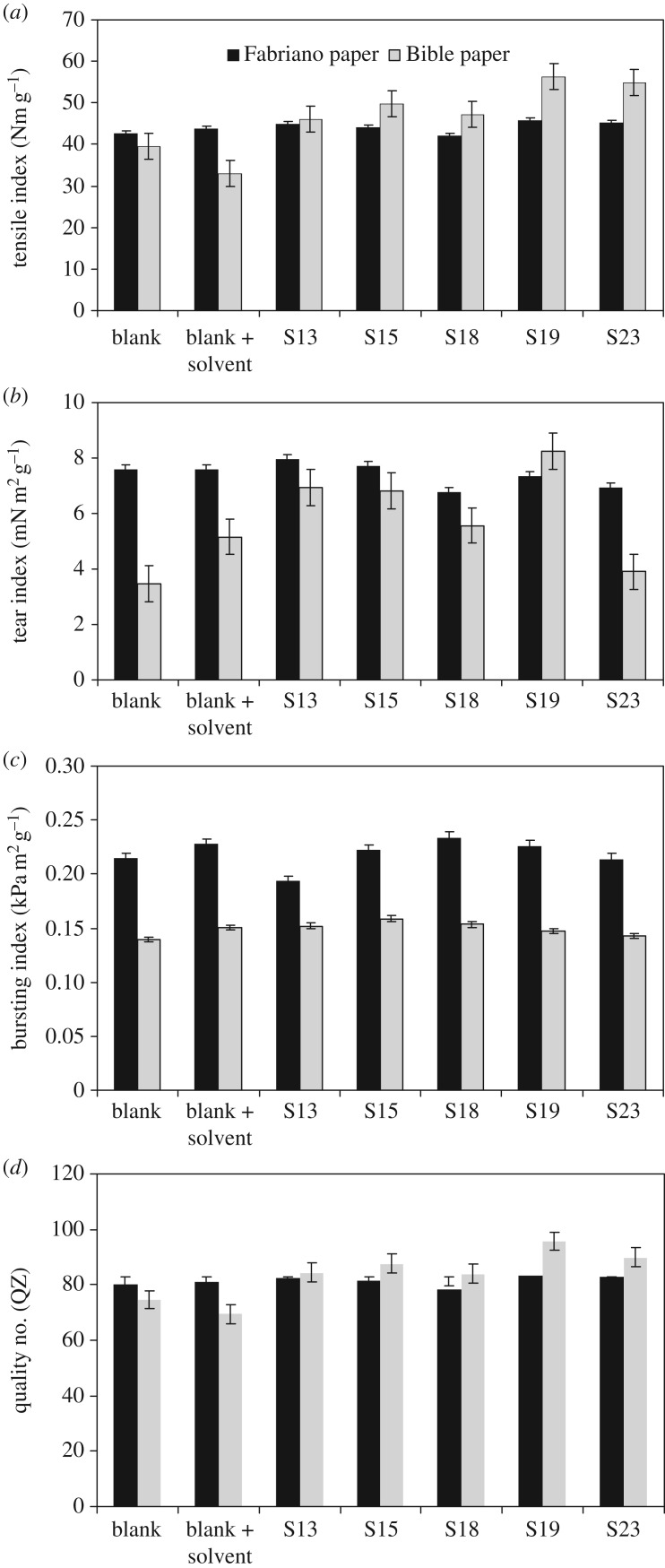
(*a*, *b*) Strength properties of paper sheets treated with representative fluorescent compounds. S13, S15, S18, S19 and S23 for Fabriano (SF) and Bible (SB) treated by compounds **13, 15, 18, 19** and **23**. (*c*, *d*) Strength properties of paper sheets treated with representative fluorescent compounds. S13, S15, S18, S19 and S23 for Fabriano (SF) and Bible (SB) treated by compounds **13, 15, 18, 19** and **23**.

The fluorescence spectra and strength properties of paper sheets treated with five representative fluorescence-active fluorinated pyrazolines **13**, **15**, **18**, **19** and **23** are given in figure 3 and table 4. These treated paper sheets were denoted with SF13, SF15, SF18, SF19 and SF23 for Fabriano papers; while for Bible paper were denoted as SB13, SB15, SB18, SB19, SB23, respectively. Table 4 shows that the treated paper sheets are excited at the range of 279–380 nm that corresponds to the absorption band in the solutions. It can be noticed that the decrease in the intensity of emission spectra is in the order of SF4 > SF5 > SF3 > SF1 > SF2 for the Fabriano papers; while for Bible sheets this order becomes SB3 > SB4 > SB1 > SB2 > SB5.

**Table 4.** Fluorescence spectra of treated paper sheets.

sample no.	excitation maxima *λ*_max_(nm)	emission *λ*_max_(nm)	fluorescence intensity × 10^4^
untreated SF^a^	—	—	—
SF1	282.8, 375	463.3	7651.56
SF2	290, 372.7	448.9	3528.30
SF3	280.1, 373.5	450.2	14730.14
SF4	279.4, 376.5	453.5	33676.10
SF5	283.5, 310.7, 379.9	471.1	17793.61
untreated SB^b^	—	—	—
SB1	279.5, 399.9	467.7	3077.90
SB2	279.7, 380	453.2	1688.62
SB3	279.9, 385.2	445.0	7896.15
SB4	281, 371	458.3	4755.48
SB5	280, 376	467.6	1360.27

^a^SF denotes the sample of the Fabriano paper.

^b^SB denotes the sample of the Bible paper.

On comparison with the emission bands of fluorescent compound solutions, it is noticed that the presence of the 4-chlorophenyl group at pyrazoline moiety for SF1, SF3, SB1 and SB3 leads to a red shift of the emission spectra; however, a blue shift of emission band is clear in the case of paper treated with compounds including 4-methylphenyl, as the case of SF2 and SB3. For SF4, SF5, SB4 and SB5, which were treated with fluorescent compounds including 2-naphthyl, no marked change in emission band is observed, in comparison to the data obtained in solution.

Table 4 also shows that the trend of emission intensities data for the treated Bible paper sheets is different from that for the Fabriano sheets, which may be ascribed to the change in cellulose substrates, basis weight and degree of sizing of the two types of paper sheets.

With regard to strength properties of treated paper sheets, figure 3*a,b* shows that all investigated compounds provide the improvement in quality numbers (*Q*_z_, indication to all strength properties). The improvement in the case of Bible paper sheets is greater than in the case of Fabriano paper sheets, especially for samples S19 and S23. The explanation of these observed data is probably ascribed to the basis weight of paper sheets. Bible paper is lower in grammage (45 g m^−2^), which promotes the diffusion of fluorescent solutions through the fibres and interact with the free hydroxyl groups of paper substrates, i.e. it leads to reformation of bonds but with higher strength hydrogen bonds. This view is emphasized from decreasing the strength of Bible paper sheets treated by chloroform solution only. The treating of Bible paper by chloroform leads to decrease in the energy of hydrogen bonds between fibres. It means that more free OH groups in cellulose substrate are possibly created.

**Figure 4.**
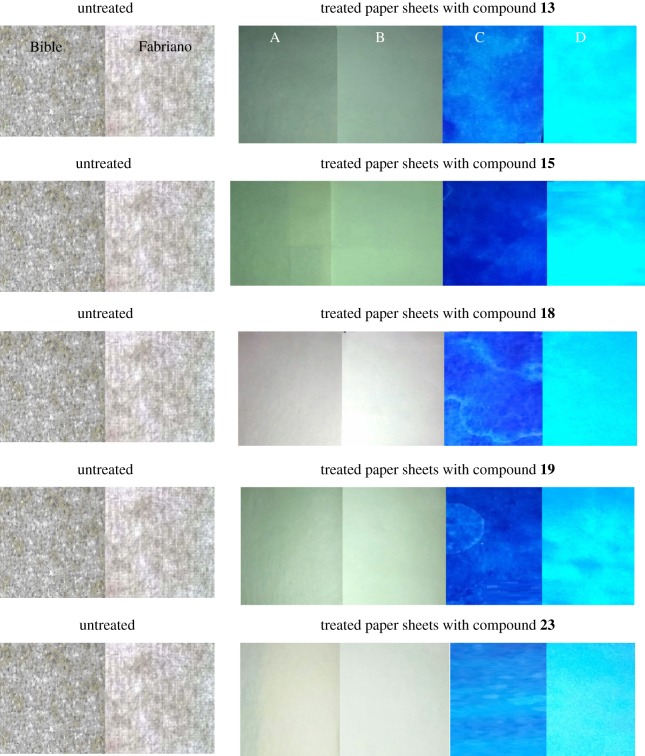
Photos of untreated and treated paper sheets. A and B indicate fluorescent compound-treated Bible and Fabriano sheets viewed with the naked eye; while C and D are UV images of these paper sheets.

The active ultraviolet area for the treated paper sheets was screened via a video spectral comparator (VSC®6000) as shown in figure 4. This figure shows that the treated paper sheets, denoted as SF13, SB13, SF18, SB18, SF19, SB19, SF23 and SB23, exhibit a green fluorescence light; while those denoted as SF15 and SB15 exhibit a blue fluorescence light. The Fabriano paper sheets show a greater intensity than the treated Bible paper sheets. This trend is related to the foregoing reason of lower grammage Bible paper sheets than Fabriano paper. The higher grammage paper sheets of Fabriano type (approx. 80 g m^−2^) may lead to a concentration of the fluorescent compounds on the surface of paper. As can be seen, the florescence intensity followed the order of quantum yields of compound solutions.

## 4. Conclusion

Synthesis of novel pyrazolines with fluorescence properties and application to paper sheets is the main objective of this study. 2D-QSPR study was undertaken for validating the fluorescence behaviour of the synthesized heterocycles. Some of the synthesized heterocycles (**13, 15, 19** and **23**) possessed promising fluorescence properties with quantum yield value reaching 0.86. The quantum yield values were estimated relative to the standard reference used (quinine sulfate). The greatest improvement in strength properties was observed in Bible paper sheets upon treatment with the fluorescence-active compounds **19** and **23**; while the greatest improvement in the fluorescence intensities was observed in treated Fabriano paper sheets. Both promising fluorescence and strength properties of treated paper sheets persuade us to examine, in forthcoming work, their safety behaviour towards resistance to counterfeiting and forgery, as well as ageing.

## Supplementary Material

Supplementary Materials

## Data accessibility

The datasets supporting this article have been uploaded as part of the electronic supplementary material. It includes graphic abstract, methods of synthesizing fluorescent pyrazolines in detail, together with charts and tables of analyses, BMLR-QSPR model plots and tables.
