# Supplementary material for: Synthesis, quantitative structure–property relationship study of novel fluorescence active 2-pyrazolines and application
Source: R Soc Open Sci. 2018 Mar 14;5(3):171964. doi: 10.1098/rsos.171964 (PMC5882720; doi:10.1098/rsos.171964)
Supplement: Supplementary Materials [file rsos171964supp1.docx]

**Supplementary materials**

(http://datadryad.org/review?doi=doi:10.5061/dryad.v150k)

**Article title: Synthesis, Quantitative Structure-Property Relationship (QSPR) Study of Novel Fluorescence Active 2-Pyrazolines and Application.**

**Authors and address:** Adel S. Girgis^a^ Altaf H. Basta^b^*, Houssni El-Saied,^b^ Mohamed A. Mohamed,^c^ Ahmad H. Bedair^c^, and Ahmad S. Salim^b^,

^a^Pesticide Chemistry Department, National Research Centre, Dokki, Giza 12622, Egypt

^b^Cellulose & Paper Dept., National Research Centre, Dokki, Giza 12622, Egypt

^c^Forgery Research Department, Medico-legal Sector, Ministry of Justice, Cairo, Egypt

^d^Chemistry Department, Faculty of Science, Al-Azhar University, Cairo, Egypt

**Journal: Royal Society Open Science**

----------------------------------------------------------------------------------

* Corresponding author; Emails: Altaf_Halim@yahoo.com & [Altaf_Basta2004@yahoo.com](mailto:Altaf_Basta2004@yahoo.com), Fax # 202-376180766 , Tel. & Fax. 202-33371718

**Graphic abstract**

**
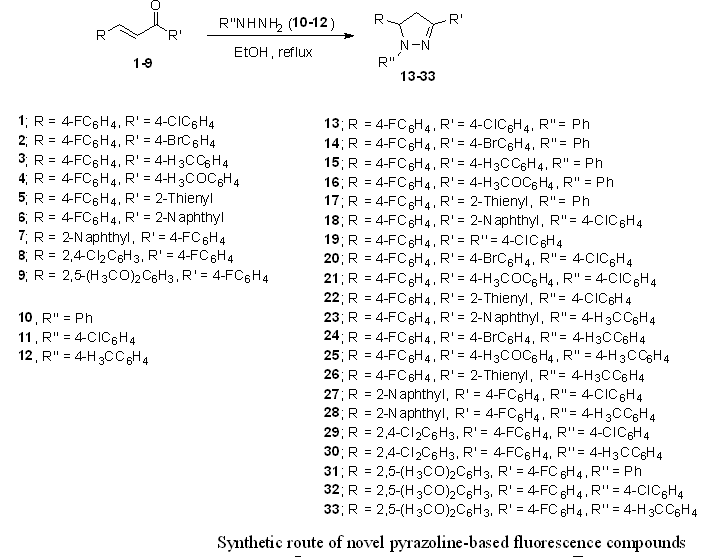
**


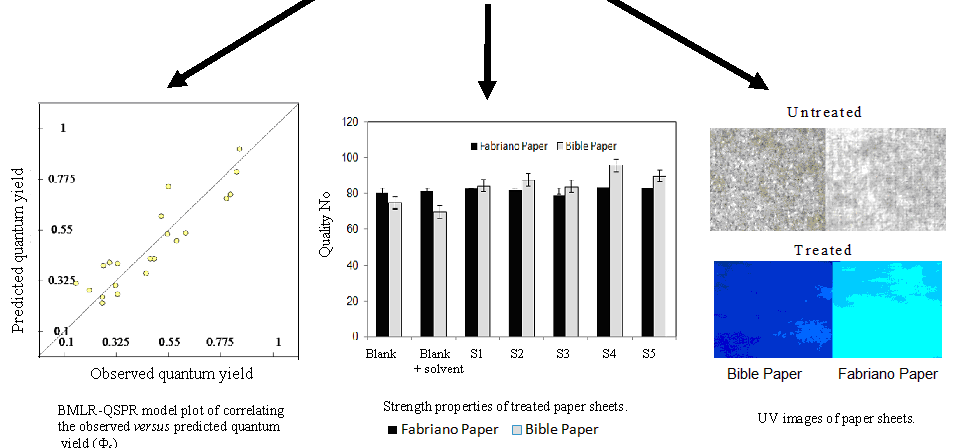


**Materials and Methods**

**2.1. Methods of synthesizing fluorescence pyrazolines 13-33.**

***2.1.1. 3-(4-Chlorophenyl)-5-(4-fluorophenyl)-1-phenyl-4,5-dihydro-1H-pyrazole (13)***

This compound is obtained from reaction of **1** and **10**, at reaction time 6 h, It almost colorless microcrystals from n-butanol, mp 156–158 °C, yield 89%. IR *ν* (cm^-1^): 1589, 1558, 1504, 1493. ^1^H-NMR δ (ppm): (300 MHz) 3.08 (dd, *J* = 7.5, 17.1 Hz, 1H, upfield H of pyrazolinyl *H_2_C*-4), 3.81 (dd, *J* = 12.5, 17.0 Hz, 1H, downfield H of pyrazolinyl *H_2_C*-4), 5.28 (dd, *J* = 7.2, 12.3 Hz, 1H, pyrazolinyl *HC*-5), 6.78-6.85 (m, 1H, arom. H), 7.01-7.43 (m, 10H, arom. H), 7.65 (d, *J* = 8.7 Hz, 2H, arom. H). ^13^C-NMR *δ* (ppm): (100 MHz) 43.4 (pyrazolinyl H_2_*C*-4), 64.0 (pyrazolinyl H*C*-5), 113.5, 116.0, 116.2, 119.5, 125.3, 126.9, 127.1, 127.5, 127.6, 128.8, 128.9, 129.0, 129.1, 131.2, 134.4, 138.0, 138.1, 144.5, 145.6, 161.0, 163.4 (arom. *C*). Elemental analysis: C_21_H_16_ClFN_2_ required C, 71.90; H, 4.60; N, 7.99, found C, 72.09; H, 4.71; N, 8.07.

***2.1.2. 3-(4-Bromophenyl)-5-(4-fluorophenyl)-1-phenyl-4,5-dihydro-1H-pyrazole (14)***

This pyrazole derivative is obtained from reaction of **2** and **10**, at reaction time 7 h. It is pale yellow microcrystals from ethanol, mp 144–146 °C, yield 71% (1.40 g). IR *ν* (cm^-1^): 1597, 1570, 1543, 1499. ^1^H-NMR δ (ppm): (400 MHz) 3.09 (dd, *J* = 7.2, 17.1 Hz, 1H, upfield H of pyrazolinyl *H_2_C*-4), 3.81 (dd, *J* = 12.4, 17.0 Hz, 1H, downfield H of pyrazolinyl *H_2_C*-4), 5.28 (dd, *J* = 7.2, 12.4 Hz, 1H, pyrazolinyl *HC*-5), 6.85 (t, *J* = 7.3 Hz, 1H, arom. H), 7.03-7.09 (m, 4H, arom. H), 7.21-7.25 (m, 2H, arom. H), 7.28-7.32 (m, 2H, arom. H), 7.39 (br s, 2H, arom. H), 7.53 (d, *J* = 8.6 Hz, 2H, arom. H), 7.60 (dd, *J* = 1.9, 6.7 Hz, 2H, arom. H). Elemental analysis: C_21_H_16_BrFN_2_ required C, 63.81; H, 4.08; N, 7.09, found C, 64.06; H, 4.17; N, 7.31.

***2.1.3. 5-(4-Fluorophenyl)-3-(4-methylphenyl)-1-phenyl-4,5-dihydro-1H-pyrazole (15)***

It is obtained from reaction of **3** and **10**, at reaction time 9 h. It is pale yellow microcrystals from n-butanol, mp 147–149 °C, yield 82% (1.35 g). IR *ν* (cm^-1^): 1597, 1551, 1497. ^1^H-NMR δ (ppm): (300 MHz) 2.39 (s, 3H, *CH_3_*), 3.10 (dd, *J* = 7.4, 17.0 Hz, 1H, upfield H of pyrazolinyl *H_2_C*-4), 3.83 (dd, *J* = 12.5, 17.0 Hz, 1H, downfield H of pyrazolinyl *H_2_C*-4), 5.24 (dd, *J* = 7.2, 12.3 Hz, 1H, pyrazolinyl *HC*-5), 6.79-7.64 (m, 13H, arom. H). ^13^C-NMR *δ* (ppm): (100 MHz) 21.4 (*C*H_3_), 43.7 (pyrazolinyl H_2_*C*-4), 63.8 (pyrazolinyl H*C*-5), 113.4, 115.9, 116.1, 119.2, 125.8, 127.5, 127.6, 129.0, 129.3, 129.9, 138.4, 138.5, 138.8, 144.9, 145.0, 160.9, 163.4 (arom. *C*). Elemental analysis: C_22_H_19_FN_2_ required C, 79.97; H, 5.80; N, 8.48, found C, 80.04; H, 5.92; N, 8.64.

***2.1.4. 5-(4-Fluorophenyl)-3-(4-methoxyphenyl)-1-phenyl-4,5-dihydro-1H-pyrazole (16)***

It is obtained from reaction of **4** and **10**. Reaction time 7 h, and its microcrystals from methanol is colorless, with mp 133–135 °C, yield 75%. IR *ν* (cm^-1^): 1593, 1497, 1420. ^1^H-NMR δ (ppm): (400 MHz) 3.10 (dd, *J* = 7.2, 17.0 Hz, 1H, upfield H of pyrazolinyl *H_2_C*-4), 3.82 (dd, *J* = 12.2, 17.0 Hz, 1H, downfield H of pyrazolinyl *H_2_C*-4), 3.87 (s, 3H, O*CH_3_*), 5.23 (dd, *J* = 7.2, 12.2 Hz, 1H, pyrazolinyl *HC*-5), 6.80-7.70 (m, 13H, arom. H). Elemental analysis: C_22_H_19_FN_2_O required C, 76.28; H, 5.53; N, 8.09, found C, 76.17; H, 5.39; N, 7.89.

***2.1.5. 5-(4-Fluorophenyl)-1-phenyl-3-(2-thienyl)-4,5-dihydro-1H-pyrazole (17)***

It is obtained from reaction of **5** and **10**, at reaction time 7 h. It is pale yellow microcrystals from n-butanol, mp 137–139 °C, yield 75%. IR *ν* (cm^-1^): 1593, 1570, 1499. ^1^H-NMR δ (ppm): (400 MHz) 3.13 (dd, *J* = 7.2, 16.9 Hz, 1H, upfield H of pyrazolinyl *H_2_C*-4), 3.85 (dd, *J* = 12.3, 16.9 Hz, 1H, downfield H of pyrazolinyl *H_2_C*-4), 5.27 (dd, *J* = 7.2, 12.3 Hz, 1H, pyrazolinyl *HC*-5), 6.84 (t, *J* = 7.3 Hz, 1H, arom. H), 7.04-7.09 (m, 6H, arom. H), 7.20-7.24 (m, 2H, arom. H), 7.31-7.36 (m, 3H, arom. H). Elemental analysis: C_19_H_15_FN_2_S required C, 70.78; H, 4.69; N, 8.69, found C, 70.62; H, 4.62; N, 8.93.

***2.1.6. 1-(4-Chlorophenyl)-5-(4-fluorophenyl)-3-(2-naphthyl)-4,5-dihydro-1H-pyrazole (18)***

It is obtained from reaction of **6** and **11**, at reaction time 8 h. It is pale yellow microcrystals from n-butanol, mp 184–186 °C, yield 80% (1.60 g). IR *ν* (cm^-1^): 1595, 1495, 1439. ^1^H-NMR δ (ppm): (400 MHz) 3.25 (dd, *J* = 7.0, 17.0 Hz, 1H, upfield H of pyrazolinyl *H_2_C*-4), 3.93 (dd, *J* = 12.3, 17.0 Hz, 1H, downfield H of pyrazolinyl *H_2_C*-4), 5.26 (dd, *J* = 7.0, 12.2 Hz, 1H, pyrazolinyl *HC*-5), 7.03-7.08 (m, 4H, arom. H), 7.16-7.19 (m, 2H, arom. H), 7.28-7.32 (m, 2H, arom. H), 7.50-7.53 (m, 2H, arom. H), 7.81-7.88 (m, 4H, arom. H), 8.17 (dd, J = 1.6, 8.6 Hz, 1H, arom. H). ^13^C-NMR *δ* (ppm): (100 MHz) 43.6 (pyrazolinyl H_2_*C*-4), 63.9 (pyrazolinyl H*C*-5), 114.6, 116.1, 116.3, 123.4, 124.2, 125.4, 126.4, 126.58, 126.59, 127.5, 127.6, 127.9, 128.1, 128.3, 128.9, 129.2, 130.0, 133.3, 133.6, 137.7, 137.8, 143.1, 147.4, 161.0, 163.5 (arom. *C*). Elemental analysis: C_25_H_18_ClFN_2_ required C, 74.90; H, 4.53; N, 6.99, found C, 75.12; H, 4.71; N, 7.18.

***2.1.7. 1,3-Bis(4-chlorophenyl)-5-(4-fluorophenyl)-4,5-dihydro-1H-pyrazole (19)***

It is obtained from reaction of **1** and **11**, at reaction time 8 h. It is almost colorless microcrystals from n-butanol, mp 141–143 °C, yield 81%. IR *ν* (cm^-1^): 1597, 1489. ^1^H-NMR δ (ppm): (300 MHz) 3.10 (dd, *J* = 7.1, 17.0 Hz, 1H, upfield H of pyrazolinyl *H_2_C*-4), 3.82 (dd, *J* = 12.3, 17.1 Hz, 1H, downfield H of pyrazolinyl *H_2_C*-4), 5.25 (dd, *J* = 7.1, 12.2 Hz, 1H, pyrazolinyl *HC*-5), 6.95-7.65 (m, 12H, arom. H). Elemental analysis: C_21_H_15_Cl_2_FN_2_ required C, 65.47; H, 3.92; N, 7.27, found C, 65.56; H, 4.03; N, 7.09.

***2.1.8. 3-(4-Bromophenyl)-1-(4-chlorophenyl)-5-(4-fluorophenyl)-4,5-dihydro-1H-pyrazole (20)***

It is obtained from reaction of **2** and **11**, at reaction time 7 h. It is colorless microcrystals from n-butanol, mp 164–166 °C, yield 79%. IR *ν* (cm^-1^): 1593, 1508, 1489. ^1^H-NMR δ (ppm): (400 MHz) 3.11 (dd, *J* = 7.1, 17.1 Hz, 1H, upfield H of pyrazolinyl *H_2_C*-4), 3.83 (dd, *J* = 12.3, 17.1 Hz, 1H, downfield H of pyrazolinyl *H_2_C*-4), 5.26 (dd, *J* = 7.0, 12.3 Hz, 1H, pyrazolinyl *HC*-5), 6.97 (dd, *J* = 2.1, 6.9 Hz, 2H, arom. H), 7.03-7.07 (m, 2H, arom. H), 7.15 (dd, *J* = 2.1, 6.9 Hz, 2H, arom. H), 7.25-7.29 (m, 2H, arom. H), 7.53 (dd, *J* = 1.9, 6.8 Hz, 2H, arom. H), 7.59 (dd, *J* = 1.8, 6.8 Hz, 2H, arom. H). Elemental analysis: C_21_H_15_BrClFN_2_ required C, 58.70; H, 3.52; N, 6.52, found C, 58.60; H, 3.38; N, 6.71.

***2.1.9. 1-(4-Chlorophenyl)-5-(4-fluorophenyl)-3-(4-methoxyphenyl)-4,5-dihydro-1H-pyrazole (21)***

It is obtained from reaction of **4** and **11**, at reaction time 7 h. It is colorless microcrystals from ethanol, mp 151–153 °C, yield 84%. IR *ν* (cm^-1^): 1607, 1593, 1493. ^1^H-NMR δ (ppm): (400 MHz) 3.10 (dd, *J* = 7.1, 17.1 Hz, 1H, upfield H of pyrazolinyl *H_2_C*-4), 3.82 (dd, *J* = 12.2, 17.0 Hz, 1H, downfield H of pyrazolinyl *H_2_C*-4), 3.86 (s, 3H, O*CH_3_*), 5.19 (dd, *J* = 7.1, 12.1 Hz, 1H, pyrazolinyl *HC*-5), 6.93-7.16 (m, 7H, arom. H), 7.26-7.37 (m, 3H, arom. H), 7.68 (d, *J* = 8.8 Hz, 2H, arom. H). Elemental analysis: C_22_H_18_ClFN_2_O required C, 69.38; H, 4.76; N, 7.36, found C, 69.20; H, 4.70; N, 7.26.

***2.1.10. 1-(4-Chlorophenyl)-5-(4-fluorophenyl)-3-(2-thienyl)-4,5-dihydro-1H-pyrazole (22)***

It is obtained from reaction of **5** and **11**, at reaction time 7 h. It is pale yellow microcrystals from ethanol, mp 144–146 °C, yield 79%. IR *ν* (cm^-1^): 1597, 1494. ^1^H-NMR δ (ppm): (400 MHz) 3.14 (dd, *J* = 7.0, 17.0 Hz, 1H, upfield H of pyrazolinyl *H_2_C*-4), 3.87 (dd, *J* = 12.2, 16.9 Hz, 1H, downfield H of pyrazolinyl *H_2_C*-4), 5.24 (dd, *J* = 7.1, 12.2 Hz, 1H, pyrazolinyl *HC*-5), 6.94-6.97 (m, 2H, arom. H), 7.03-7.09 (m, 4H, arom. H), 7.11-7.15 (m, 2H, arom. H), 7.27-7.30 (m, 2H, arom. H), 7.36 (dd, *J* = 1.0, 5.0 Hz, 1H, arom. H). Elemental analysis: C_19_H_14_ClFN_2_S required C, 63.95; H, 3.95; N, 7.85, found C, 63.89; H, 3.83; N, 7.65.

***2.1.11.5-(4-Fluorophenyl)-1-(4-methylphenyl)-3-(2-naphthyl)-4,5-dihydro-1H-pyrazole (23)***

It is obtained from reaction of **6** and **12**, at reaction time 6 h. It is pale yellow microcrystals from n-butanol, mp 188–189 °C, yield 84%. IR *ν* (cm^-1^): 1602, 1558, 1516, 1504. ^1^H-NMR δ (ppm): (400 MHz) 2.28 (s, 3H, *CH_3_*), 3.25 (dd, *J* = 7.4, 16.9 Hz, 1H, upfield H of pyrazolinyl *H_2_C*-4), 3.91-3.99 (m, 1H, downfield H of pyrazolinyl *H_2_C*-4), 5.27-5.32 (m, 1H, pyrazolinyl *HC*-5), 7.04-7.08 (m, 6H, arom. H), 7.28-7.36 (m, 2H, arom. H), 7.48-7.51 (m, 2H, arom. H), 7.81-7.88 (m, 4H, arom. H), 8.19 (d, *J* = 8.7 Hz, 1H, arom. H). ^13^C-NMR *δ* (ppm): (100 MHz) 20.5 (*C*H_3_), 43.5 (pyrazolinyl H_2_*C*-4), 64.2 (pyrazolinyl H*C*-5), 113.6, 115.9, 116.1, 123.5, 125.0, 125.4, 126.3, 126.5, 127.6, 127.7, 127.8, 128.1, 128.2, 128.7, 129.5, 129.7, 130.4, 133.3, 133.4, 138.39, 138.42, 142.5, 146.4, 160.9, 163.4 (arom. *C*). Elemental analysis: C_26_H_21_FN_2_ required C, 82.08; H, 5.56; N, 7.36, found C, 82.24; H, 5.67; N, 7.22.

***2.1.12. 3-(4-Bromophenyl)-5-(4-fluorophenyl)-1-(4-methylphenyl)-4,5-dihydro-1H-pyrazole (24)***

It is obtained from reaction of **2** and **12**, at reaction time 7 h. It is pale yellow microcrystals from n-butanol, mp 174–176 °C, yield 87%. IR *ν* (cm^-1^): 1607, 1514, 1489. ^1^H-NMR δ (ppm): (400 MHz) 2.27 (s, 3H, *CH_3_*), 3.07 (dd, *J* = 7.6, 17.0 Hz, 1H, upfield H of pyrazolinyl *H_2_C*-4), 3.79 (dd, *J* = 12.4, 17.0 Hz, 1H, downfield H of pyrazolinyl *H_2_C*-4), 5.25 (dd, *J* = 7.6, 12.4 Hz, 1H, pyrazolinyl *HC*-5), 6.97-7.07 (m, 6H, arom. H), 7.18-7.32 (m, 2H, arom. H), 7.52 (dd, *J* = 1.9, 6.8 Hz, 2H, arom. H), 7.59 (d, *J* = 8.6 Hz, 2H, arom. H). Elemental analysis: C_22_H_18_BrFN_2_ required C, 64.56; H, 4.43; N, 6.84, found C, 64.65; H, 4.49; N, 6.71.

***2.1.13. 5-(4-Fluorophenyl)-3-(4-methoxyphenyl)-1-(4-methylphenyl)-4,5-dihydro-1H-pyrazole (25)***

It is obtained from reaction of **4** and **12**, at reaction time 7 h. It is almost colorless microcrystals from n-butanol, mp 157–159 °C, yield 78%. IR *ν* (cm^-1^): 1605, 1510, 1422. ^1^H-NMR δ (ppm): (400 MHz) 2.27 (s, 3H, *CH_3_*), 3.09 (dd, *J* = 7.7, 16.9 Hz, 1H, upfield H of pyrazolinyl *H_2_C*-4), 3.81 (dd, *J* = 12.2, 16.9 Hz, 1H, downfield H of pyrazolinyl *H_2_C*-4), 3.87 (s, 3H, O*CH_3_*), 5.19 (dd, *J* = 7.7, 12.1 Hz, 1H, pyrazolinyl *HC*-5), 6.93-7.07 (m, 8H, arom. H), 7.27-7.34 (m, 2H, arom. H), 7.69 (d, *J* = 8.8 Hz, 2H, arom. H). Elemental analysis: C_23_H_21_FN_2_O required C, 76.64; H, 5.87; N, 7.77, found C, 76.78; H, 5.96; N, 7.66.

***2.1.14. 5-(4-Fluorophenyl)-1-(4-methylphenyl)-3-(2-thienyl)-4,5-dihydro-1H-pyrazole (26)***

It is obtained from reaction of **5** and **12**, at reaction time 8 h. It is pale yellow microcrystals from n-butanol, mp 148–150 °C, yield 74% (1.25 g). IR *ν* (cm^-1^): 1605, 1562, 1506. ^1^H-NMR δ (ppm): (400 MHz) 2.27 (s, 3H, *CH_3_*), 3.11 (dd, *J* = 7.6, 16.8 Hz, 1H, upfield H of pyrazolinyl *H_2_C*-4), 3.83 (dd, *J* = 12.2, 16.8 Hz, 1H, downfield H of pyrazolinyl *H_2_C*-4), 5.23 (dd, *J* = 7.6, 12.2 Hz, 1H, pyrazolinyl *HC*-5), 6.95 (d, *J* = 8.6 Hz, 2H, arom. H), 7.01-7.07 (m, 6H, arom. H), 7.30-7.34 (m, 3H, arom. H). Elemental analysis: C_20_H_17_FN_2_S required C, 71.40; H, 5.09; N, 8.33, found C, 71.59; H, 5.27; N, 8.37.

***2.1.15. 1-(4-Chlorophenyl)-3-(4-fluorophenyl)-5-(2-naphthyl)-4,5-dihydro-1H-pyrazole (27)***

It is obtained from reaction of **7** and **11,** at reaction time 7 h. It is colorless microcrystals from n-butanol, mp 146–147 °C, yield 73%. IR *ν* (cm^-1^): 1601, 1593, 1491. ^1^H-NMR δ (ppm): (400 MHz) 3.22 (dd, *J* = 7.4, 17.2 Hz, 1H, upfield H of pyrazolinyl *H_2_C*-4), 3.92 (dd, *J* = 12.3, 17.2 Hz, 1H, downfield H of pyrazolinyl *H_2_C*-4), 5.42 (dd, *J* = 7.4, 12.3 Hz, 1H, pyrazolinyl *HC*-5), 7.03-7.19 (m, 5H, arom. H), 7.28-7.57 (m, 4H, arom. H), 7.73 (dd, *J* = 5.4, 8.8 Hz, 2H, arom. H), 7.78-7.96 (m, 4H, arom. H). Elemental analysis: C_25_H_18_ClFN_2_ required C, 74.90; H, 4.53; N, 6.99, found C, 74.96; H, 4.62; N, 6.87.

***2.1.16. 3-(4-Fluorophenyl)-1-(4-methylphenyl)-5-(naphthyl)-4,5-dihydro-1H-pyrazole (28)***

It is obtained from reaction of **7** and **12**, at reaction time 7 h. It is colorless microcrystals from n-butanol, mp 140–142 °C, yield 74%. IR *ν* (cm^-1^): 1603, 1562, 1508. ^1^H-NMR δ (ppm): (400 MHz) 2.25 (s, 3H, *CH_3_*), 3.19 (dd, *J* = 7.9, 17.0 Hz, 1H, upfield H of pyrazolinyl *H_2_C*-4), 3.88 (dd, *J* = 12.4, 17.0 Hz, 1H, downfield H of pyrazolinyl *H_2_C*-4), 5.41 (dd, *J* = 7.9, 12.3 Hz, 1H, pyrazolinyl *HC*-5), 6.99-7.18 (m, 6H, arom. H), 7.46-7.53 (m, 3H, arom. H), 7.72-7.89 (m, 6H, arom. H). Elemental analysis: C_26_H_21_FN_2_ required C, 82.08; H, 5.56; N, 7.36, found C, 82.20; H, 5.69; N, 7.42.

***2.1.17. 1-(4-Chlorophenyl)-5-(2,4-dichlorophenyl)-3-(4-fluorophenyl)-4,5-dihydro-1H-pyrazole (29)***

It is obtained from reaction of **8** and **11**, at reaction time 7 h. It is colorless microcrystals from n-butanol, mp 175–177 °C, yield 76%. IR *ν* (cm^-1^): 1601, 1593, 1562, 1512. ^1^H-NMR δ (ppm): (400 MHz) 3.03 (dd, *J* = 6.7, 17.2 Hz, 1H, upfield H of pyrazolinyl *H_2_C*-4), 3.96 (dd, *J* = 12.3, 17.2 Hz, 1H, downfield H of pyrazolinyl *H_2_C*-4), 5.57 (dd, *J* = 6.7, 12.3 Hz, 1H, pyrazolinyl *HC*-5), 6.91 (d, *J* = 8.9 Hz, 2H, arom. H), 7.08-7.19 (m, 6H, arom. H), 7.50 (d, *J* = 1.8 Hz, 1H, arom. H), 7.70-7.73 (m, 2H, arom. H). Elemental analysis: C_21_H_14_Cl_3_FN_2_ required C, 60.10; H, 3.36; N, 6.67, found C, 60.27; H, 3.42; N, 6.86.

***2.1.18. 5-(2,4-Dichlorophenyl)-3-(4-fluorophenyl)-1-(4-methylphenyl)-4,5-dihydro-1H-pyrazole (30)***

It is obtained from reaction of **8** and **12**, at reaction time 7 h. It is pale yellow microcrystals from n-butanol, mp 144–146 °C, yield 73%. IR *ν* (cm^-1^): 1617, 1600, 1558, 1508. ^1^H-NMR δ (ppm): (300 MHz) 2.27 (s, 3H, *CH_3_*), 2.99 (dd, *J* = 6.9, 17.1 Hz, 1H, upfield H of pyrazolinyl *H_2_C*-4), 3.93 (dd, *J* = 12.3, 17.1 Hz, 1H, downfield H of pyrazolinyl *H_2_C*-4), 5.56 (dd, *J* = 7.2, 12.3 Hz, 1H, pyrazolinyl *HC*-5), 6.88-7.73 (m, 11H, arom. H). Elemental analysis: C_22_H_17_Cl_2_FN_2_ required C, 66.18; H, 4.29; N, 7.02, found C, 66.06; H, 4.15; N, 7.22.

***2.1.19. 5-(2,5-Dimethoxyphenyl)-3-(4-fluorophenyl)-1-phenyl-4,5-dihydro-1H-pyrazole (31)***

It is obtained from reaction of **9** and **10**, at reaction time 8 h. It is colorless microcrystals from ethanol, mp 120–122 °C, yield 69%. IR *ν* (cm^-1^): 1595, 1566, 1499. ^1^H-NMR δ (ppm): (400 MHz) 3.01 (dd, *J* = 7.1, 17.2 Hz, 1H, upfield H of pyrazolinyl *H_2_C*-4), 3.64 (s, 3H, O*CH_3_*), 3.86 (dd, *J* = 12.4, 17.2 Hz, 1H, downfield H of pyrazolinyl *H_2_C*-4), 3.92 (s, 3H, O*CH_3_*), 5.58 (dd, *J* = 7.1, 12.3 Hz, 1H, pyrazolinyl *HC*-5), 6.77-6.91 (m, 4H, arom. H), 7.06-7.11 (m, 4H, arom. H), 7.21 (t, *J* = 7.9 Hz, 2H, arom. H), 7.72 (dd, *J* = 5.5, 8.8 Hz, 2H, arom. H). Elemental analysis: C_23_H_21_FN_2_O_2_ required C, 73.39; H, 5.62; N, 7.44, found C, 73.45; H, 5.65; N, 7.49.

***2.1.20.*** *1-(4-Chlorophenyl)-5-(2,5-dimethoxyphenyl)-3-(4-fluorophenyl)-4,5-dihydro-1H-pyrazole (****32****)*

It is obtained from reaction of **9** and **11**, at reaction time 7 h. It is colorless microcrystals from n-butanol, mp 185–187 °C, yield 76%. IR *ν* (cm^-1^): 1595, 1558, 1493. ^1^H-NMR δ (ppm): (400 MHz) 3.03 (dd, *J* = 6.9, 17.2 Hz, 1H, upfield H of pyrazolinyl *H_2_C*-4), 3.65 (s, 3H, O*CH_3_*), 3.86 (dd, *J* = 12.3, 17.2 Hz, 1H, downfield H of pyrazolinyl *H_2_C*-4), 3.92 (s, 3H, O*CH_3_*), 5.55 (dd, *J* = 6.9, 12.3 Hz, 1H, pyrazolinyl *HC*-5), 6.71 (d, *J* = 3.0 Hz, 1H, arom. H), 6.78 (dd, *J* = 3.0, 8.8 Hz, 1H, arom. H), 6.90 (d, *J* = 8.8 Hz, 1H, arom. H), 6.98 (d, *J* = 9.0 Hz, 2H, arom. H), 7.09 (t, *J* = 8.7 Hz, 2H, arom. H), 7.15 (d, *J* = 9.0 Hz, 2H, arom. H), 7.71 (dd, *J* = 5.5, 8.7 Hz, 2H, arom. H). Elemental analysis: C_23_H_20_ClFN_2_O_2_ required C, 67.24; H, 4.91; N, 6.82, found C, 67.04; H, 4.80; N, 6.75.

***2.1.21. 5-(2,5-Dimethoxyphenyl)-3-(4-fluorophenyl)-1-(4-methylphenyl)-4,5-dihydro-1H-pyrazole (33)***

It is obtained from reaction of **9** and **12**, at reaction time 8 h. It is colorless microcrystals from n-butanol, mp 168–170 °C, yield 72%. IR *ν* (cm^-1^): 1616, 1597, 1560, 1521. ^1^H-NMR δ (ppm): (400 MHz) 2.28 (s, 3H, *CH_3_*), 3.00 (dd, *J* = 7.4, 17.1 Hz, 1H, upfield H of pyrazolinyl *H_2_C*-4), 3.66 (s, 3H, O*CH_3_*), 3.86 (dd, *J* = 12.4, 17.1 Hz, 1H, downfield H of pyrazolinyl *H_2_C*-4), 3.93 (s, 3H, O*CH_3_*), 5.55 (dd, *J* = 7.4, 12.3 Hz, 1H, pyrazolinyl *HC*-5), 6.77-6.81 (m, 2H, arom. H), 6.91 (d, *J* = 8.6 Hz, 1H, arom. H), 6.98-7.11 (m, 6H, arom. H), 7.72 (dd, *J* = 5.5, 8.7 Hz, 2H, arom. H). Elemental analysis: C_24_H_23_FN_2_O_2_ required C, 73.83; H, 5.94; N, 7.17, found C, 73.91; H, 5.70; N, 6.99.

**Table titles**

**Table S1.** Descriptor of the BMLR-QSPR model for the fluorescence active fluorinated pyrazolines (**13-33**)^.^

**Table S2.** Observed and estimated/predicted quantum yield values of the fluorescence active pyrazolines (**13-33**) according to the BMLR-QSPR model.

**Table S3.** Molecular descriptor values of the BMLR-QSPR model for the fluorescence active pyrazolines (**13‒33**) according to the BMLR-QSPR model.

**Table S4.** Descriptor of the BMLR-QSPR model for the fluorescence active subset group (A+B).

**Table S5.** Observed and estimated/predicted activity values for the subset group (A+B) according to the (A+B)-QSPR model.

**Table S6.** Observed and estimated/predicted activity values for the subset group (C, as an external test set) according to the (A+B)-QSPR model.

**Table S7.** Descriptor of the BMLR-QSPR model for the fluorescence active subset group (A+C).

**Table S8.** Observed and estimated/predicted activity values for the subset group (A+C) according to the (A+C)-QSPR model.

**Table S9.** Observed and estimated/predicted activity values for the subset group (B, as an external test set) according to the (A+C)-QSPR model.

**Table S10.** Descriptor of the BMLR-QSPR model for the fluorescence active subset group (B+C).

**Table S11.** Observed and estimated/predicted activity values for the subset group (B+C) according to the (B+C)-QSPR model.

**Table S12.** Observed and estimated/predicted activity values for the subset group (A, as an external test set) according to the (B+C)-QSPR model.

**Figure captions**

**Fig. S1.** IR spectrum of compound **13** (KBr pellet).

**Fig. S2.** ^1^H-NMR spectrum of compound **13** in CDCl_3_.

**Fig. S3.** ^13^C-NMR spectrum of compound **13** in CDCl_3_.

**Fig. S4.** IR spectrum of compound **14** (KBr pellet).

**Fig. S5.** ^1^H-NMR spectrum of compound **14** in CDCl_3_.

**Fig. S6.** IR spectrum of compound **15** (KBr pellet).

**Fig. S7.** ^1^H-NMR spectrum of compound **15** in CDCl_3_.

**Fig. S8.** ^13^C-NMR spectrum of compound **15** in CDCl_3_.

**Fig. S9.** IR spectrum of compound **16** (KBr pellet).

**Fig. S10.** ^1^H-NMR spectrum of compound **16** in CDCl_3_.

**Fig. S11.** IR spectrum of compound **17** (KBr pellet).

**Fig. S12.** ^1^H-NMR spectrum of compound **17** in CDCl_3_.

**Fig. S13.** IR spectrum of compound **18** (KBr pellet).

**Fig. S14.** ^1^H-NMR spectrum of compound **18** in CDCl_3_.

**Fig. S15.** ^13^C-NMR spectrum of compound **18** in CDCl_3_.

**Fig. S16.** IR spectrum of compound **19** (KBr pellet).

**Fig. S17.** ^1^H-NMR spectrum of compound **19** in CDCl_3_.

**Fig. S18.** IR spectrum of compound **20** (KBr pellet).

**Fig. S19.** ^1^H-NMR spectrum of compound **20** in CDCl_3_.

**Fig. S20.** IR spectrum of compound **21** (KBr pellet).

**Fig. S21.** ^1^H-NMR spectrum of compound **21** in CDCl_3_.

**Fig. S22.** IR spectrum of compound **22** (KBr pellet).

**Fig. S23.** ^1^H-NMR spectrum of compound **22** in CDCl_3_.

**Fig. S24.** IR spectrum of compound **23** (KBr pellet).

**Fig. S25.** ^1^H-NMR spectrum of compound **23** in CDCl_3_.

**Fig. S26.** ^13^C-NMR spectrum of compound **23** in CDCl_3_.

**Fig. S27.** IR spectrum of compound **24** (KBr pellet).

**Fig. S28.** ^1^H-NMR spectrum of compound **24** in CDCl_3_.

**Fig. S29.** IR spectrum of compound **25** (KBr pellet).

**Fig. S30.** ^1^H-NMR spectrum of compound **25** in CDCl_3_.

**Fig. S31.** IR spectrum of compound **26** (KBr pellet).

**Fig. S32.** ^1^H-NMR spectrum of compound **26** in CDCl_3_.

**Fig. S33.** IR spectrum of compound **27** (KBr pellet).

**Fig. S34.** ^1^H-NMR spectrum of compound **27** in CDCl_3_.

**Fig. S35.** IR spectrum of compound **28** (KBr pellet).

**Fig. S36.** ^1^H-NMR spectrum of compound **28** in CDCl_3_.

**Fig. S37.** IR spectrum of compound **29** (KBr pellet).

**Fig. S38.** ^1^H-NMR spectrum of compound **29** in CDCl_3_.

**Fig. S39.** IR spectrum of compound **30** (KBr pellet).

**Fig. S40.** ^1^H-NMR spectrum of compound **30** in CDCl_3_.

**Fig. S41.** IR spectrum of compound **31** (KBr pellet).

**Fig. S42.** ^1^H-NMR spectrum of compound **31** in CDCl_3_.

**Fig. S43.** IR spectrum of compound **32** (KBr pellet).

**Fig. S44.** ^1^H-NMR spectrum of compound **32** in CDCl_3_.

**Fig. S45.** IR spectrum of compound **33** (KBr pellet).

**Fig. S46.** ^1^H-NMR spectrum of compound **33** in CDCl_3_.

**Fig. S47**. BMLR-QSPR model plot of correlations representing the observed *versus* predicted quantum yield (Ф_s_) values for the synthesized pyrazolines **13-33**.

**Fig. S48.** BMLR-QSPR model plot of correlations representing the observed *versus* predicted 1/log(quantum yield, ɸ_s_) values for the subset group (A+B).

**Fig. S49.** BMLR-QSPR model plot of correlations representing the observed *versus* predicted quantum yield (Ф_s_ _s_) values for the subset group (A+C).

**Fig. S50.** BMLR-QSPR model plot of correlations representing the observed *versus* predicted quantum yield (ɸ_s_) values for the subset group (B+C), compound **14** is an outliar.

**Table S1.**Descriptor of the BMLR-QSPR model for the fluorescence active fluorinated pyrazolines (**13-33**)^a^

| Entry | ID | Coefficient | *s* | *t* | Descriptor |
| --- | --- | --- | --- | --- | --- |
| 1 | 0 | 41.8763 | 6.981 | 5.998 | Intercept |
| 2 | D_1_ | 1.74869 | 0.236 | 7.403 | LUMO+1 energy |
| 3 | D_2_ | -0.342689 | 0.082 | -4.199 | Number of Br atoms |
| 4 | D_3_ | -9.96951 | 1.777 | -5.609 | Max. coulombic interaction for bond H-C |
| 5 | D_4_ | -165.672 | 23.466 | -7.060 | Positively charged part of partial charged surface area (MOPAC PC) |
| ^a^*N* = 21, *n* = 4, *R*^2^ = 0.817, *R*^2^cvOO = 0.708, *R*^2^cvMO = 0.730, *F* = 17.881, *s*^2^ = 0.011  Quantum yield (ɸ_s_) = 41.8763 + (1.74869 x *D*_1_) ‒ (0.342689 x *D*_2_) ‒ (9.96951 x *D*_3_) ‒ (165.672 X *D*_4_) | | | | | |

**Table S2.** Observed and estimated/predicted quantum yield values of the fluorescence active pyrazolines (**13-33**) according to the BMLR-QSPR model.

| Entry | Compd. | Observed Ф_s_^a^ | Estimated Ф_s_ | Error^b^ |
| --- | --- | --- | --- | --- |
| 1 | **13** | 0.819 | 0.704 | 0.115 |
| 2 | **14** | 0.490 | 0.420 | 0.070 |
| 3 | **15** | 0.858 | 0.905 | -0.047 |
| 4 | **16** | 0.155 | 0.310 | -0.155 |
| 5 | **17** | 0.455 | 0.355 | 0.100 |
| 6 | **18** | 0.553 | 0.742 | -0.189 |
| 7 | **19** | 0.802 | 0.686 | 0.116 |
| 8 | **20** | 0.546 | 0.529 | 0.017 |
| 9 | **21** | 0.269 | 0.250 | 0.019 |
| 10 | **22** | 0.473 | 0.422 | 0.051 |
| 11 | **23** | 0.844 | 0.806 | 0.038 |
| 12 | **24** | 0.521 | 0.607 | -0.086 |
| 13 | **25** | 0.267 | 0.226 | 0.041 |
| 14 | **26** | 0.333 | 0.398 | -0.065 |
| 15 | **27** | 0.335 | 0.261 | 0.074 |
| 16 | **28** | 0.210 | 0.282 | -0.072 |
| 17 | **29** | 0.324 | 0.303 | 0.021 |
| 18 | **30** | 0.271 | 0.389 | -0.118 |
| 19 | **31** | 0.297 | 0.403 | -0.106 |
| 20 | **32** | 0.628 | 0.535 | 0.093 |
| 21 | **33** | 0.588 | 0.501 | 0.087 |

^a^ Measured in chloroform.

^b^Error is the difference between the observed and estimated quantum yield values.

**Table S3.** Molecular descriptor values of the BMLR-QSPR model for the fluorescence active pyrazolines (**13‒33**) according to the BMLR-QSPR model.

| Entry | Compd. | Descriptors^a^ | | | |
| --- | --- | --- | --- | --- | --- |
|  |  | *D*_1_ | *D*_2_ | *D*_3_ | *D*_4_ |
| 1 | **13** | -0.227 | 0 | 3.8563 | 0.01407 |
| 2 | **14** | -0.242 | 1 | 3.8563 | 0.01355 |
| 3 | **15** | -0.074 | 0 | 3.862 | 0.01412 |
| 4 | **16** | -0.067 | 0 | 3.8846 | 0.01643 |
| 5 | **17** | -0.162 | 0 | 3.8887 | 0.01491 |
| 6 | **18** | -0.265 | 0 | 3.8574 | 0.01336 |
| 7 | **19** | -0.318 | 0 | 3.8558 | 0.01324 |
| 8 | **20** | -0.252 | 1 | 3.8633 | 0.01236 |
| 9 | **21** | -0.155 | 0 | 3.8864 | 0.01575 |
| 10 | **22** | -0.187 | 0 | 3.8989 | 0.01362 |
| 11 | **23** | -0.195 | 0 | 3.8622 | 0.01343 |
| 12 | **24** | -0.152 | 1 | 3.863 | 0.01297 |
| 13 | **25** | -0.12 | 0 | 3.8817 | 0.01655 |
| 14 | **26** | -0.162 | 0 | 3.891 | 0.01451 |
| 15 | **27** | -0.526 | 0 | 3.8616 | 0.01326 |
| 16 | **28** | -0.451 | 0 | 3.8596 | 0.01405 |
| 17 | **29** | -0.556 | 0 | 3.86 | 0.01279 |
| 18 | **30** | -0.451 | 0 | 3.8579 | 0.01351 |
| 19 | **31** | 0 | 0 | 3.8556 | 0.01832 |
| 20 | **32** | 0.007 | 0 | 3.8606 | 0.01729 |
| 21 | **33** | 0.012 | 0 | 3.8553 | 0.01787 |

^a^*D*_1_ = LUMO+1 energy, *D*_2_ = Number of Br atoms, *D*_3_ = Max. coulombic interaction for bond H-C, *D*_4_ = Positively charged part of partial charged surface area (MOPAC PC).

**Table S4.** Descriptor of the BMLR-QSPR model for the fluorescence active subset group (A+B).

| ID | Coefficient | *s* | *t* | Descriptor |
| --- | --- | --- | --- | --- |
| 0 | -62.5041 | 13.087 | -4.776 | Intercept |
| *D_1_* | 0.00999751 | 0.002 | 5.132 | 1X BETA polarizability (DIP) |
| *D_2_* | 7.77697 | 1.800 | 4.321 | HA dependent HDCA-1 (Zefirov PC) |
| *D_3_* | 6.91133 | 1.693 | 4.082 | Kier & Hall index (order 2) |
| *N* = 14, *n* = 3, *R*^2^ = 0.820, *R*^2^cvOO = 0.703, *R*^2^cvMO = 0.726, *F* = 15.221, *s*^2^ = 4.908  1/log (ɸ_s_) = -62.5041 + (0.00999751 x *D_1_*)+ (7.77697 x *D_2_*) + (6.91133 x *D_3_*) | | | | |

**Table S5.** Observed and estimated/predicted activity values for the subset group (A+B) according to the (A+B)-QSPR model.

| Entry | Compd. | Observed ɸ_s_^a^ | Estimated ɸ_s_ | Error^b^ |
| --- | --- | --- | --- | --- |
| 1 | **14** | 0.490 | 0.388 | 0.102 |
| 2 | **15** | 0.858 | 0.859 | -0.001 |
| 3 | **18** | 0.553 | 0.636 | -0.083 |
| 4 | **19** | 0.802 | 0.703 | 0.099 |
| 5 | **20** | 0.546 | 0.750 | -0.204 |
| 6 | **22** | 0.473 | 0.549 | -0.076 |
| 7 | **23** | 0.844 | 0.819 | 0.025 |
| 8 | **25** | 0.267 | 0.399 | -0.132 |
| 9 | **26** | 0.333 | 0.207 | 0.126 |
| 10 | **27** | 0.335 | 0.250 | 0.085 |
| 11 | **28** | 0.210 | 0.351 | -0.141 |
| 12 | **30** | 0.271 | 0.063 | 0.208 |
| 13 | **31** | 0.297 | 0.572 | -0.275 |
| 14 | **32** | 0.628 | 0.530 | 0.098 |

^a^ Measured in chloroform.

^b^Error is the difference between the observed and estimated quantum yield values.

**Table S6.** Observed and estimated/predicted activity values for the subset group (C, as an external test set) according to the (A+B)-QSPR model.

| Entry | Compd. | Observed ɸ_s_^a^ | Estimated ɸ_s_ | Error^b^ |
| --- | --- | --- | --- | --- |
| 1 | **13** | 0.819 | 0.782 | 0.037 |
| 2 | **16** | 0.155 | 0.745 | -0.590 |
| 3 | **17** | 0.455 | 0.651 | -0.196 |
| 4 | **21** | 0.269 | 0.060 | 0.209 |
| 5 | **24** | 0.521 | 0.720 | -0.199 |
| 6 | **29** | 0.324 | 0.560 | -0.236 |
| 7 | **33** | 0.588 | 0.168 | 0.420 |

^a^ Measured in chloroform.

^b^Error is the difference between the observed and estimated quantum yield values.

**Table S7.** Descriptor of the BMLR-QSPR model for the fluorescence active subset group (A+C).

| ID | Coefficient | *s* | *t* | Descriptor |
| --- | --- | --- | --- | --- |
| 0 | -0.349118 | 0.618 | -0.565 | Intercept |
| *D_1_* | 0.0755456 | 0.013 | 5.659 | RNCS Relative negative charged SA (SAMNEG*RNCG) (Zefirov PC) |
| *D_2_* | 0.046297 | 0.011 | 4.070 | Vib heat capacity (300K) |
| *D_3_* | -0.70857 | 0.164 | -4.332 | Kier & Hall index (order 3) |
| *N* = 14, *n* = 3, *R*^2^ = 0.786, *R*^2^cvOO = 0.631, *R*^2^cvMO = 0.659, *F* = 12.210, *s*^2^ = 0.014  ɸ_s_ = -0.349118 + (0.0755456 x *D_1_*)+ (0.046297 x *D_2_*) ‒ (0.70857 x *D_3_*) | | | | |

**Table S8.** Observed and estimated/predicted activity values for the subset group (A+C) according to the (A+C)-QSPR model.

| Entry | Compd. | Observed ɸ_s_^a^ | Estimated ɸ_s_ | Error^b^ |
| --- | --- | --- | --- | --- |
| 1 | **13** | 0.819 | 0.751 | 0.068 |
| 2 | **14** | 0.490 | 0.607 | -0.117 |
| 3 | **15** | 0.858 | 0.890 | -0.032 |
| 4 | **16** | 0.155 | 0.228 | -0.073 |
| 5 | **17** | 0.455 | 0.395 | 0.060 |
| 6 | **18** | 0.553 | 0.476 | 0.077 |
| 7 | **19** | 0.802 | 0.589 | 0.213 |
| 8 | **21** | 0.269 | 0.181 | 0.088 |
| 9 | **24** | 0.521 | 0.659 | -0.138 |
| 10 | **25** | 0.267 | 0.309 | -0.042 |
| 11 | **27** | 0.335 | 0.417 | -0.082 |
| 12 | **29** | 0.324 | 0.367 | -0.043 |
| 13 | **31** | 0.297 | 0.395 | -0.098 |
| 14 | **33** | 0.588 | 0.469 | 0.119 |

^a^ Measured in chloroform.

^b^Error is the difference between the observed and estimated quantum yield values.

**Table S9.** Observed and estimated/predicted activity values for the subset group (B, as an external test set) according to the (A+C)-QSPR model.

| Entry | Compd. | Observed ɸ_s_^a^ | Estimated ɸ_s_ | Error^b^ |
| --- | --- | --- | --- | --- |
| 1 | **20** | 0.546 | 0.469 | 0.077 |
| 2 | **22** | 0.473 | 0.223 | 0.250 |
| 3 | **23** | 0.844 | 0.641 | 0.203 |
| 4 | **26** | 0.333 | 0.400 | -0.067 |
| 5 | **28** | 0.210 | 0.577 | -0.367 |
| 6 | **30** | 0.271 | 0.524 | -0.253 |
| 7 | **32** | 0.628 | 0.319 | 0.309 |

^a^ Measured in chloroform.

^b^Error is the difference between the observed and estimated quantum yield va

**Table S10.** Descriptor of the BMLR-QSPR model for the fluorescence active subset group (B+C).

| ID | Coefficient | *s* | *t* | Descriptor |
| --- | --- | --- | --- | --- |
| 0 | -132.432 | 17.515 | -7.561 | Intercept |
| *D_1_* | 38.8202 | 4.875 | 7.963 | Min. valency for atom C |
| *D_2_* | 81.1984 | 12.085 | 6.719 | Min. 1-electron react. index for atom C |
| *D_3_* | -0.254788 | 0.0480 | -5.309 | Max. e-n attraction for bond H-C |
| *N* = 14, *n* = 3, *R*^2^ = 0.910, *R*^2^cvOO = 0.830, *R*^2^cvMO = 0.845, *F* = 33.770, *s*^2^ = 0.005  ɸ_s_ = -132.432 + (38.8202 x *D_1_*)+ (81.1984 x *D_2_*) ‒ (0.254788 x *D_3_*) | | | | |

**Table S11.** Observed and estimated/predicted activity values for the subset group (B+C) according to the (B+C)-QSPR model.

| Entry | Compd. | Observed ɸ_s_^a^ | Estimated ɸ_s_ | Error^b^ |
| --- | --- | --- | --- | --- |
| 1 | **13** | 0.819 | 0.793 | 0.026 |
| 2 | **16** | 0.155 | 0.089 | 0.066 |
| 3 | **17** | 0.455 | 0.402 | 0.053 |
| 4 | **20** | 0.546 | 0.573 | -0.027 |
| 5 | **21** | 0.269 | 0.202 | 0.067 |
| 6 | **22** | 0.473 | 0.480 | -0.007 |
| 7 | **23** | 0.844 | 0.804 | 0.040 |
| 8 | **24** | 0.521 | 0.471 | 0.050 |
| 9 | **26** | 0.333 | 0.482 | -0.149 |
| 10 | **28** | 0.210 | 0.263 | -0.053 |
| 11 | **29** | 0.324 | 0.394 | -0.070 |
| 12 | **30** | 0.271 | 0.329 | -0.058 |
| 13 | **32** | 0.628 | 0.618 | 0.010 |
| 14 | **33** | 0.588 | 0.536 | 0.052 |

^a^ Measured in chloroform.

^b^Error is the difference between the observed and estimated quantum yield values.

**Table S12.** Observed and estimated/predicted activity values for the subset group (A, as an external test set) according to the (B+C)-QSPR model.

| Entry | Compd. | Observed ɸ_s_^a^ | Estimated ɸ_s_ | Error^b^ |
| --- | --- | --- | --- | --- |
| 1 | **14** | 0.490 | 0.804 | -0.314 |
| 2 | **15** | 0.858 | 0.446 | 0.412 |
| 3 | **18** | 0.553 | 0.307 | 0.246 |
| 4 | **19** | 0.802 | 0.276 | 0.526 |
| 5 | **25** | 0.267 | 0.131 | 0.136 |
| 6 | **27** | 0.335 | 0.315 | 0.020 |
| 7 | **31** | 0.297 | 0.595 | -0.298 |

^a^ Measured in chloroform.

^b^Error is the difference between the observed and estimated quantum yield values.


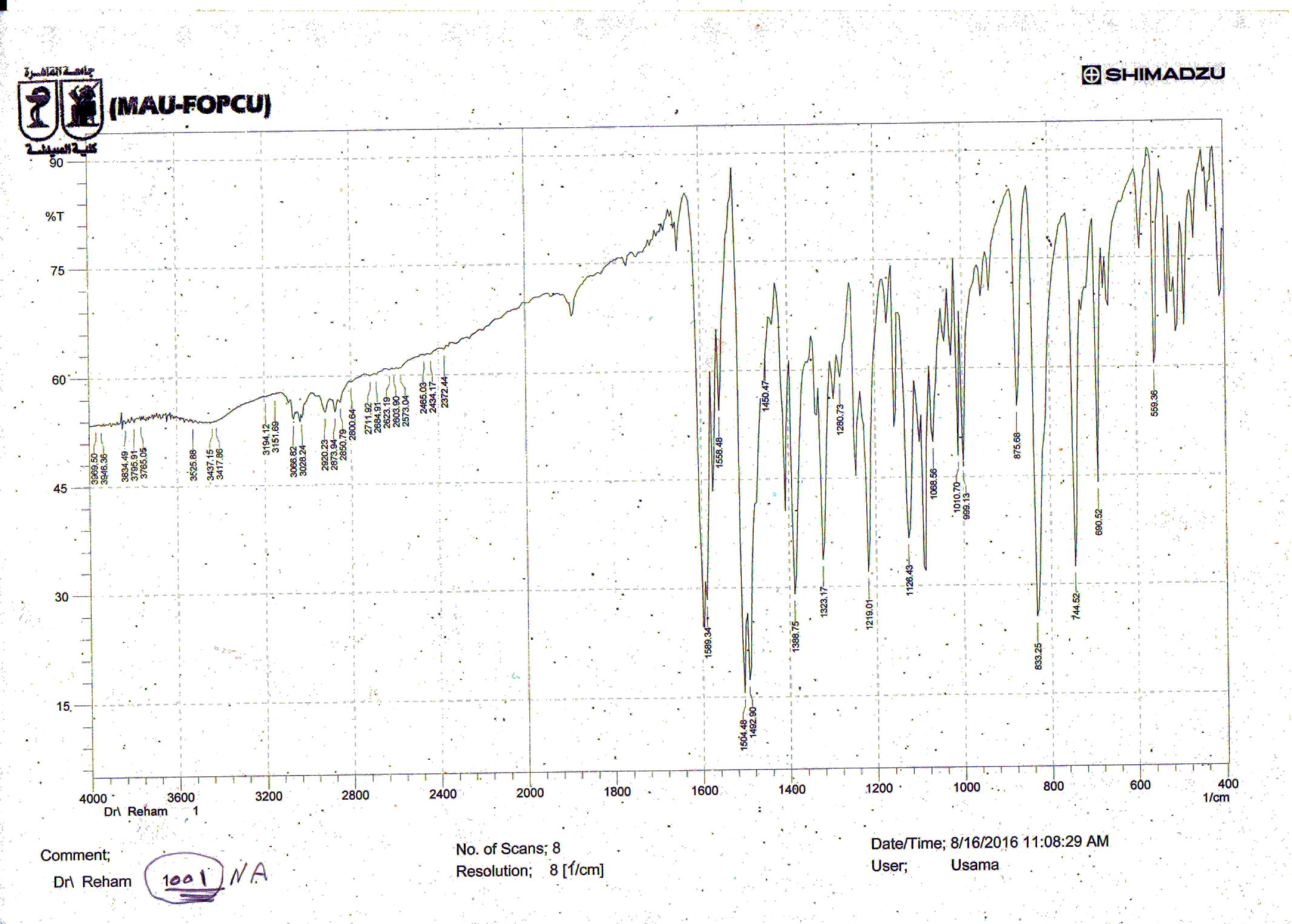


**Fig. S1.** IR spectrum of compound **13** (KBr pellet).

**Fig. S2.** 1H-NMR spectrum of compound **13** in CDCl3.


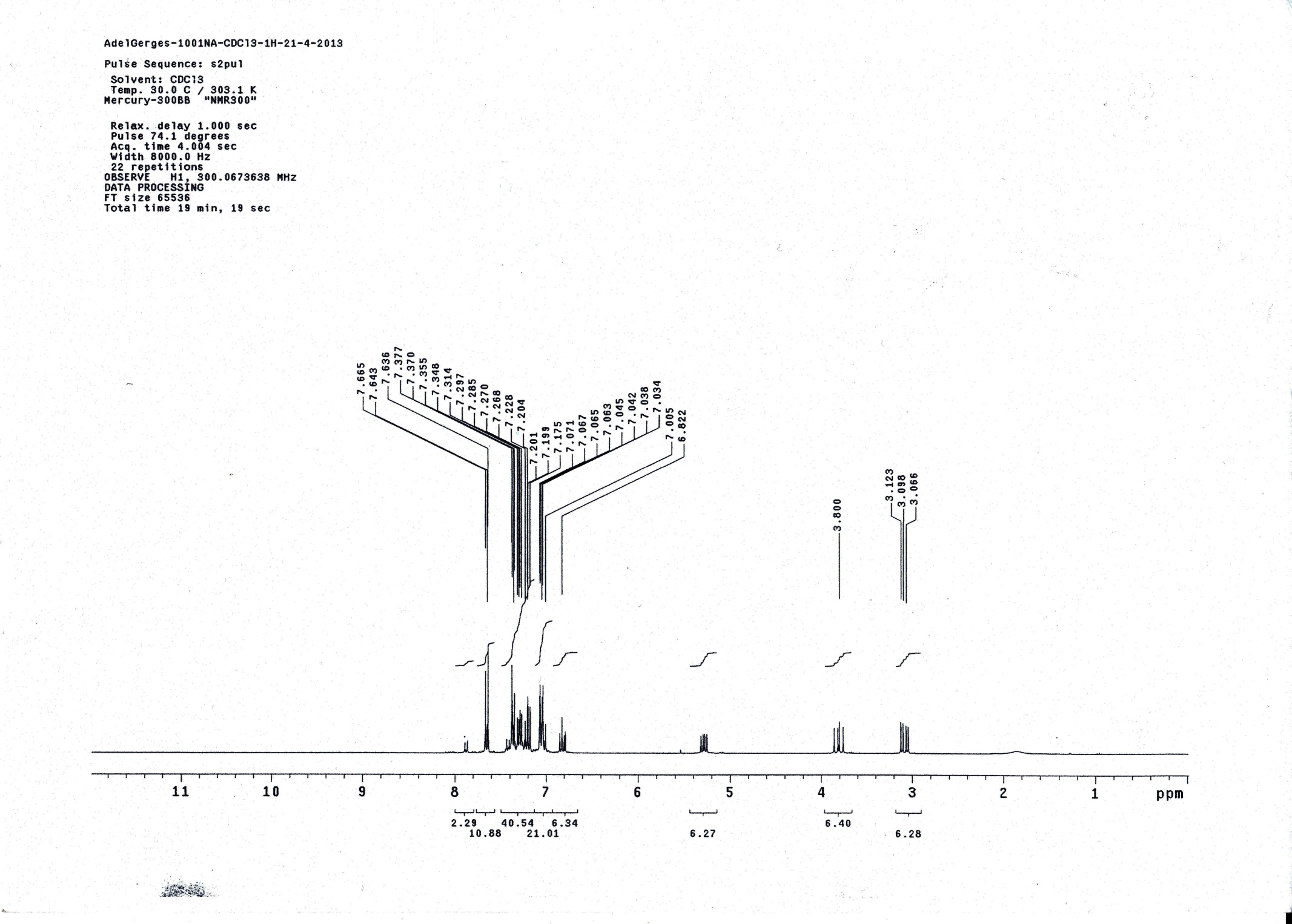

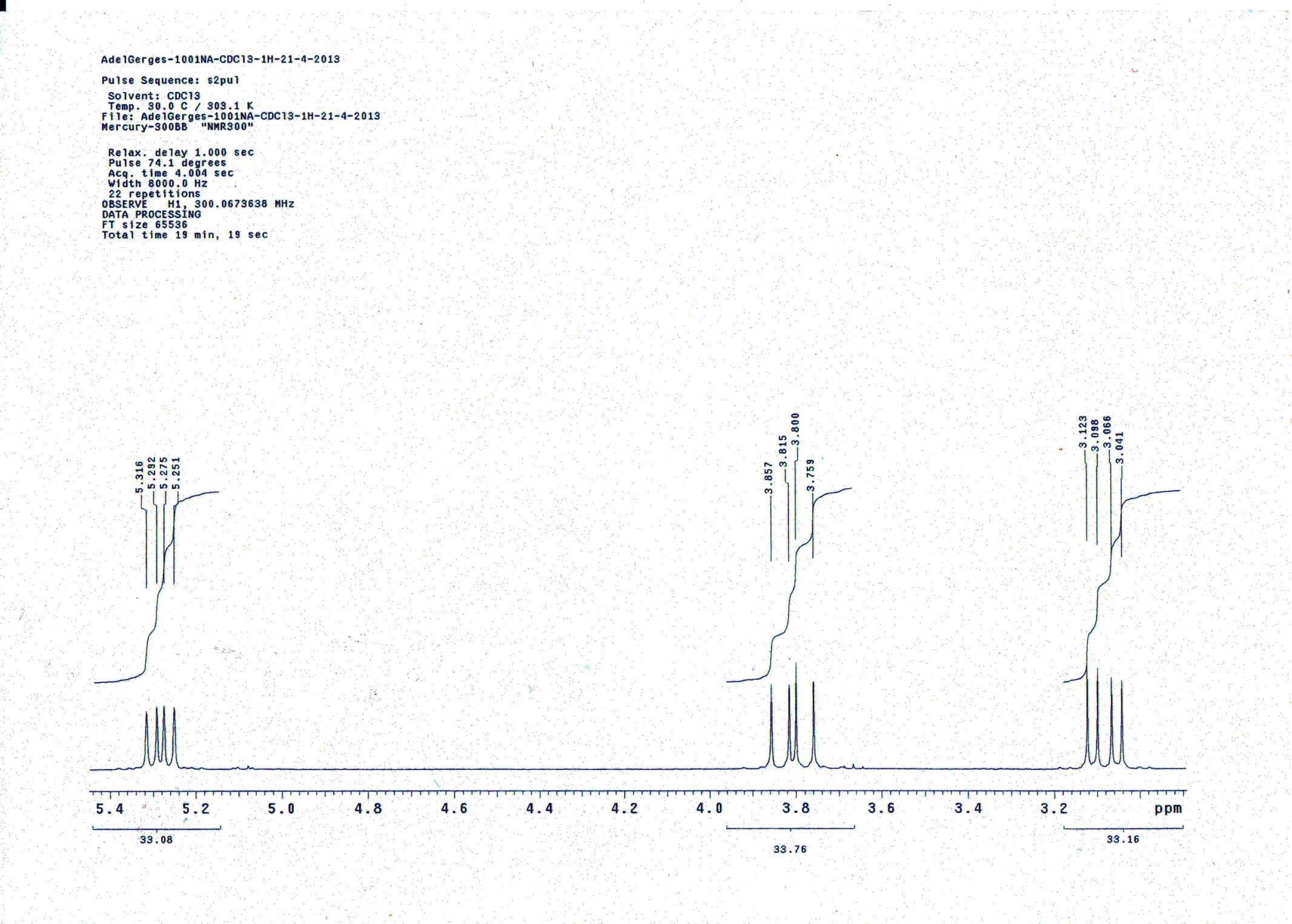


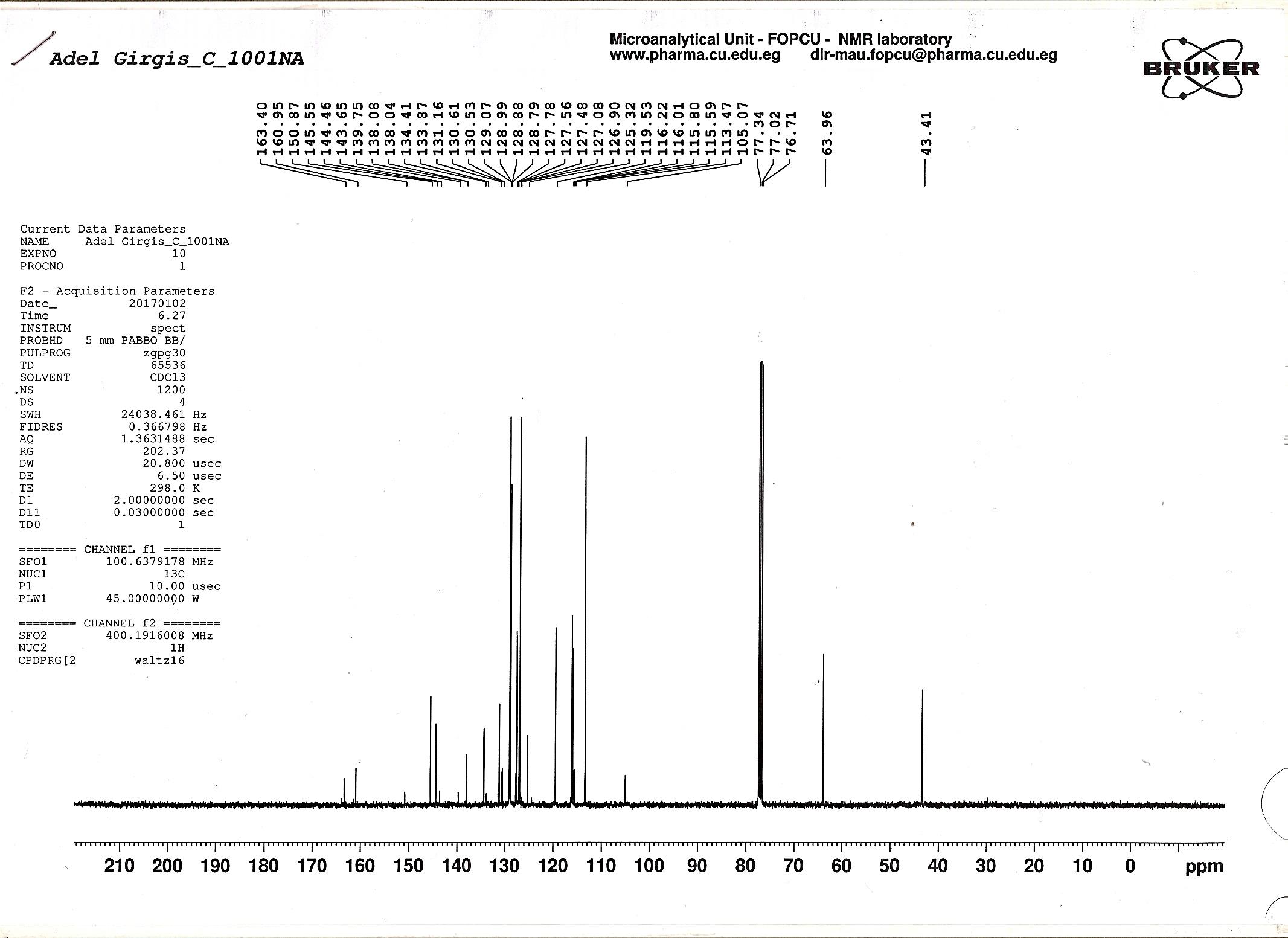


**Fig. S3.** 13C-NMR spectrum of compound **13** in CDCl3.


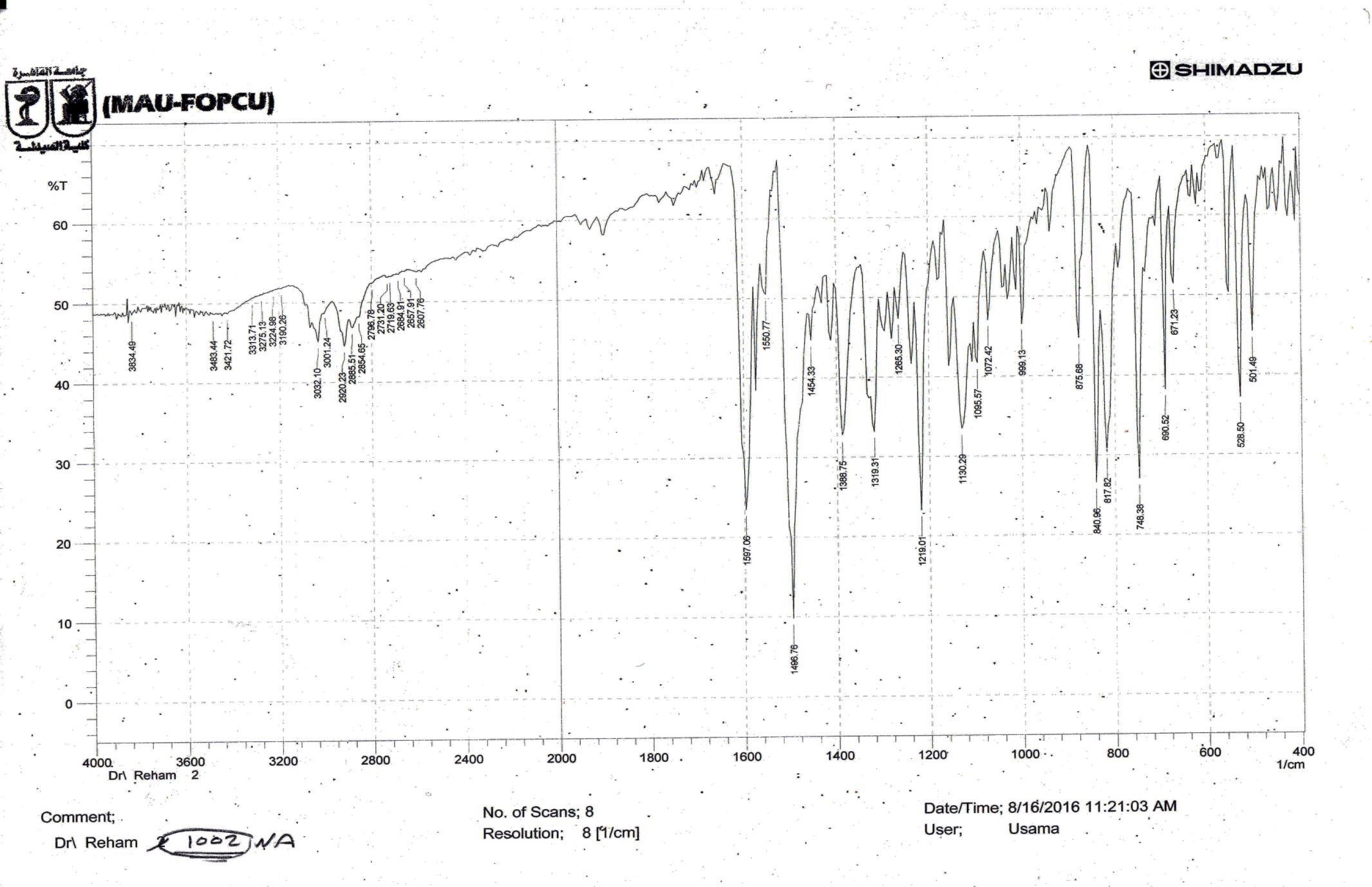


**Fig. S4.** IR spectrum of compound **14** (KBr pellet).


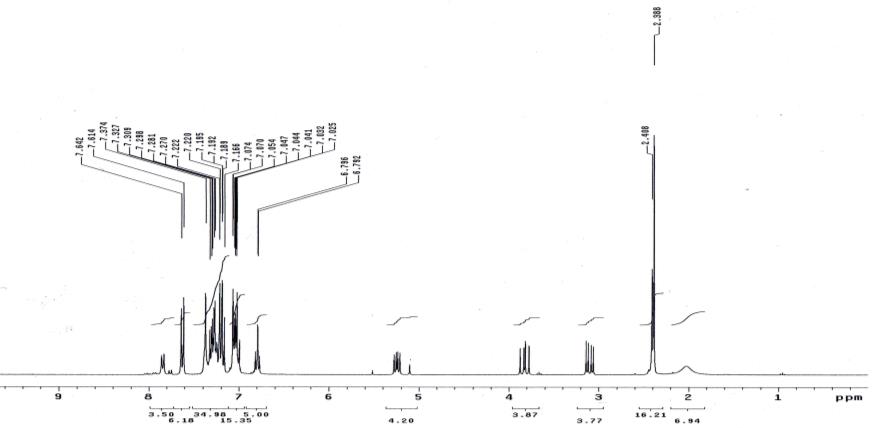

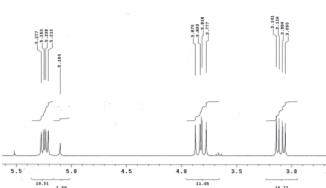


**Fig. S5.** 1H-NMR spectrum of compound **14** in CDCl3


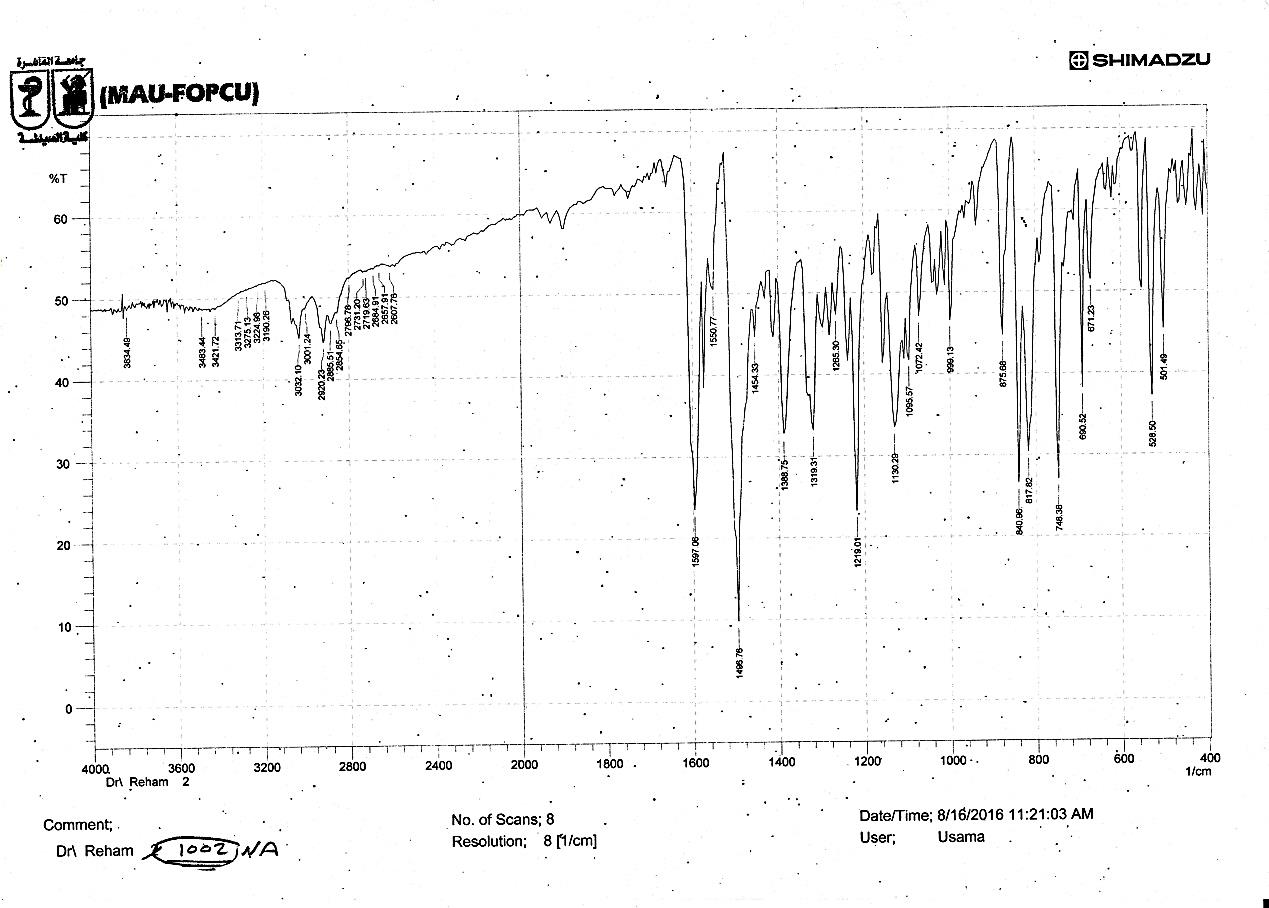


**Fig. S6.** IR spectrum of compound **15** (KBr pellet).

**Fig. S7.** 1H-NMR spectrum of compound **15** in CDCl3.


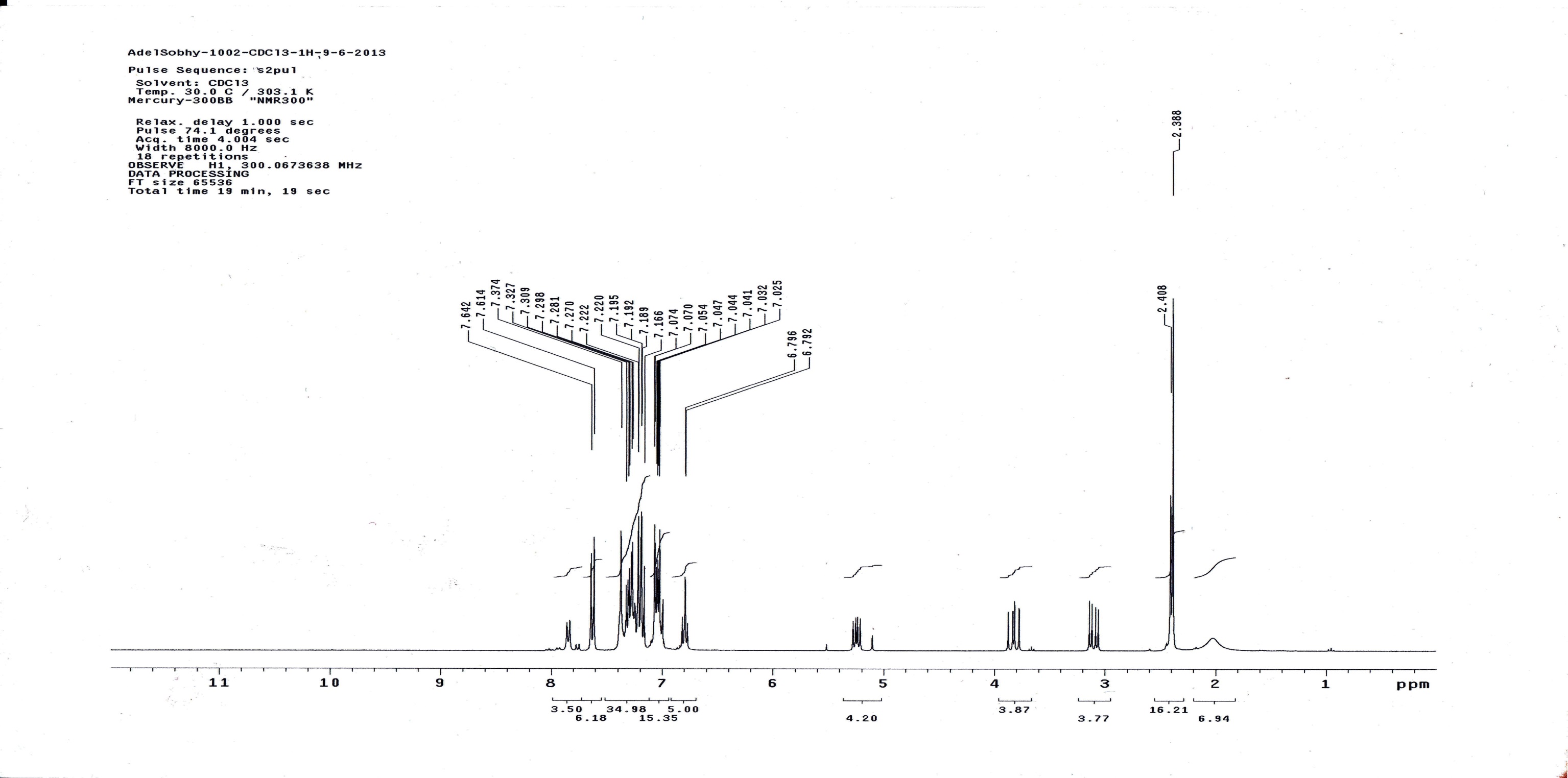

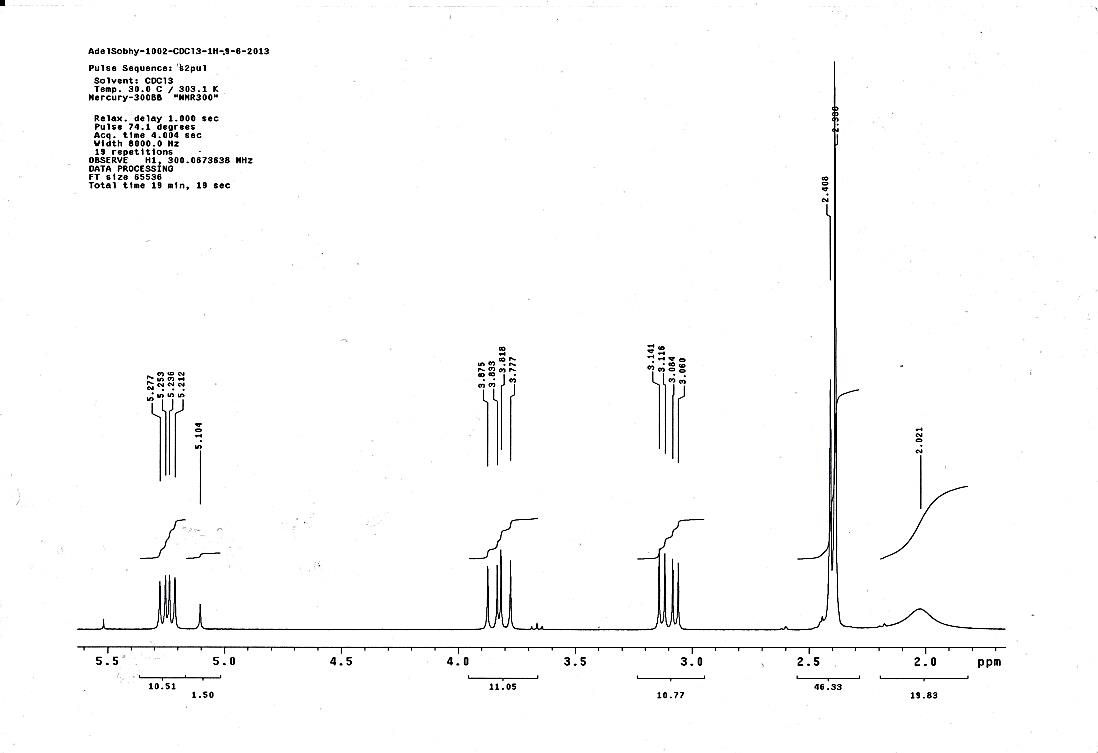


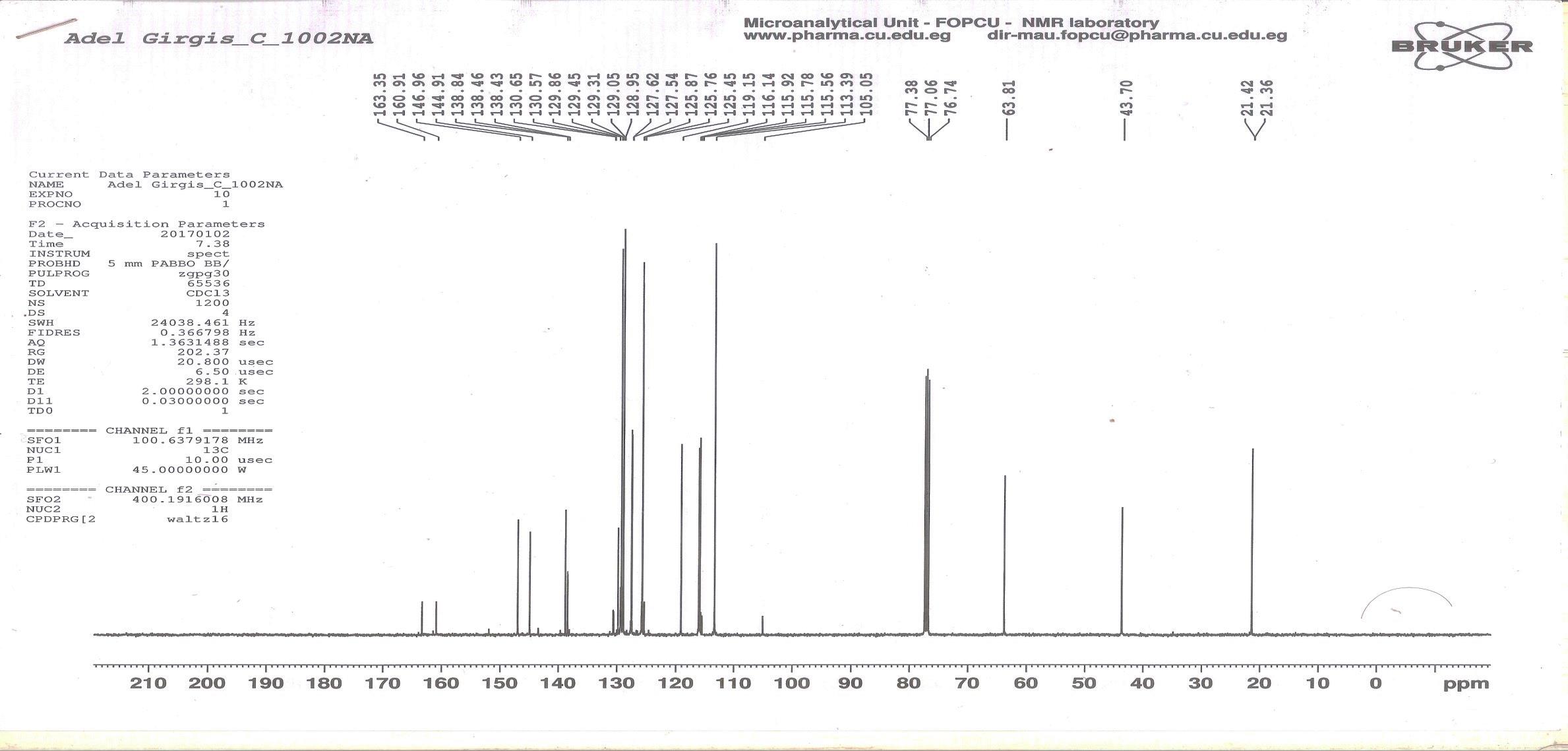


**Fig. S8.** 13C-NMR spectrum of compound **15** in CDCl3.


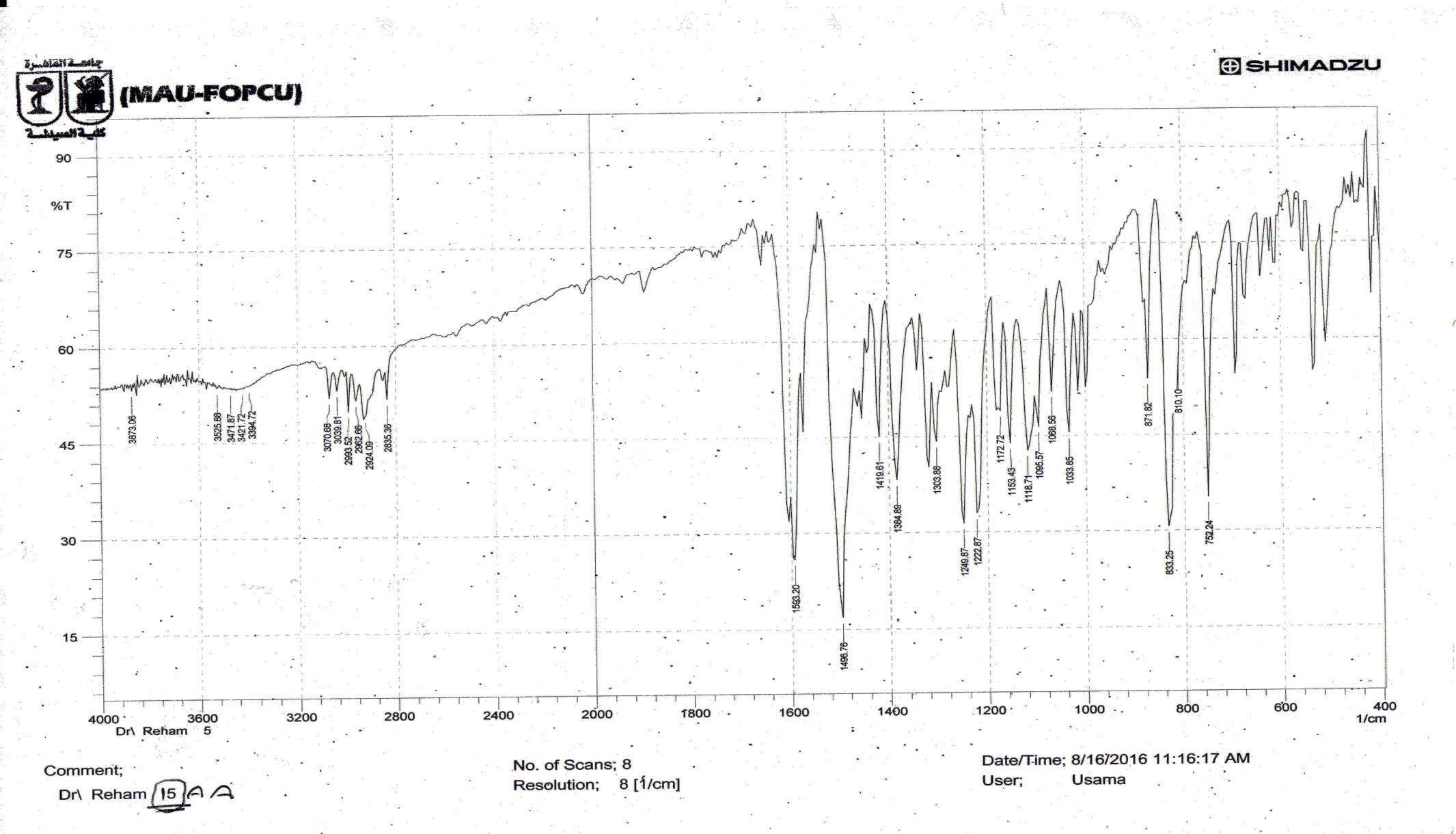


**Fig. S9.** IR spectrum of compound **16** (KBr pellet).


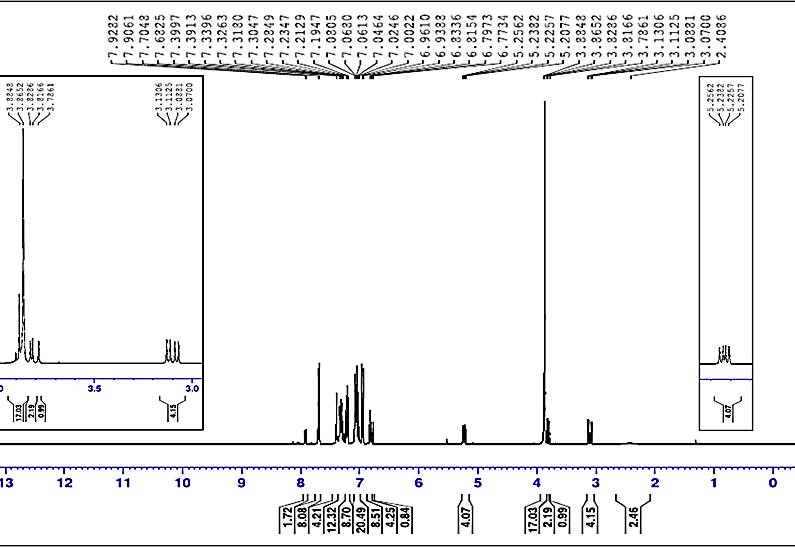


**Fig. S10.** 1H-NMR spectrum of compound **16** in CDCl3.


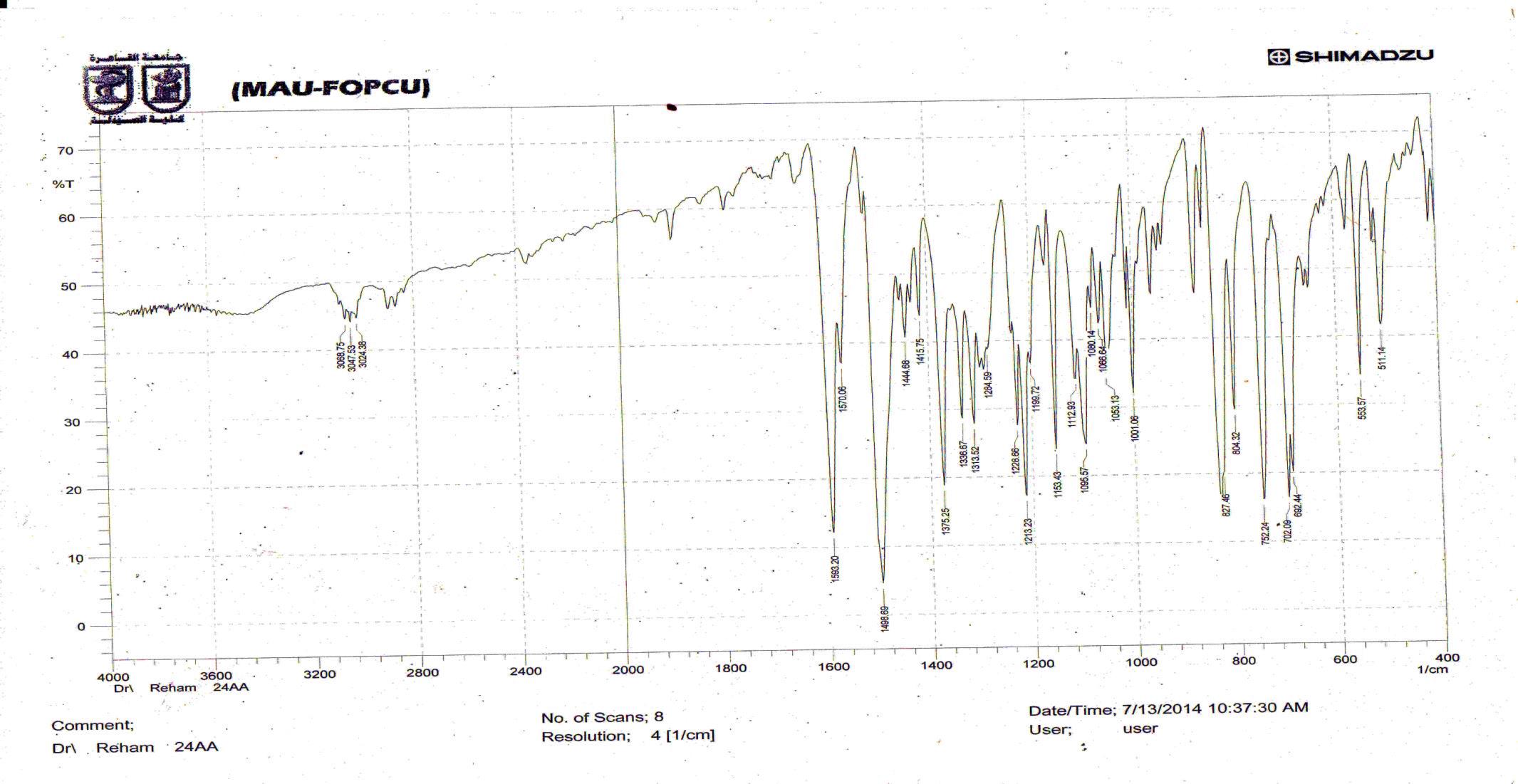


**Fig. S11.** IR spectrum of compound **17** (KBr pellet).


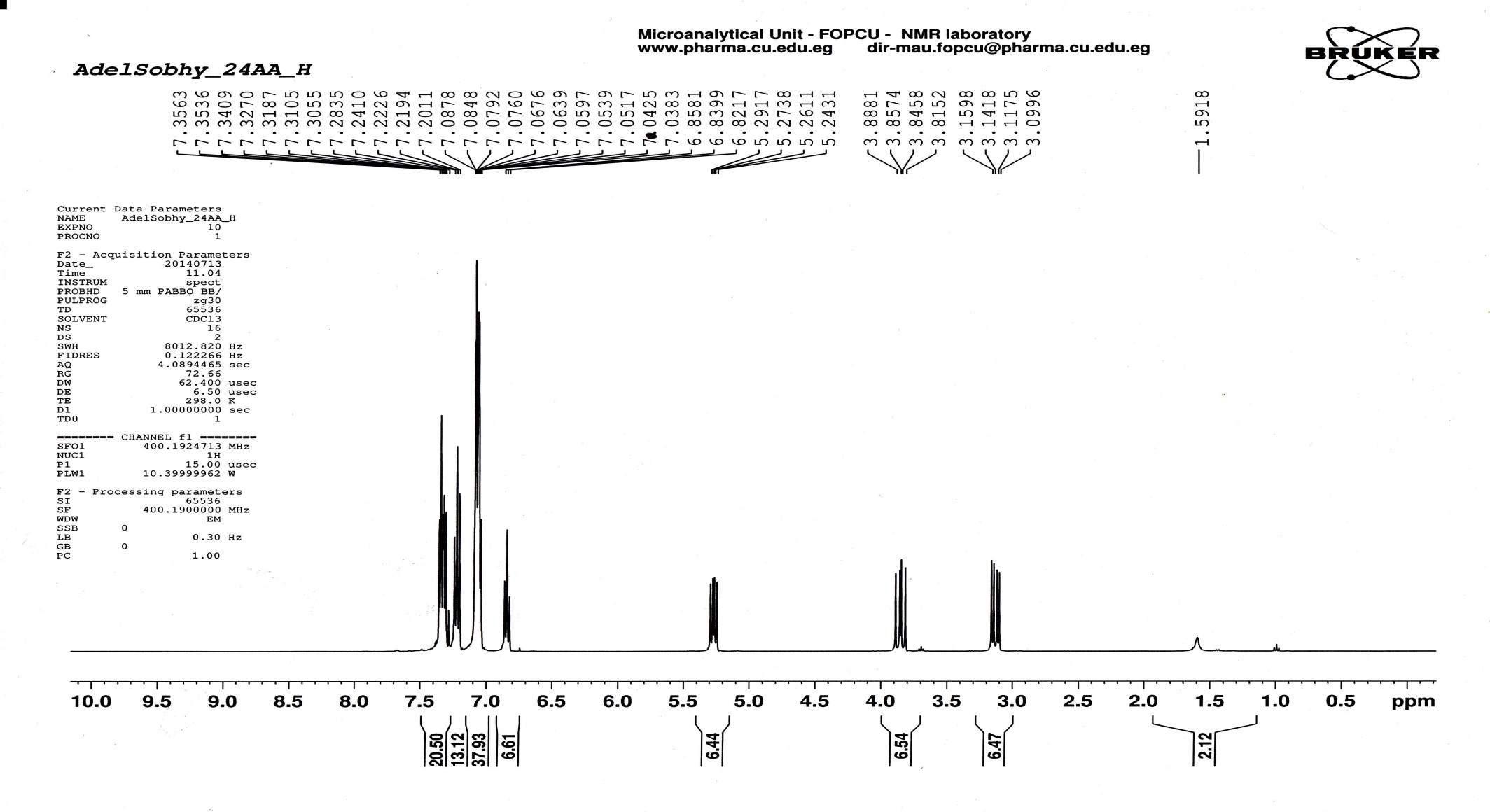


**Fig. S12.** 1H-NMR spectrum of compound **17** in CDCl3.


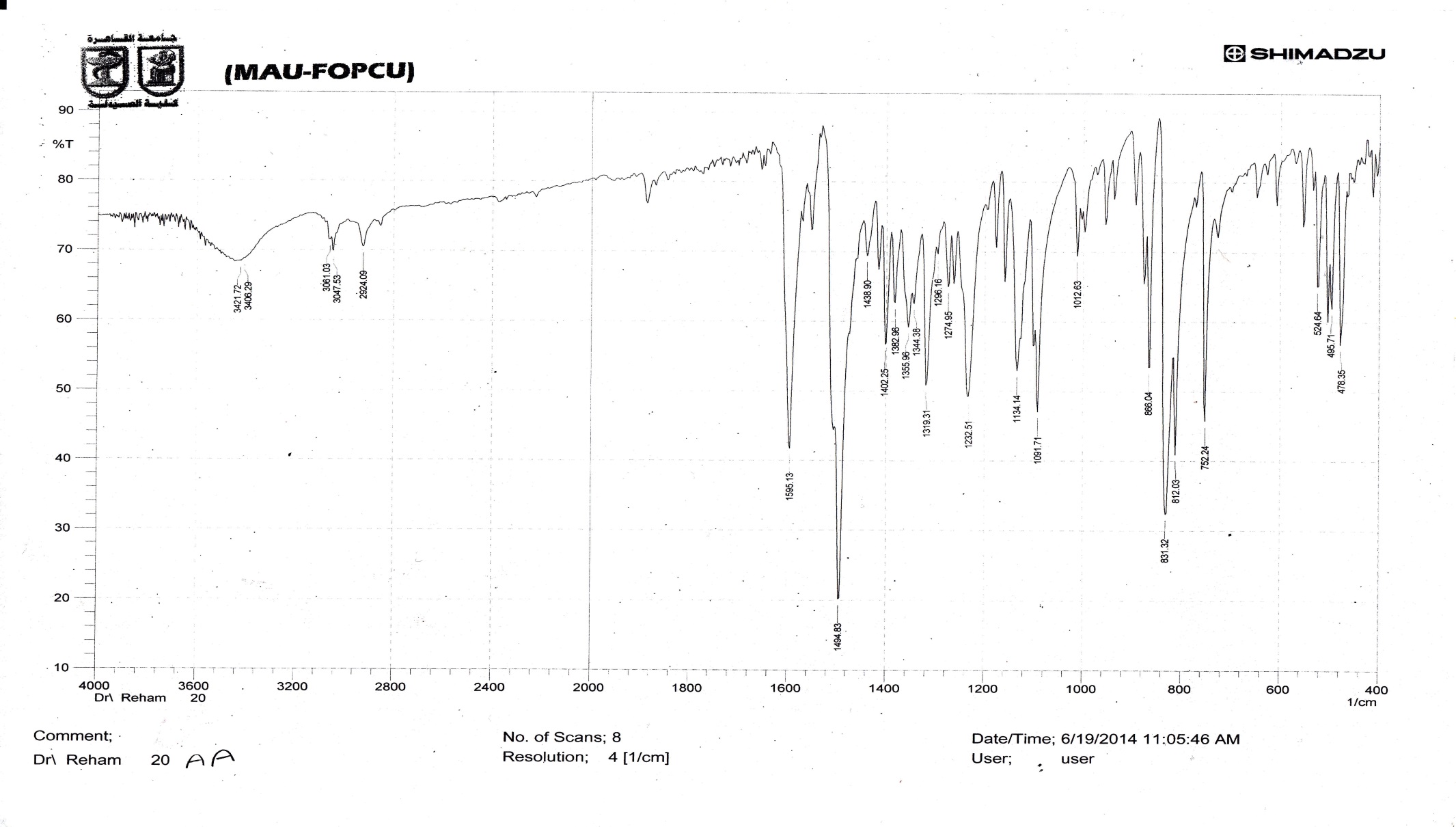


**Fig. S13.** IR spectrum of compound **18** (KBr pellet).


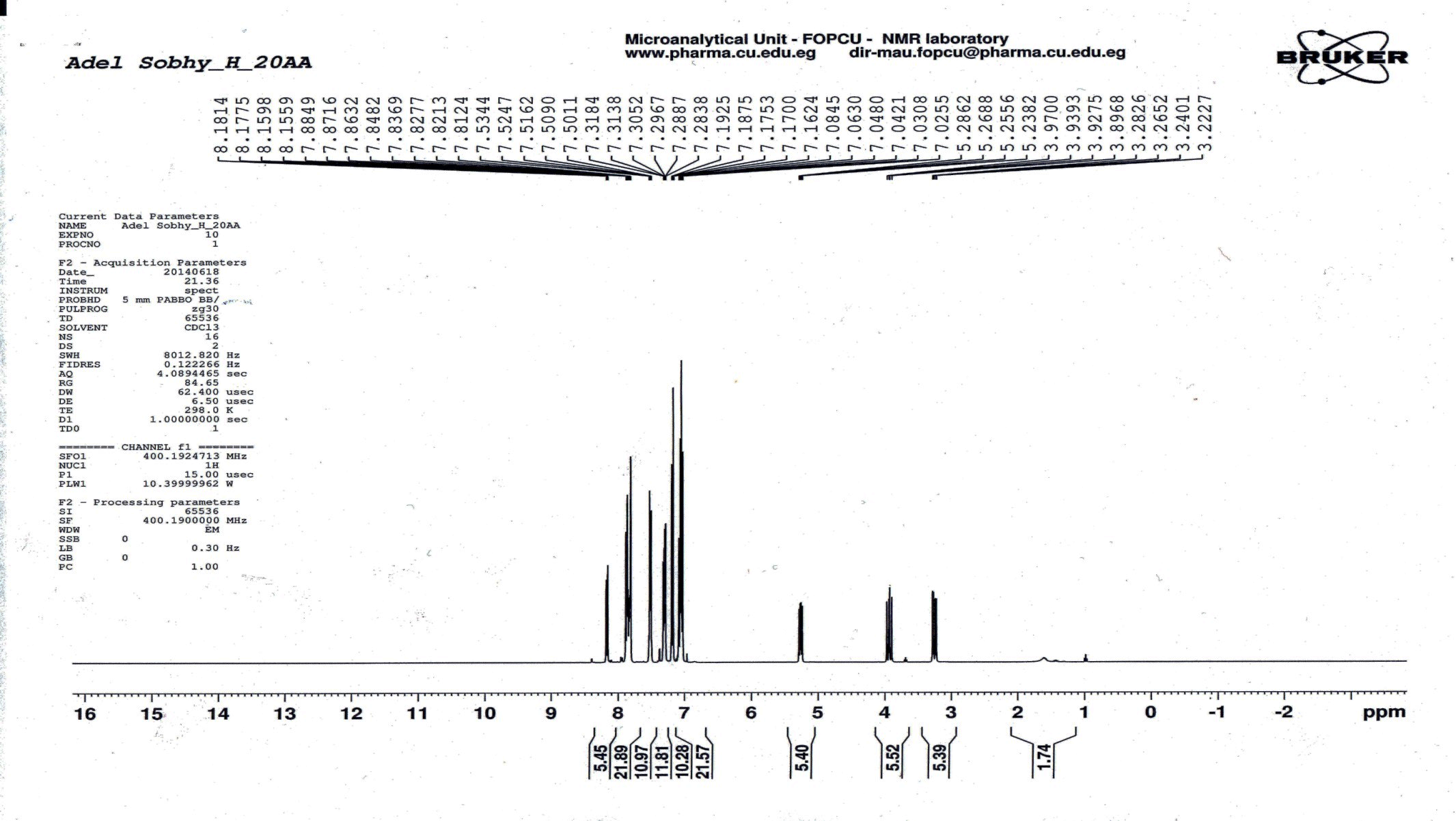


**Fig. S14.** 1H-NMR spectrum of compound **18** in CDCl3.


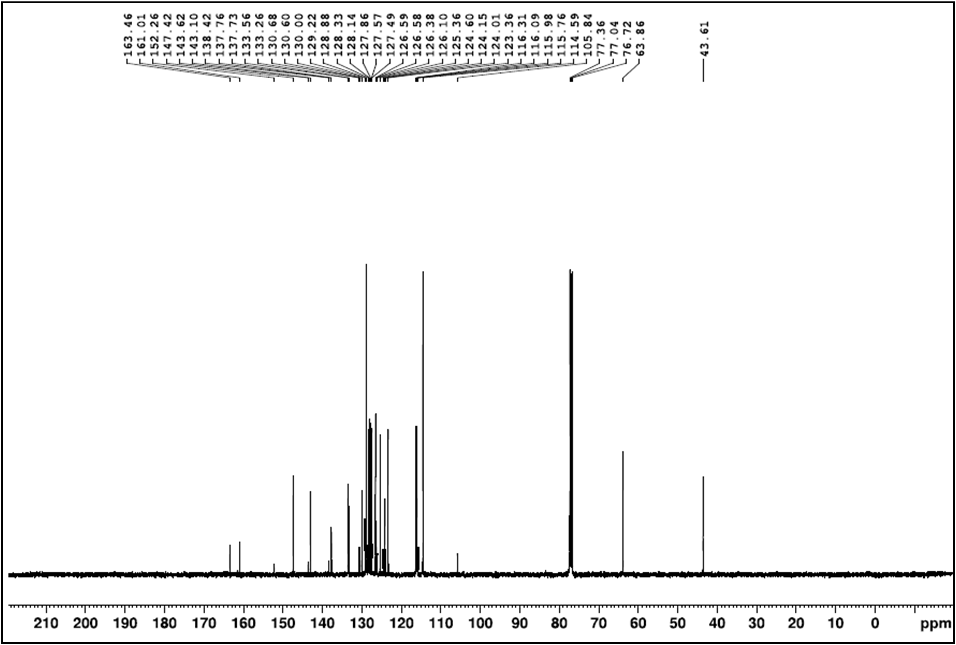


**Fig. S15.** ^13^C-NMR spectrum of compound **18** in CDCl_3_.


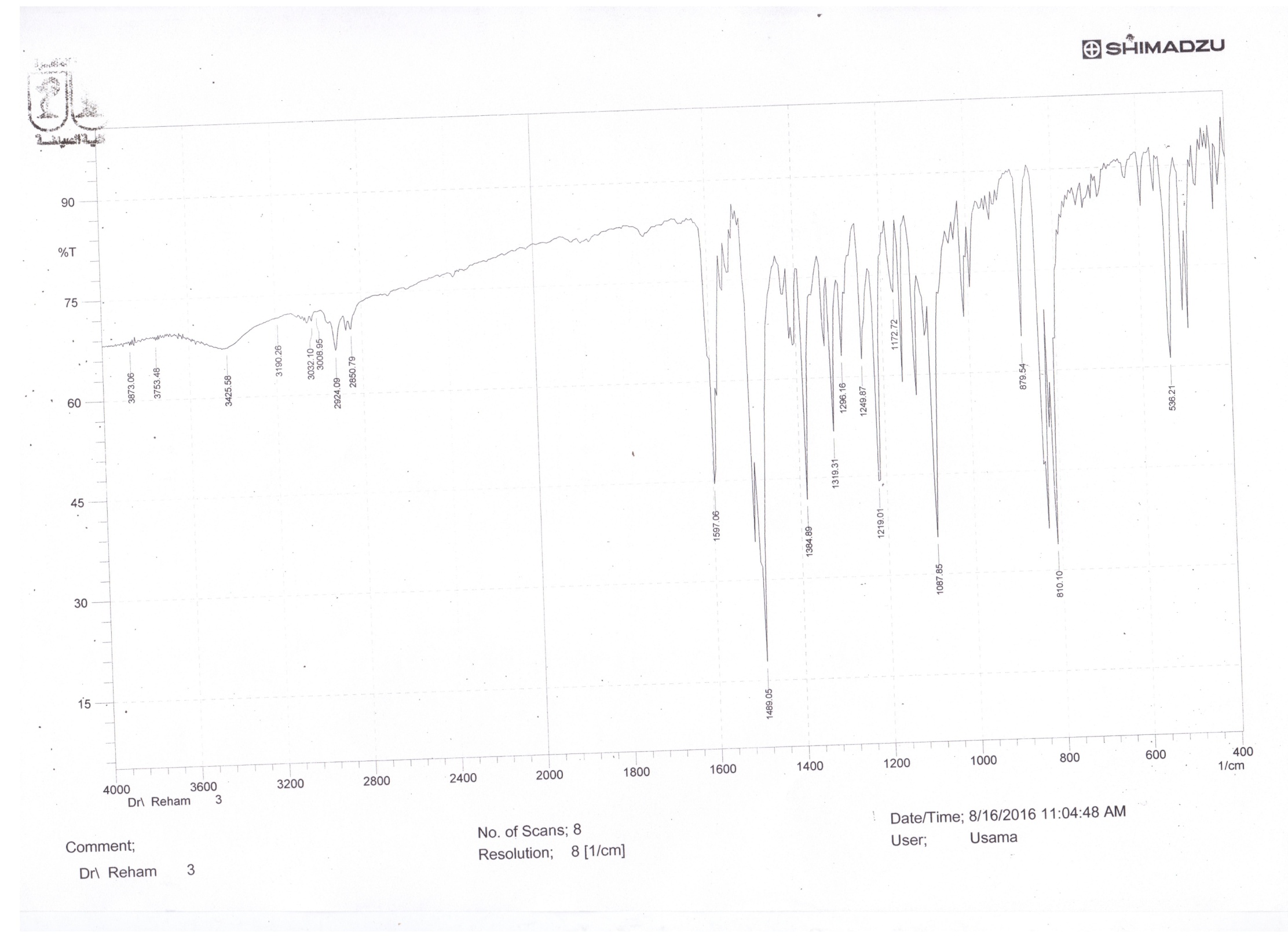


**Fig. S16.** IR spectrum of compound **19** (KBr pellet).


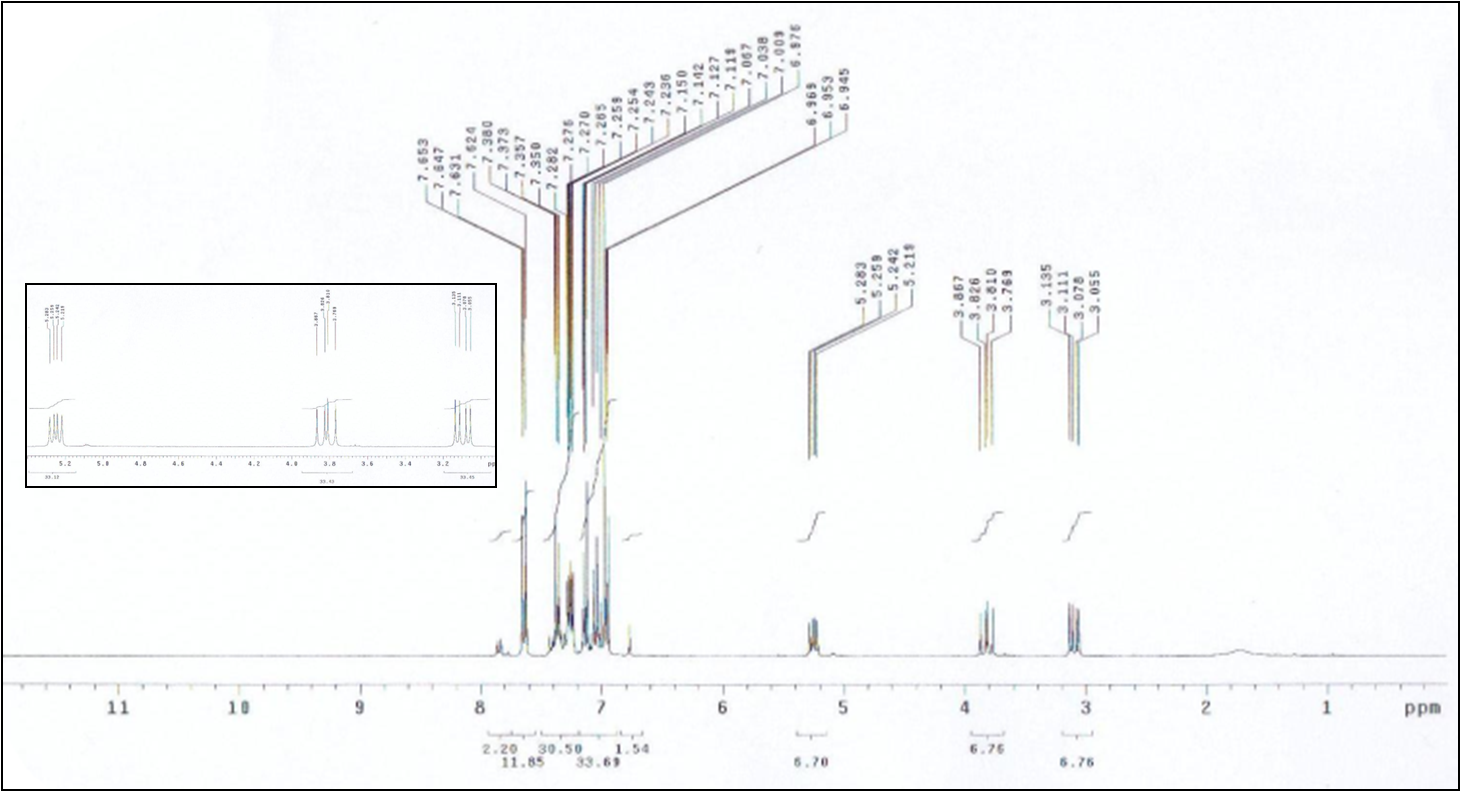


**Fig. S17.** ^1^H-NMR spectrum of compound **19** in CDCl_3_.


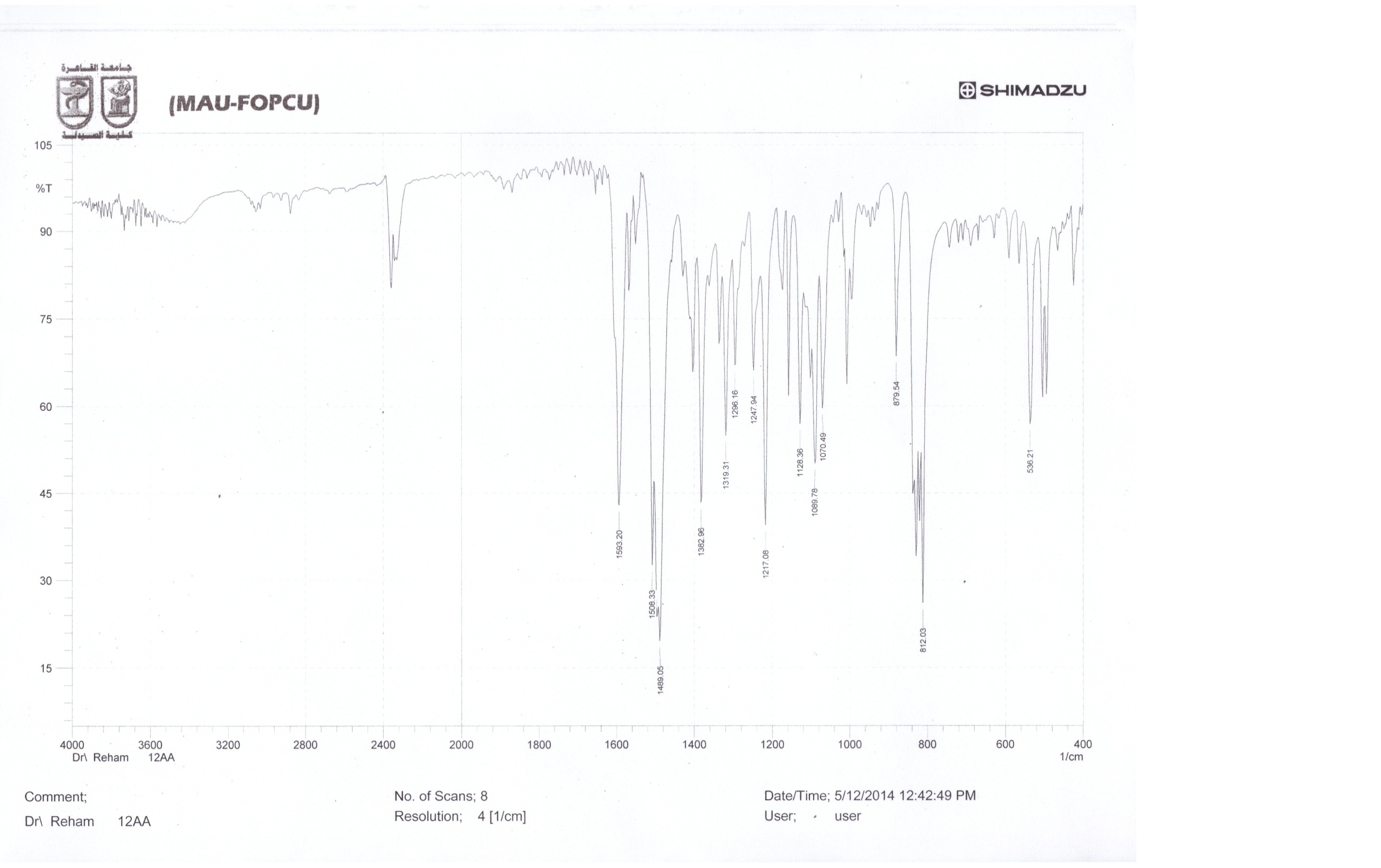


**Fig. S18.** IR spectrum of compound **20** (KBr pellet).


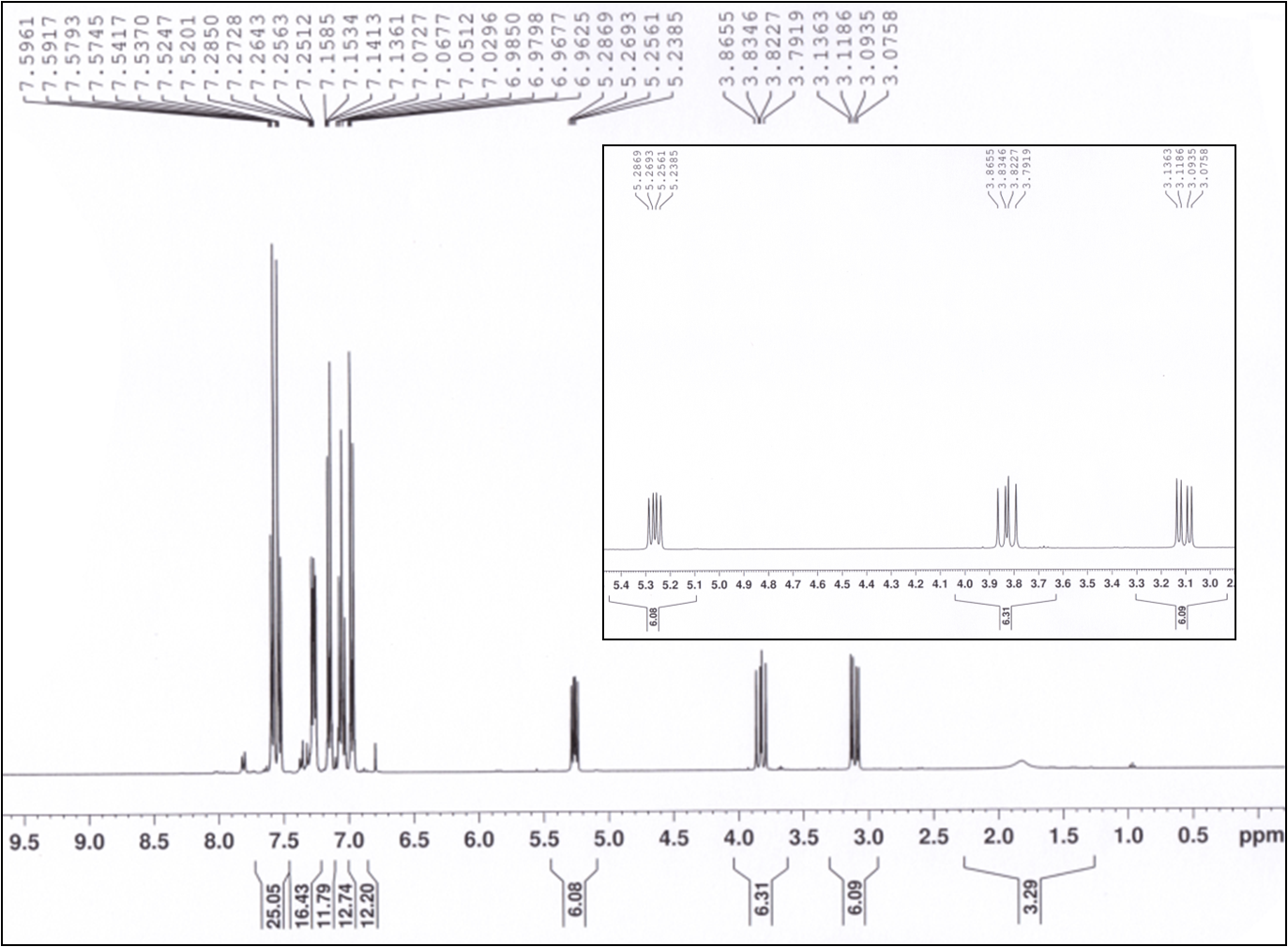


**Fig. S19.** ^1^H-NMR spectrum of compound **20** in CDCl_3_.


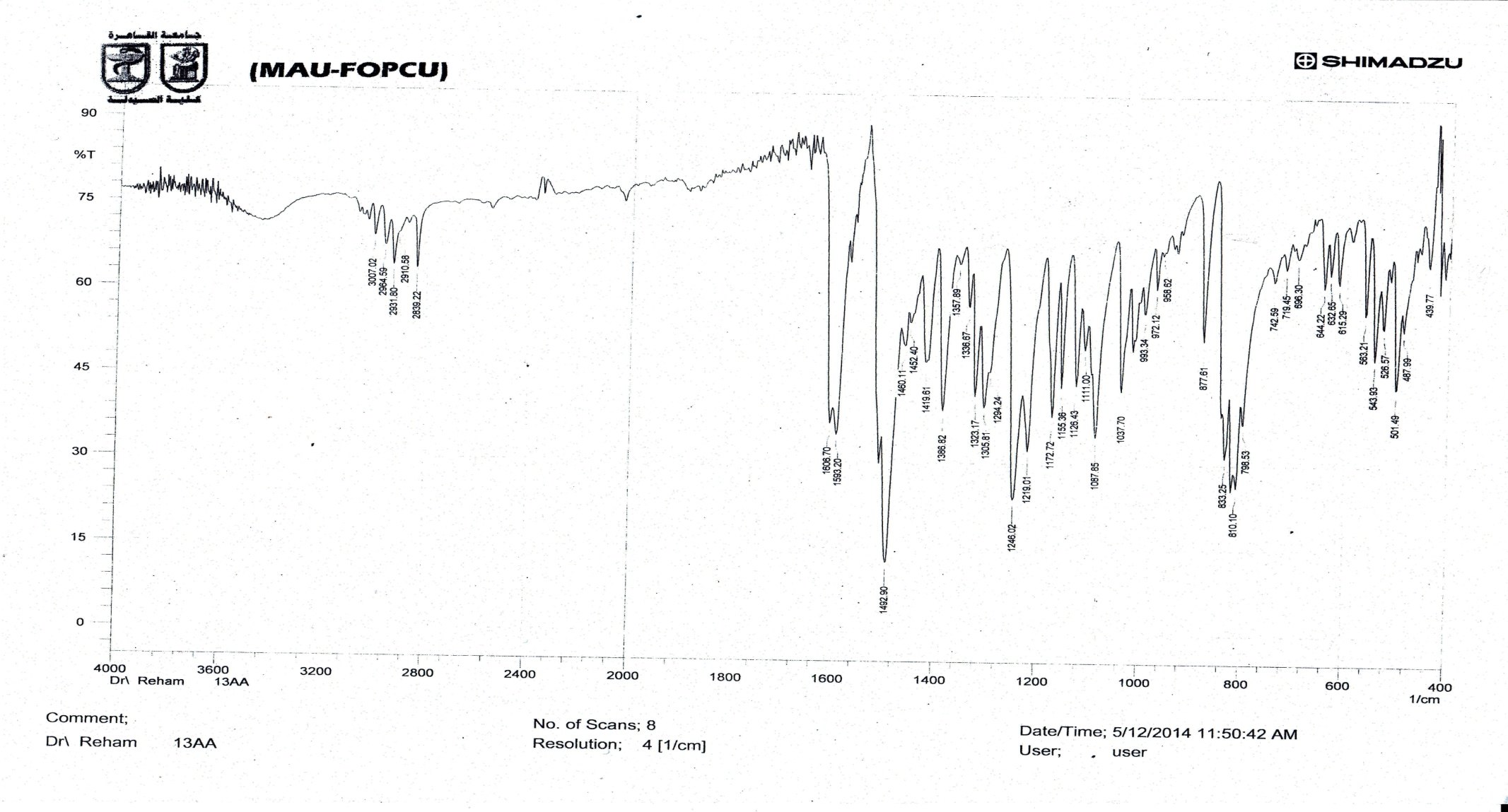


**Fig. S20.** IR spectrum of compound **21** (KBr pellet).


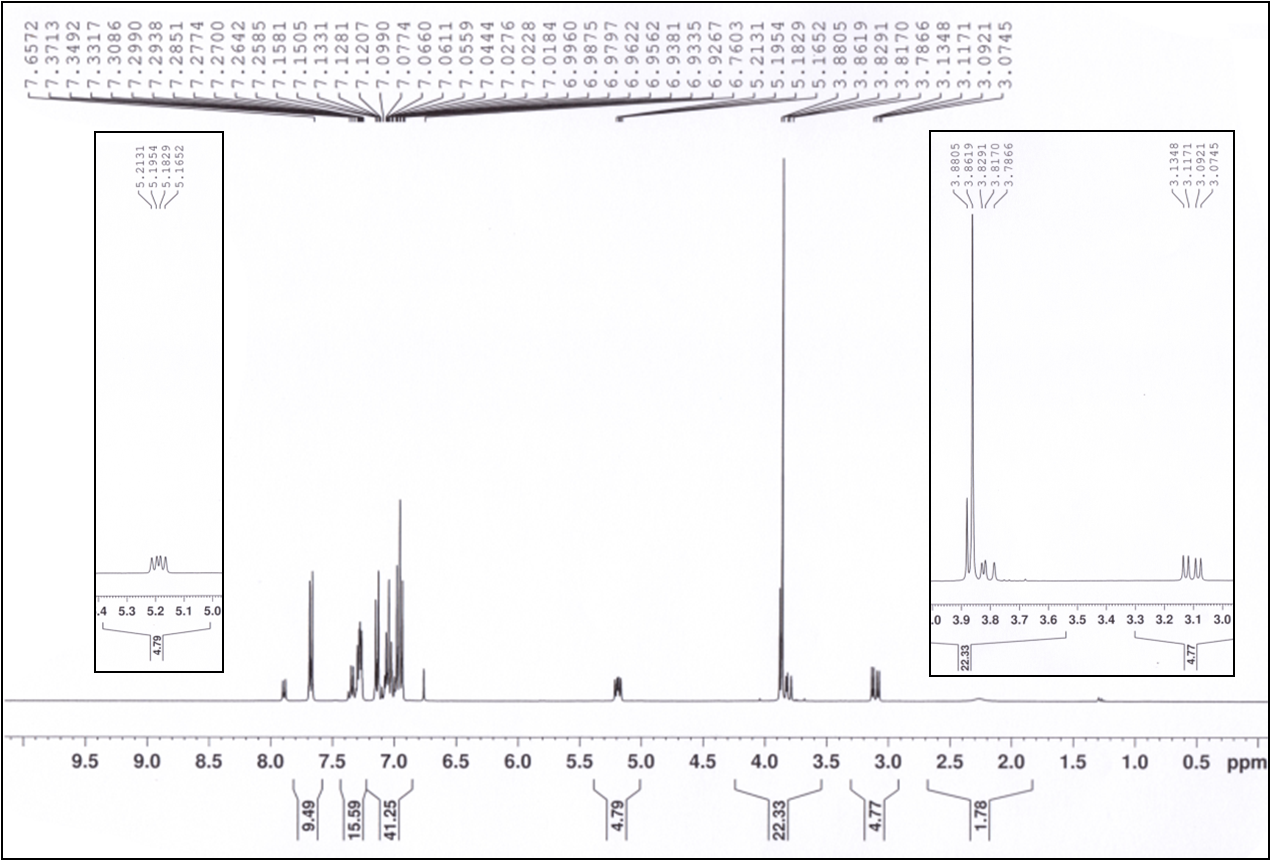


**Fig. S21.** ^1^H-NMR spectrum of compound **21** in CDCl_3_.


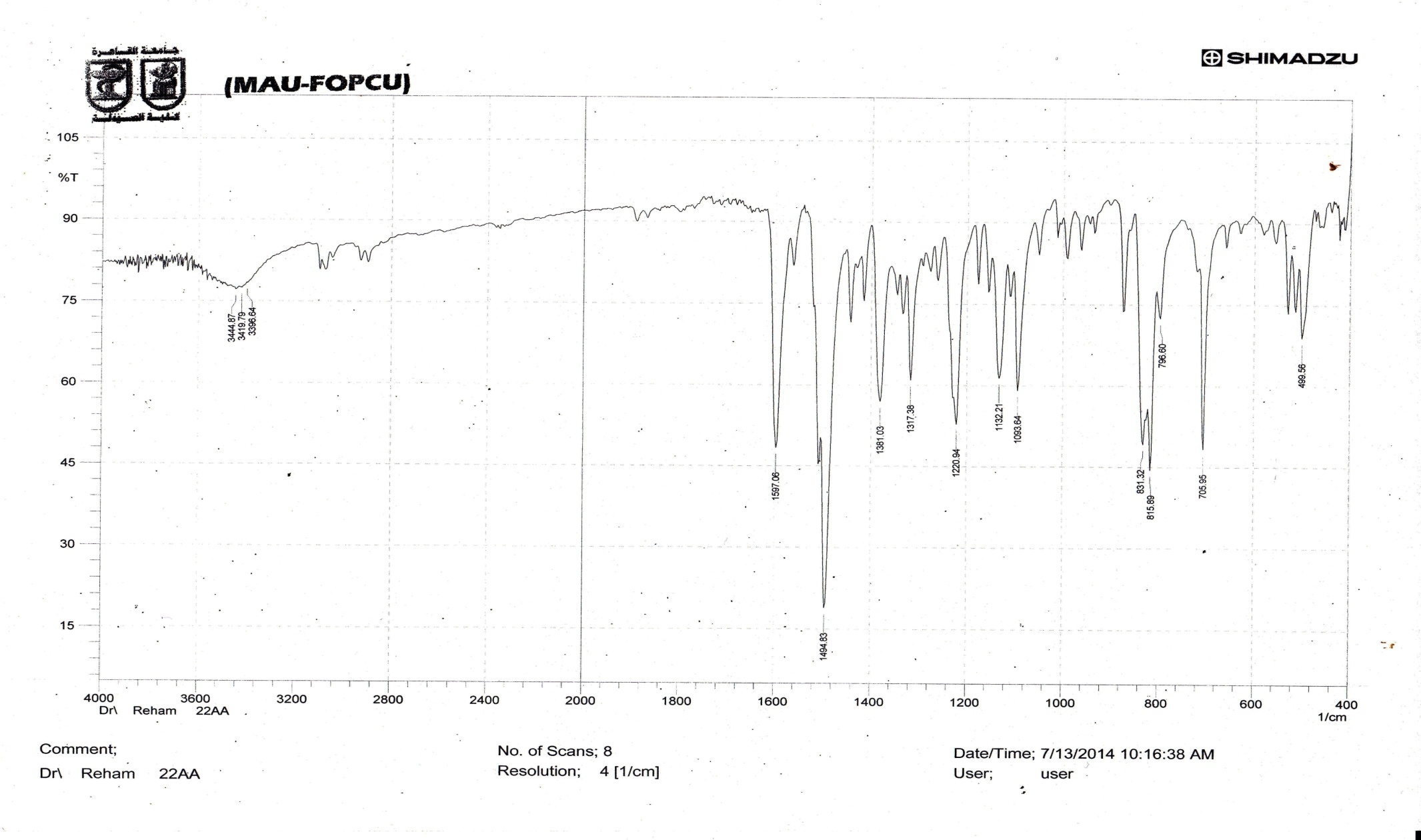


**Fig. S22.** IR spectrum of compound **22** (KBr pellet).

**
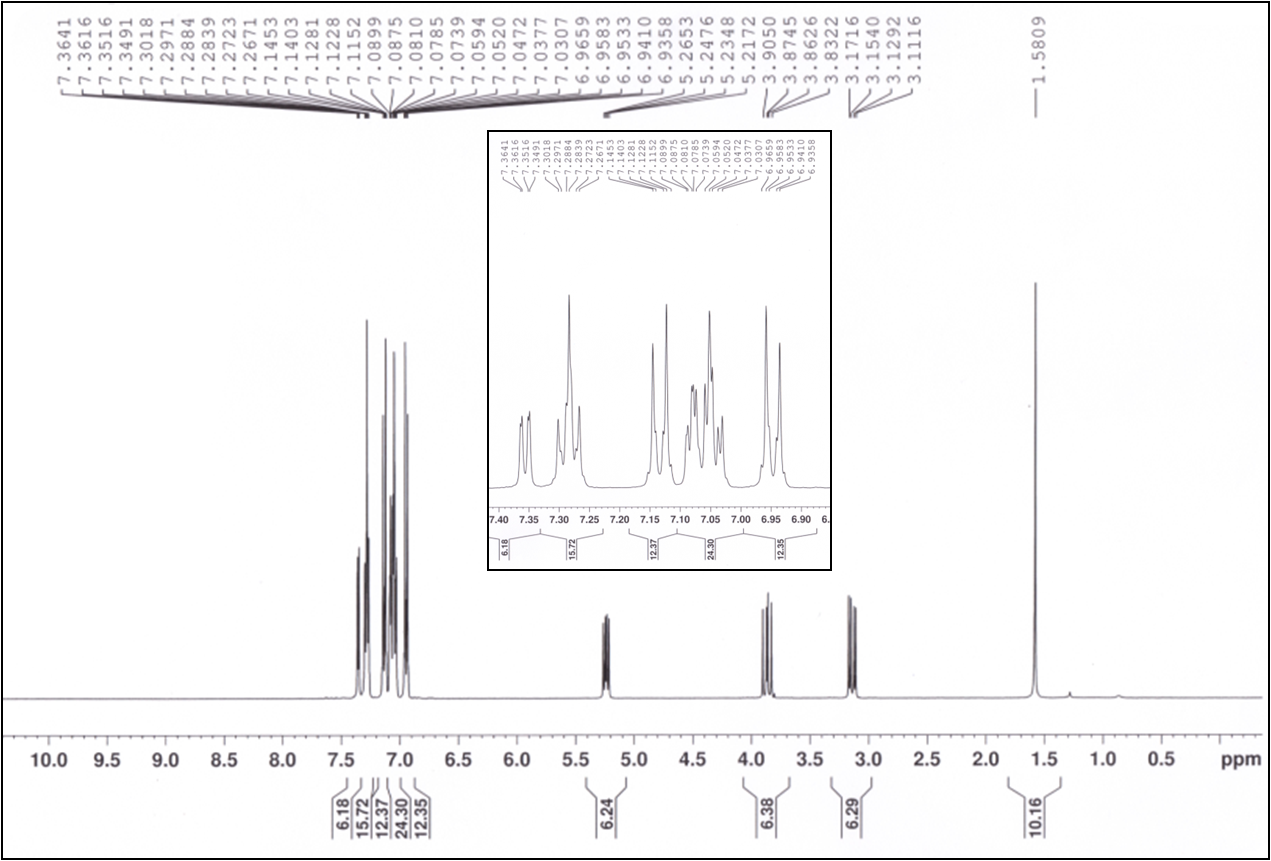
**

**Fig. S23.** ^1^H-NMR spectrum of compound **22** in CDCl_3_.


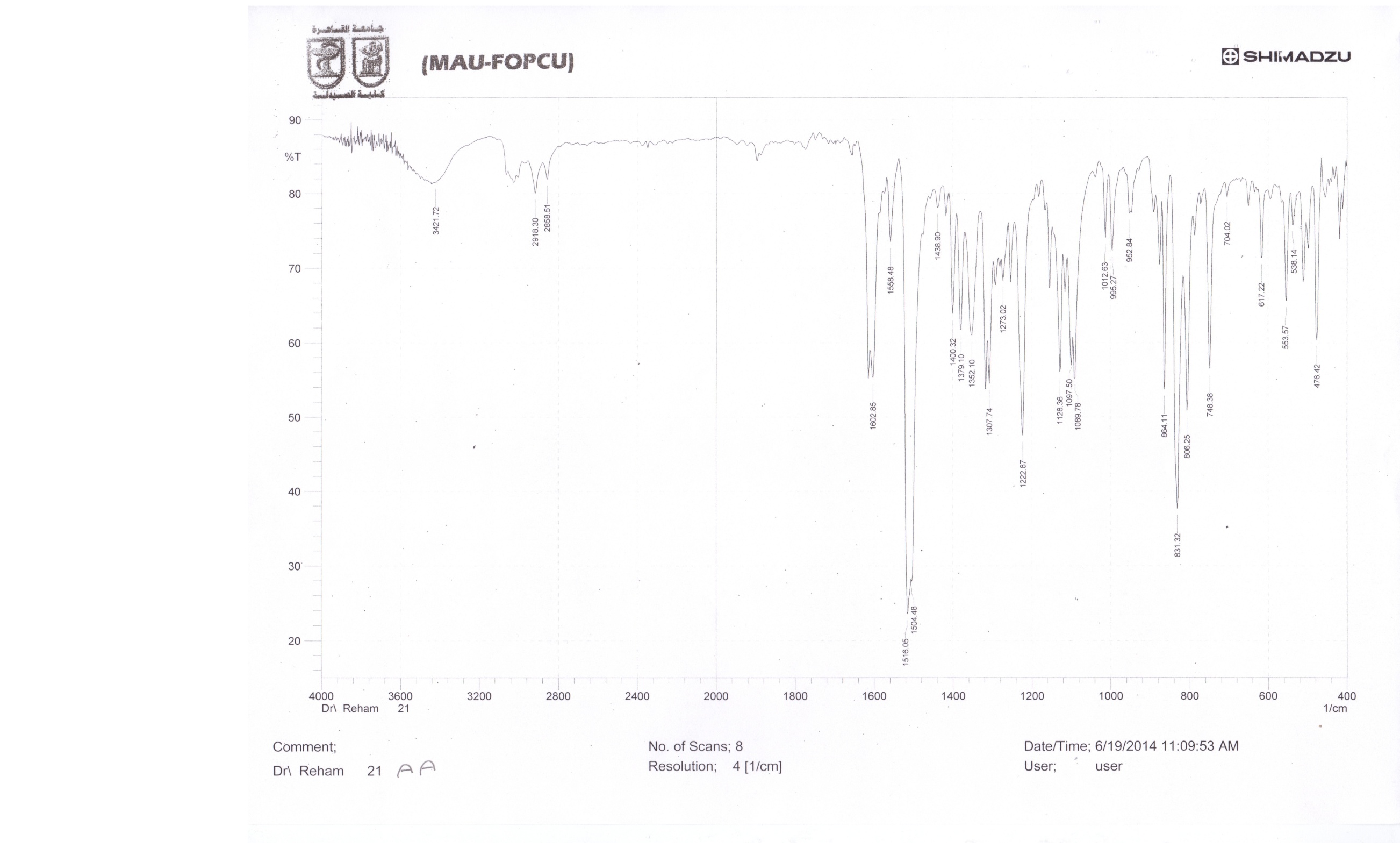


**Fig. S24.** IR spectrum of compound **23** (KBr pellet).


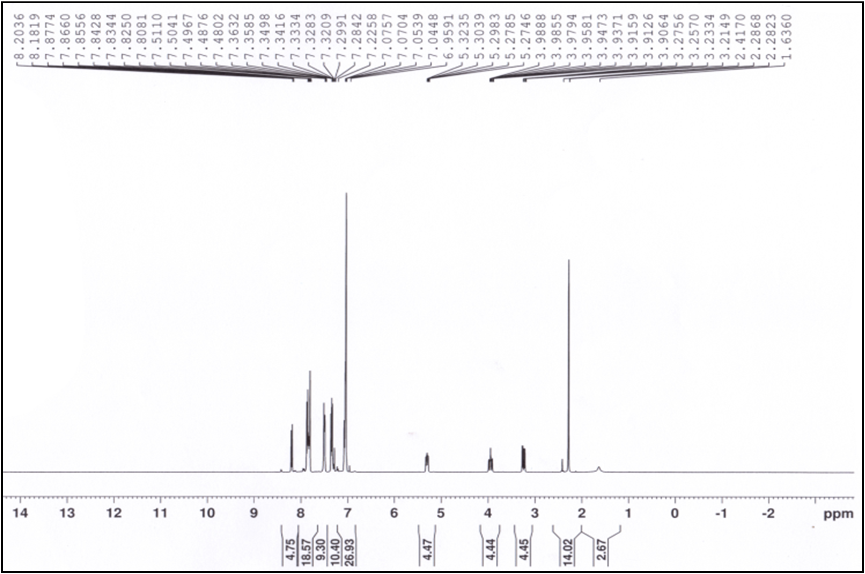


**Fig. S25.** ^1^H-NMR spectrum of compound **23** in CDCl_3_.


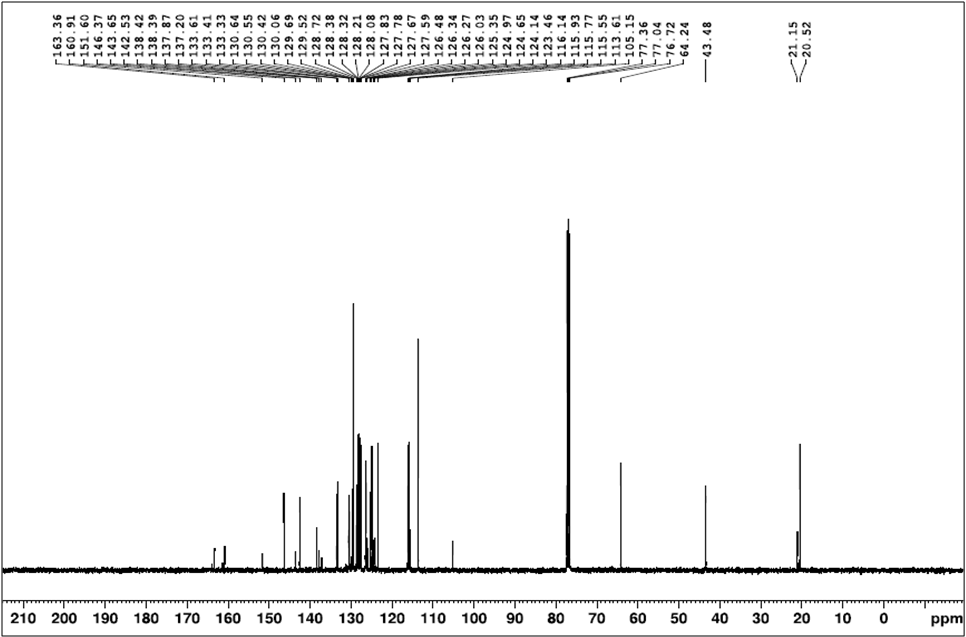


**Fig. S26.** ^13^C-NMR spectrum of compound **23** in CDCl_3_.


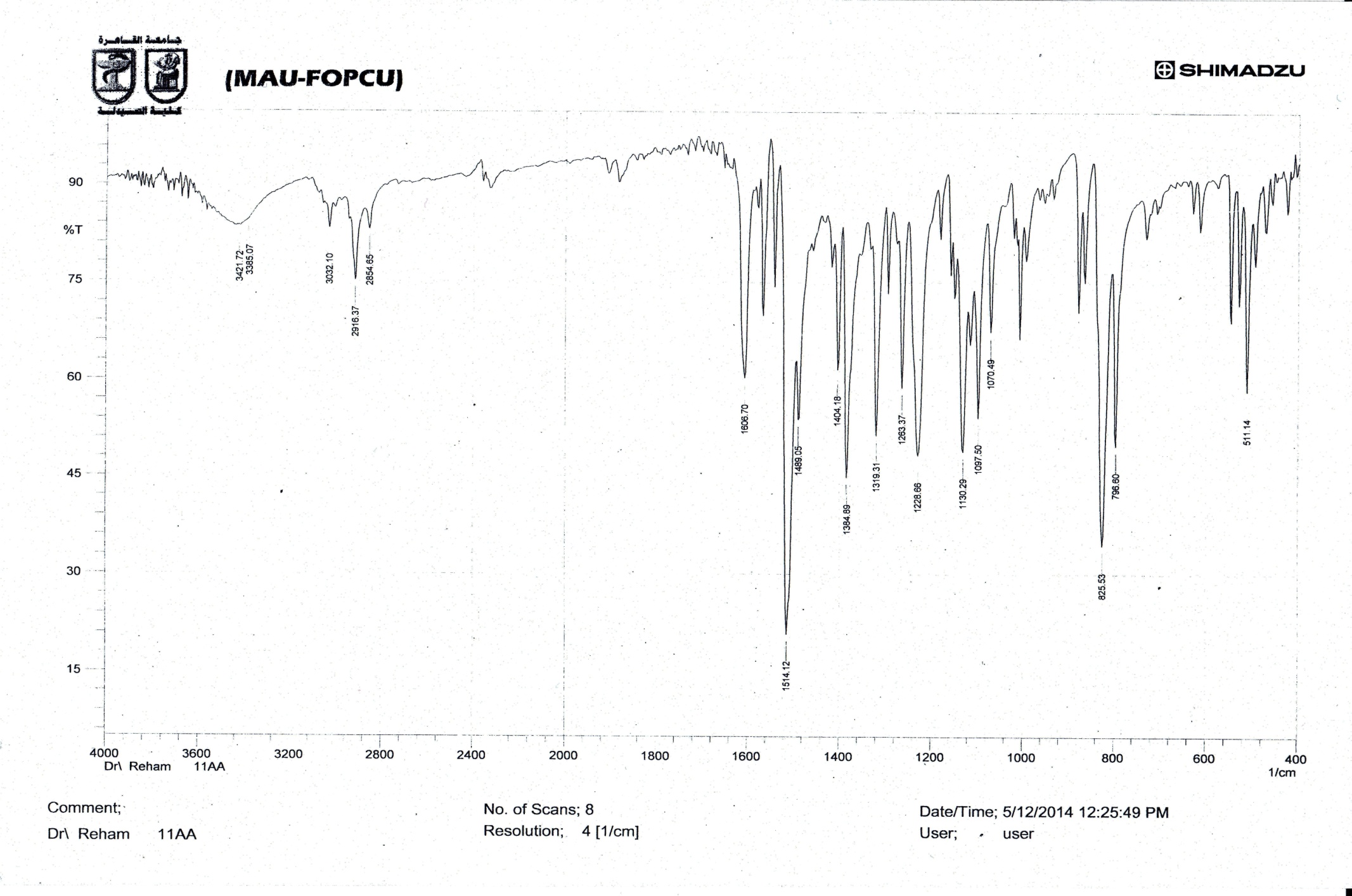


**Fig. S27.** IR spectrum of compound **24** (KBr pellet).


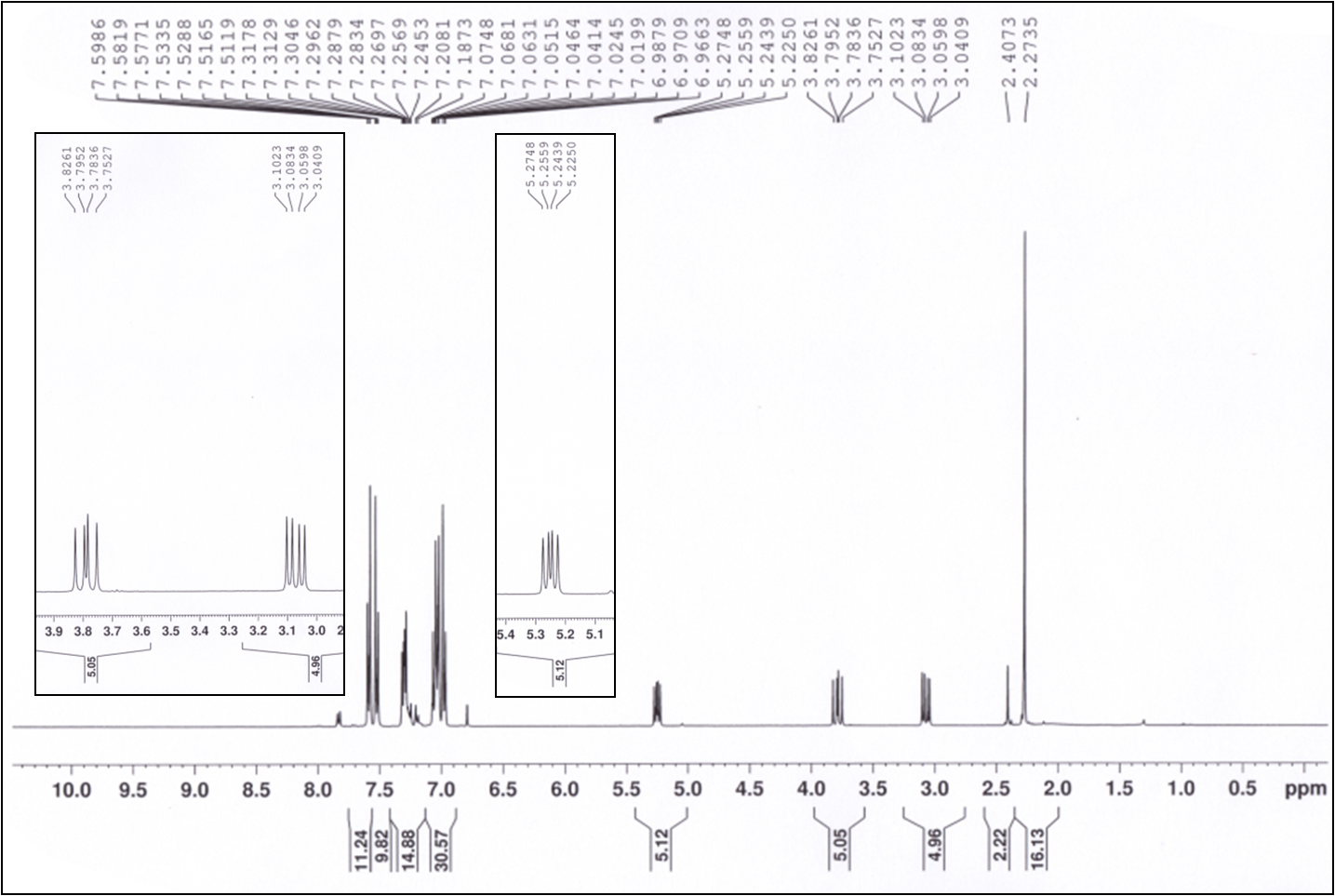


**Fig. S28.** ^1^H-NMR spectrum of compound **24** in CDCl_3_.

**
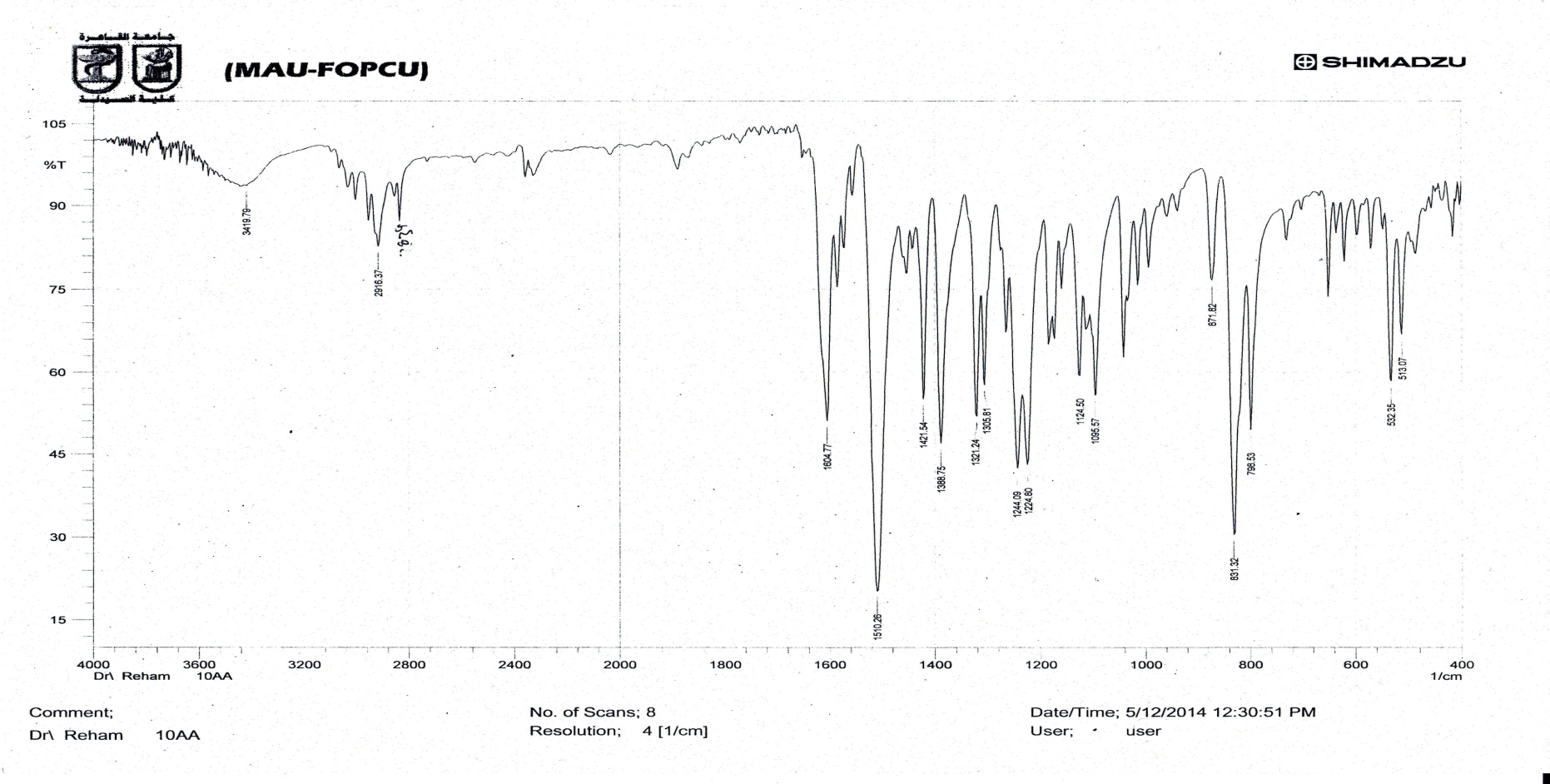
**

**Fig. S29.** IR spectrum of compound **25** (KBr pellet).

**
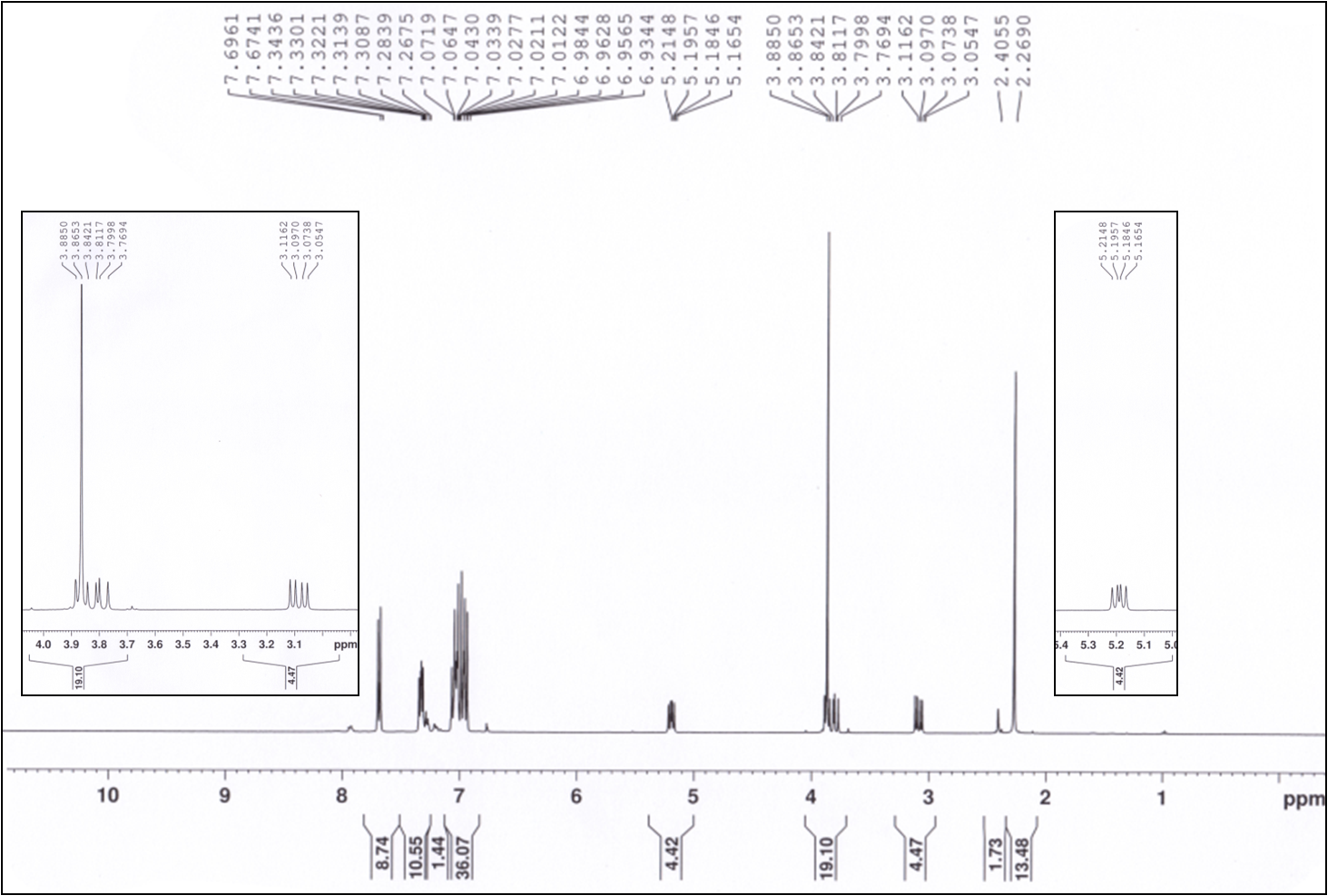
**

**Fig. S30.** ^1^H-NMR spectrum of compound **25** in CDCl_3_.**Fig. S31.** IR spectrum of compound **26** (KBr pellet).


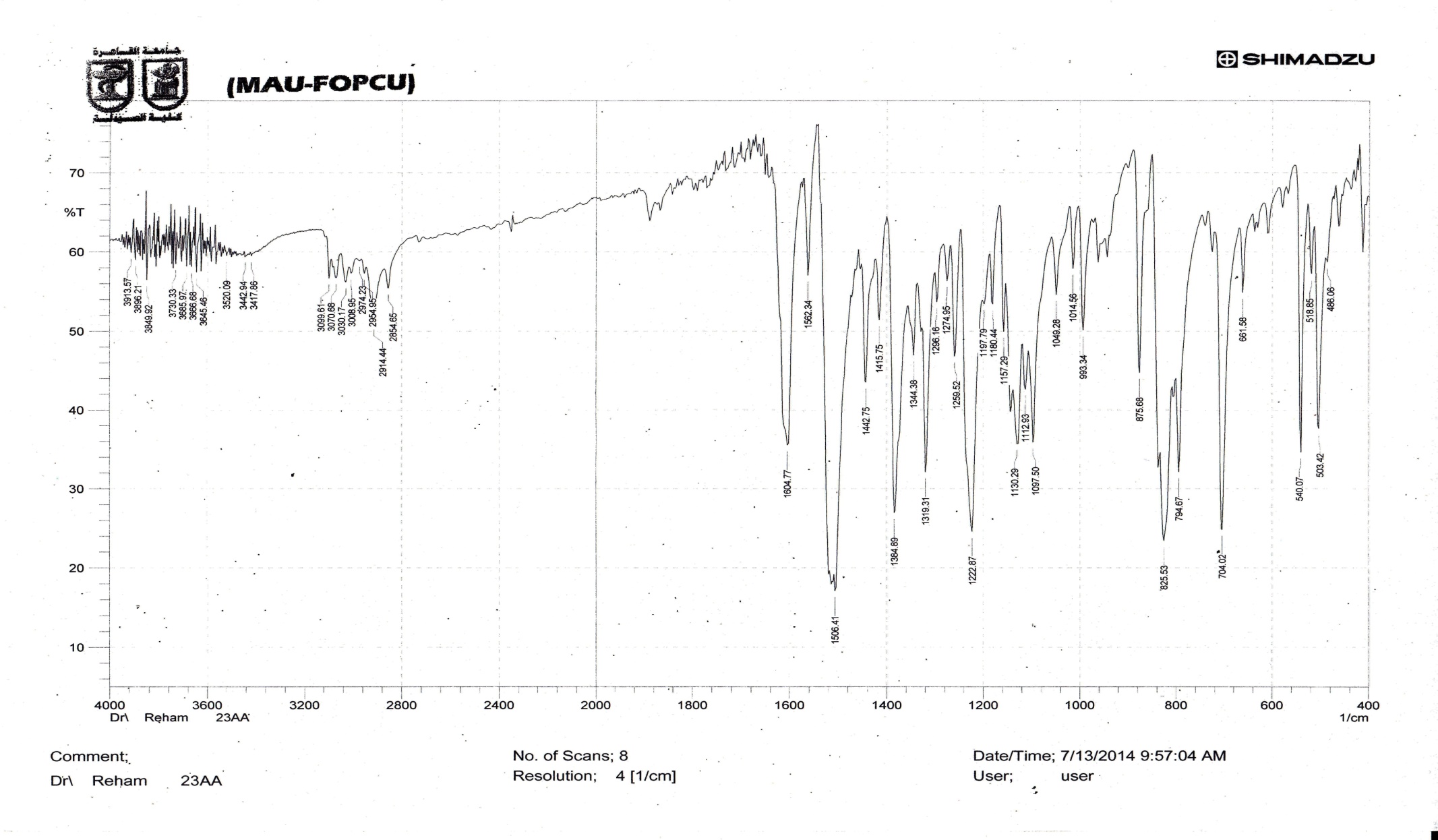


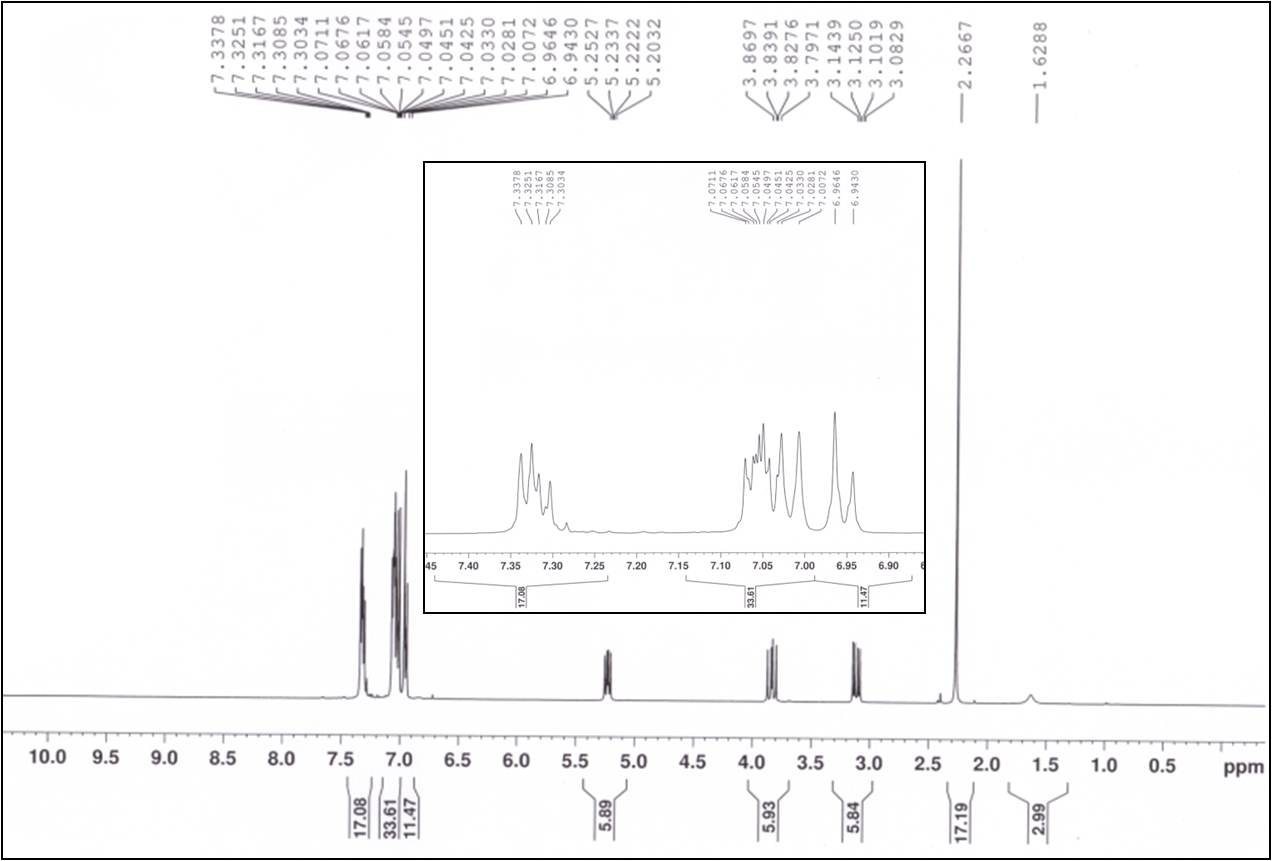


**Fig. S32.** ^1^H-NMR spectrum of compound **26** in CDCl_3_.


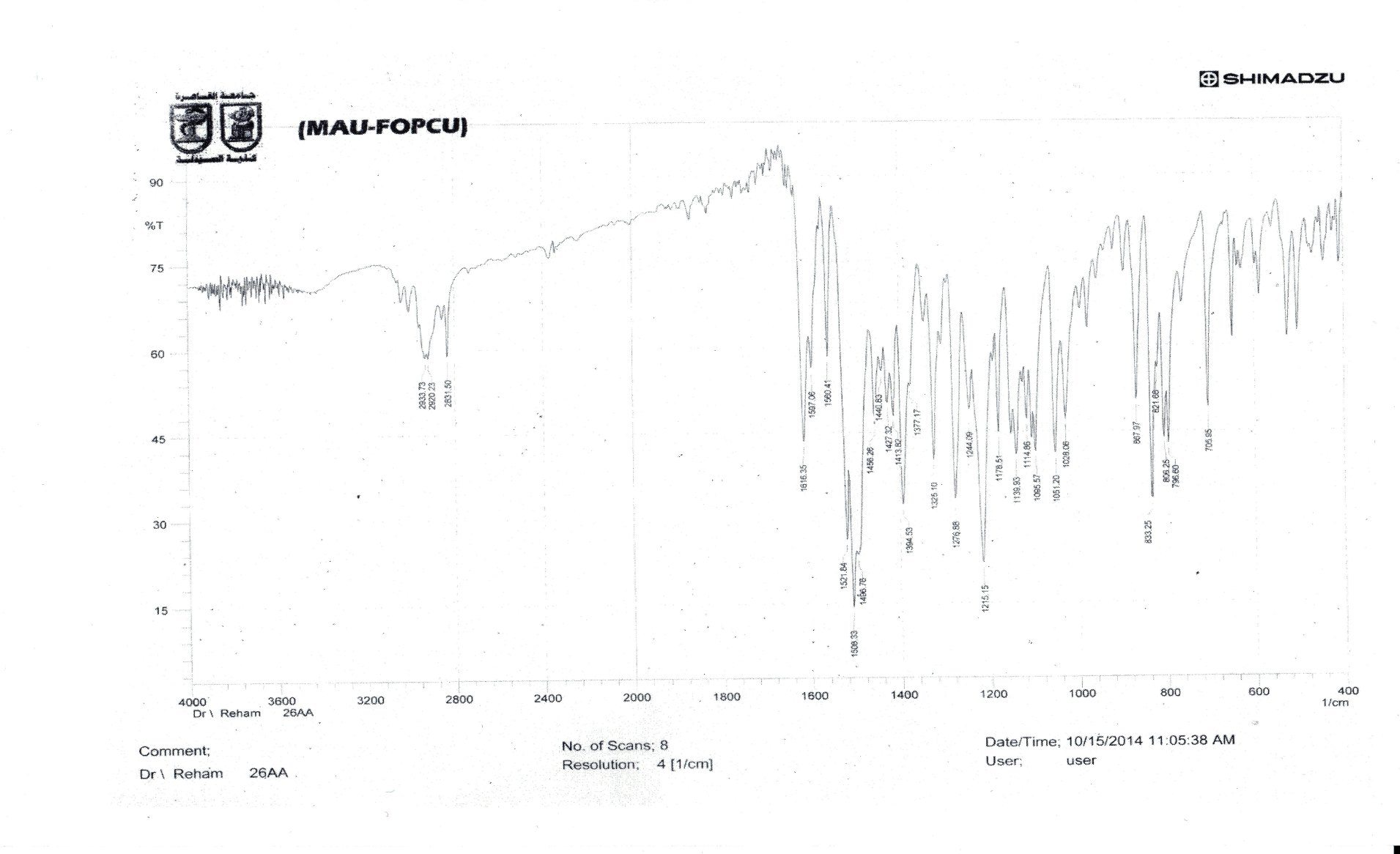


**Fig. S33.** IR spectrum of compound **27** (KBr pellet).

**
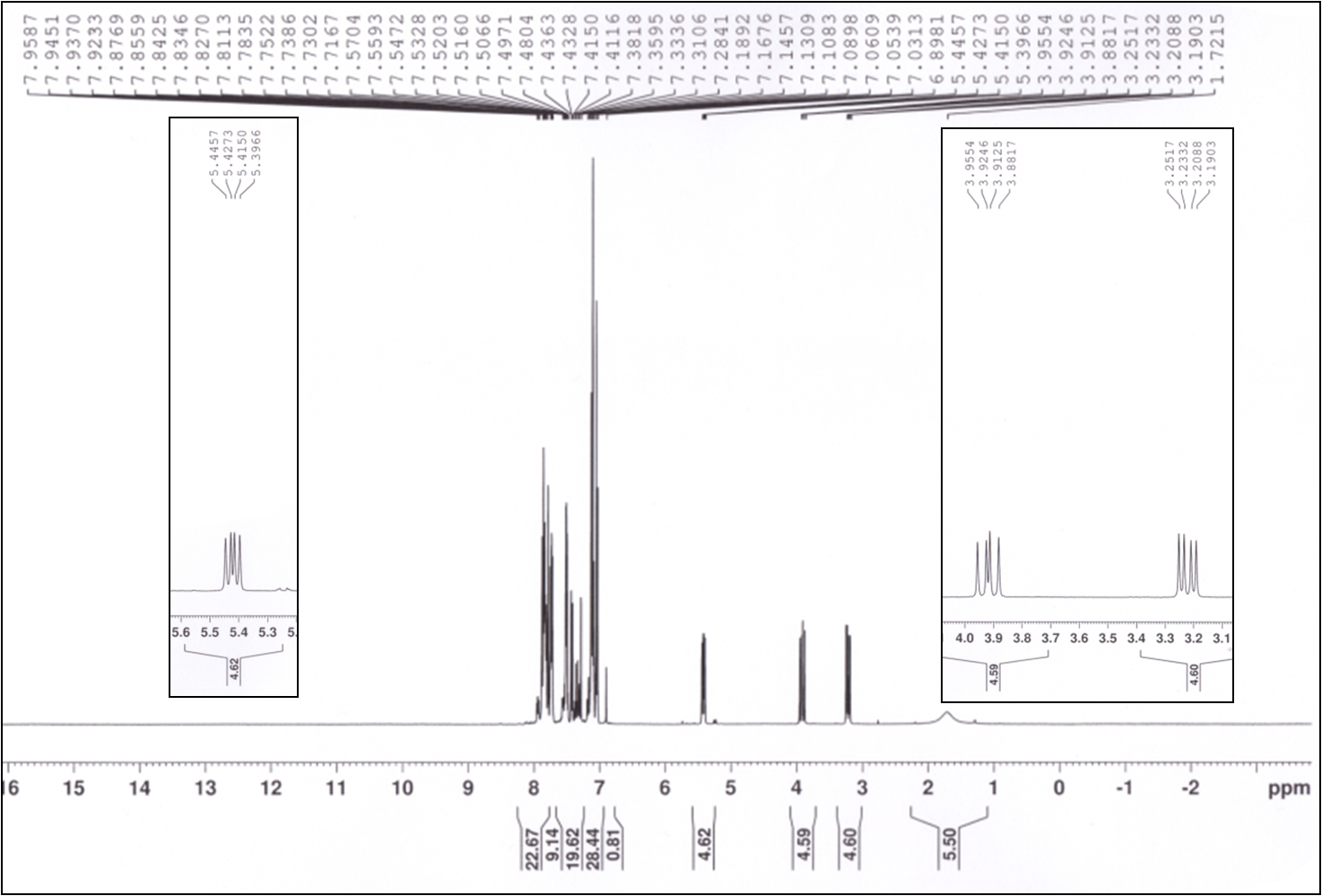
**

**Fig. S34.** ^1^H-NMR spectrum of compound **27** in CDCl_3_.


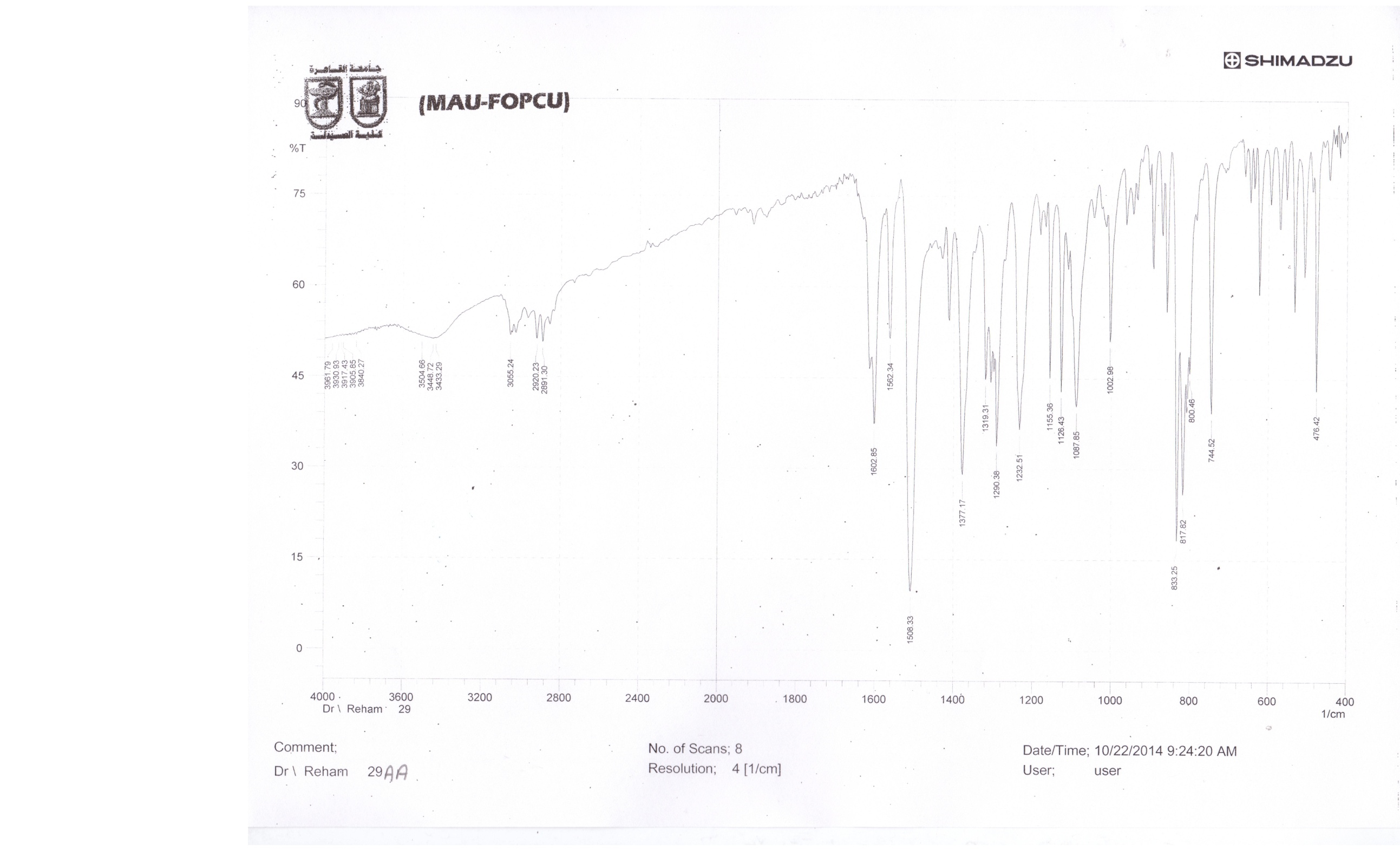


**Fig. S35.** IR spectrum of compound **28** (KBr pellet).


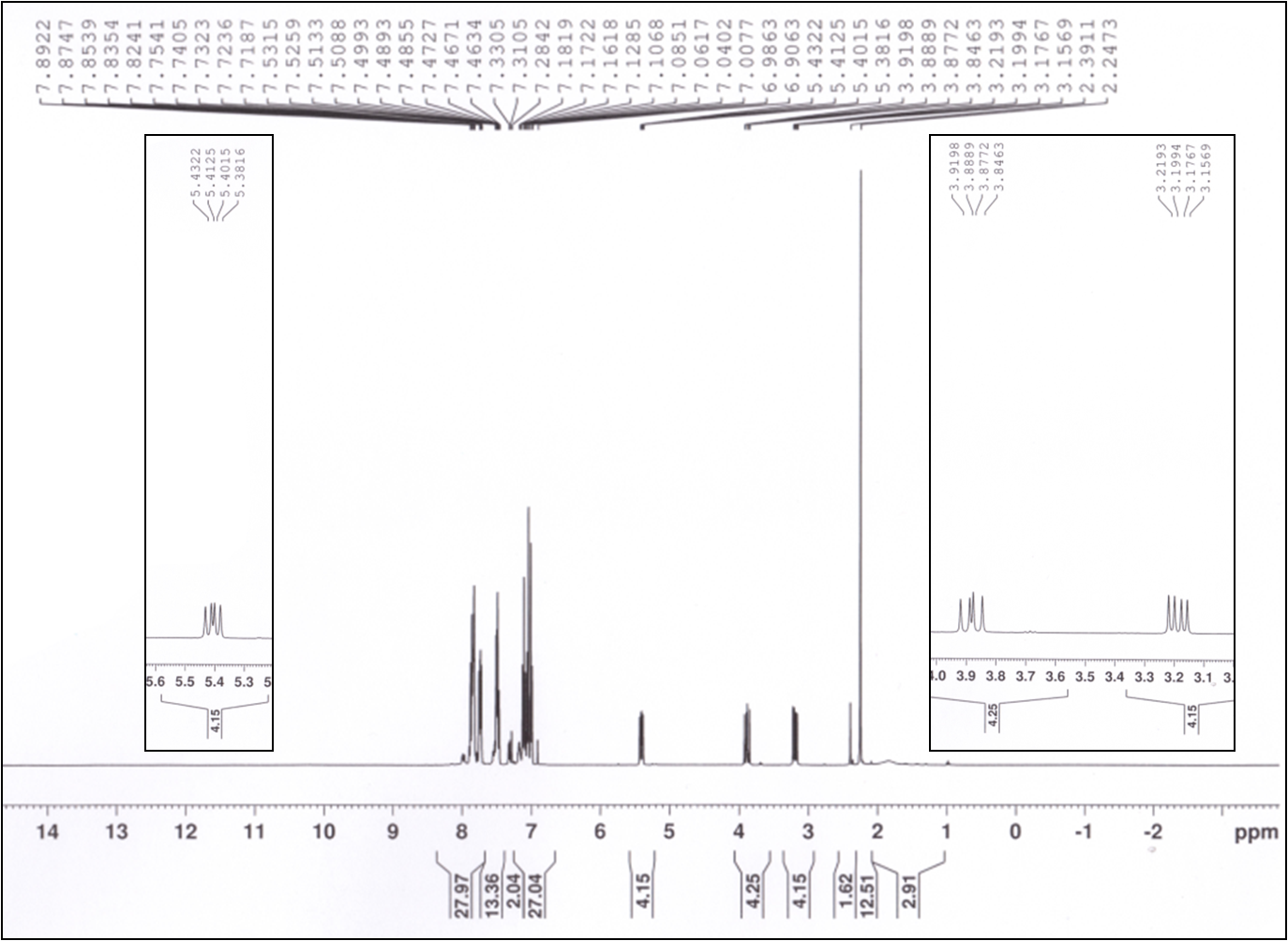


**Fig. S36.** ^1^H-NMR spectrum of compound **28** in CDCl_3_.


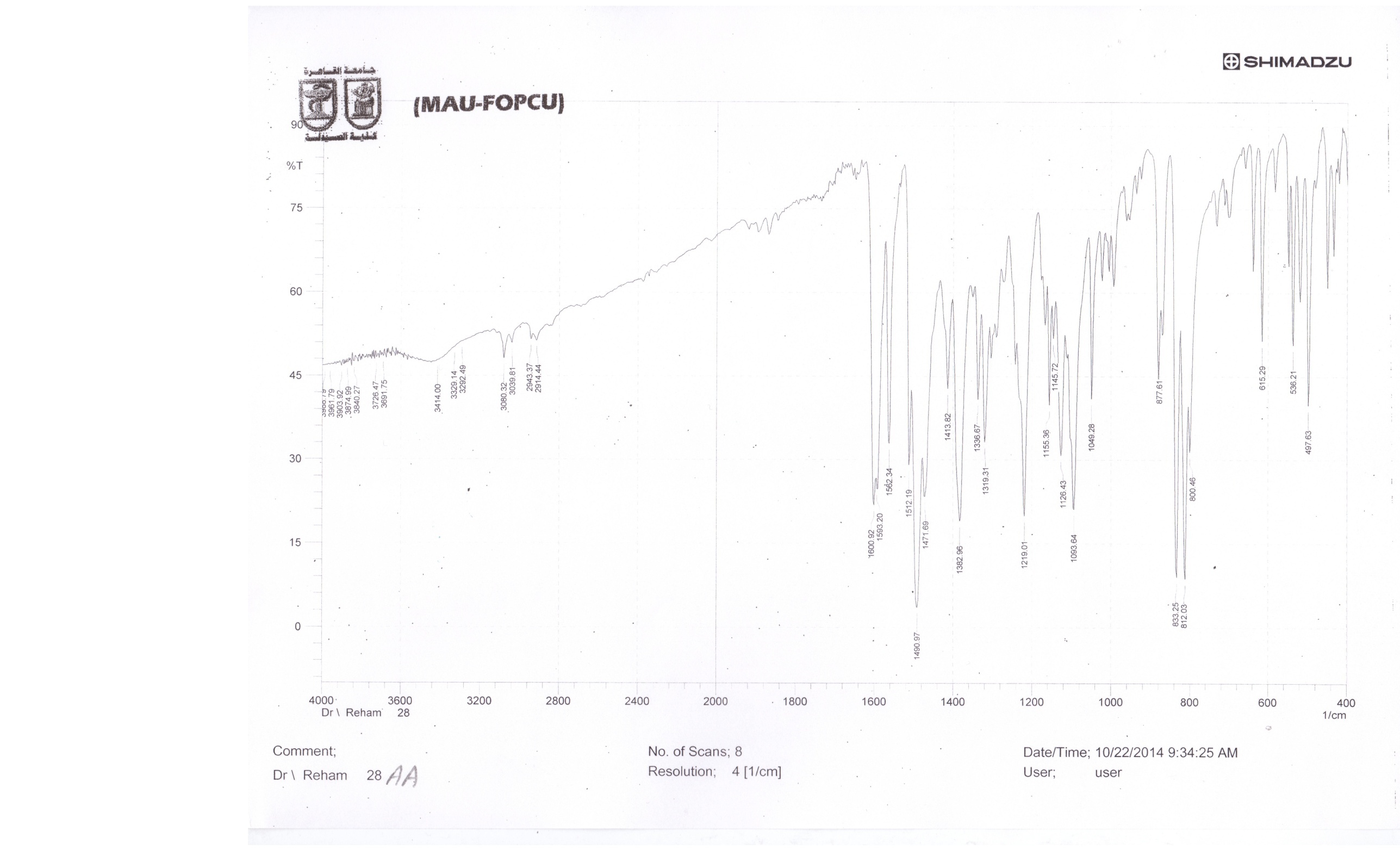


**Fig. S37.** IR spectrum of compound **29** (KBr pellet).

**
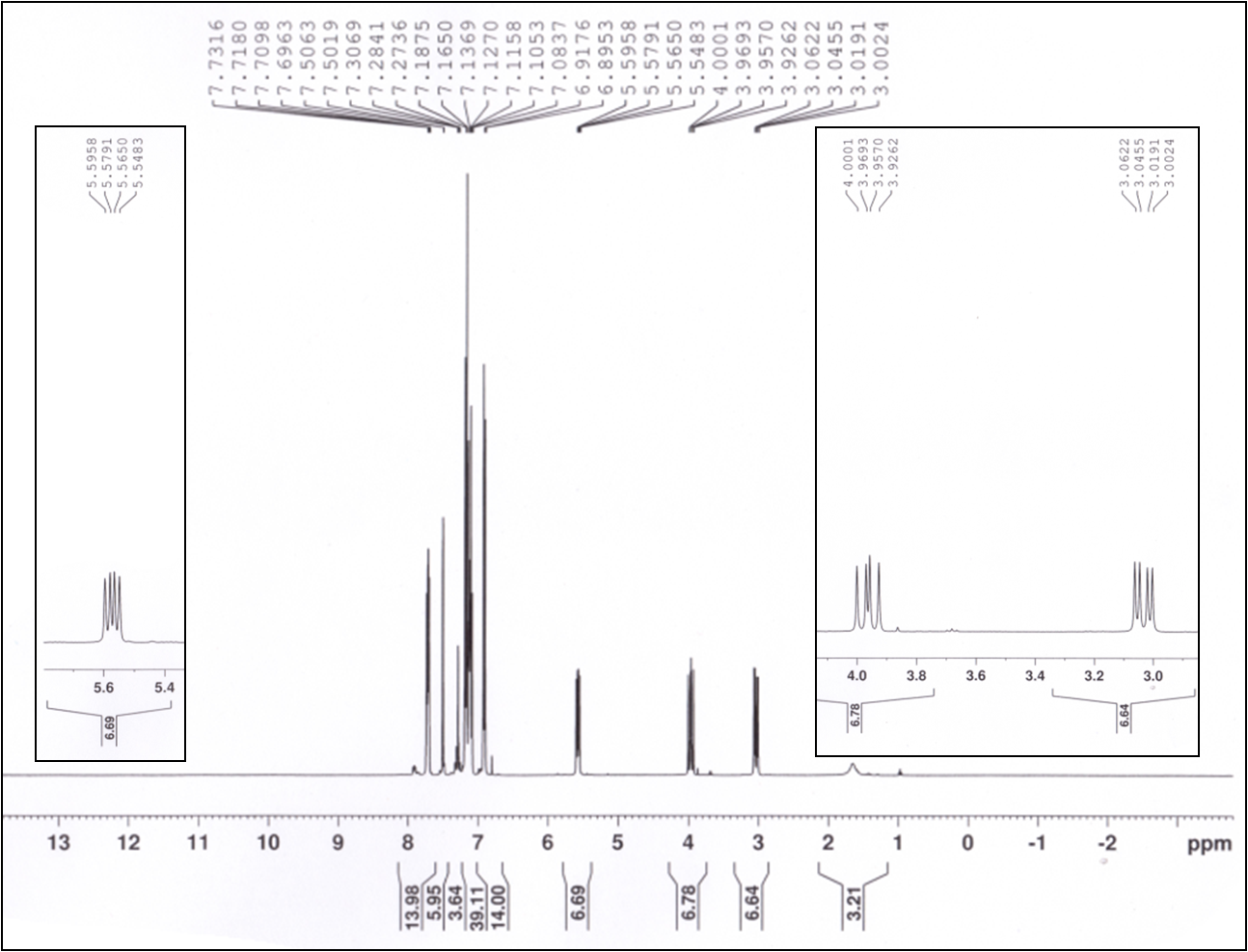
**

**Fig. S38.** ^1^H-NMR spectrum of compound **29** in CDCl_3_.


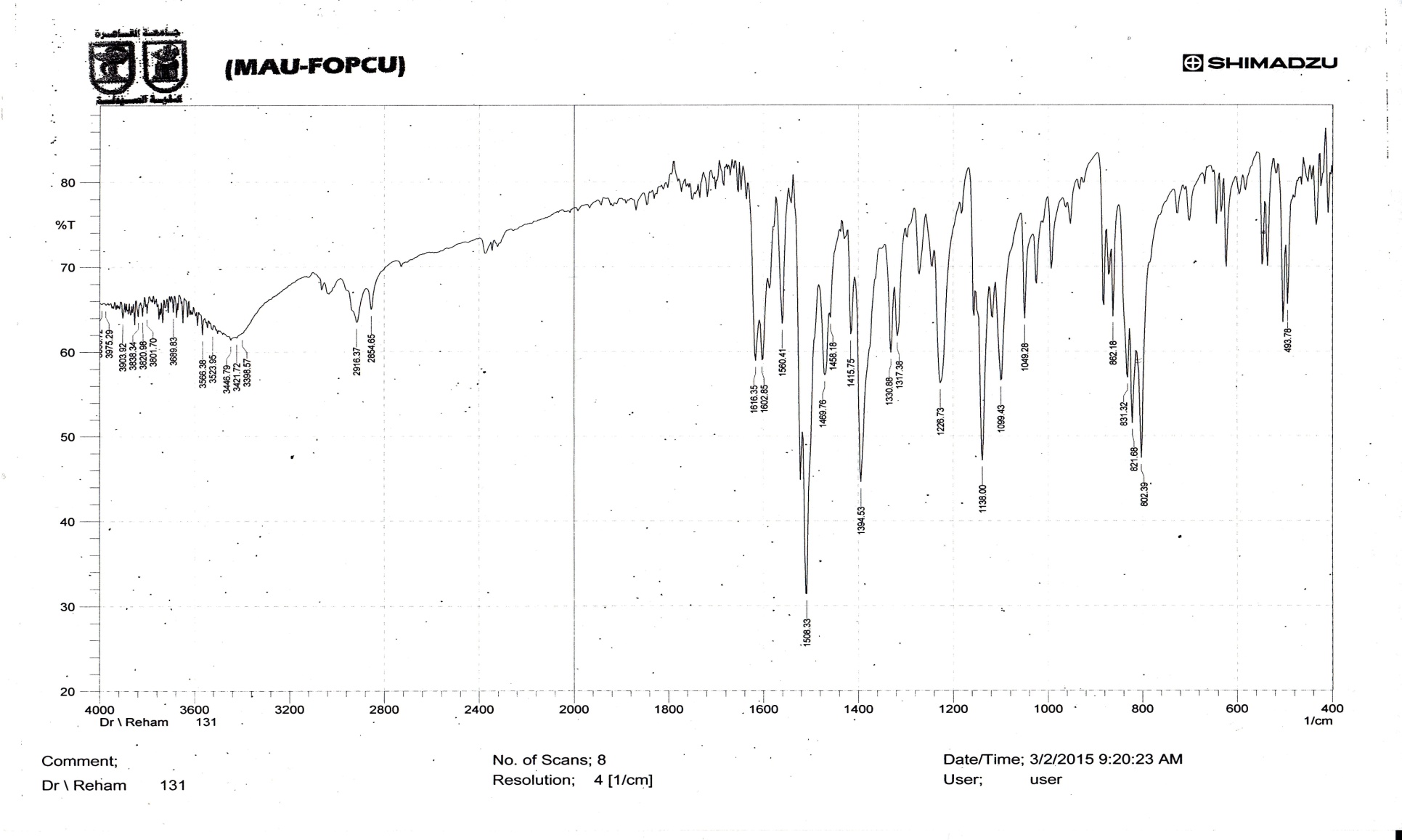


**Fig. S39.** IR spectrum of compound **30** (KBr pellet).

**
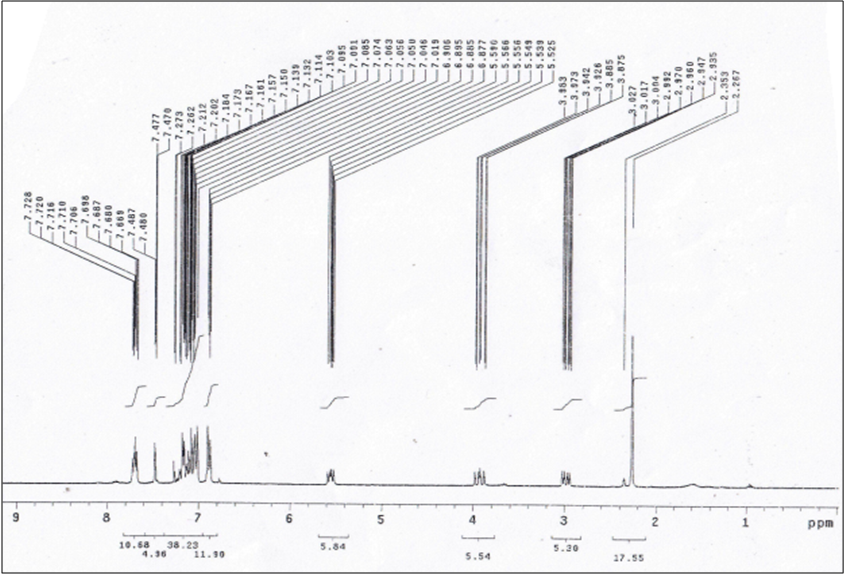
**

**Fig. S40.** ^1^H-NMR spectrum of compound **30** in CDCl_3_.


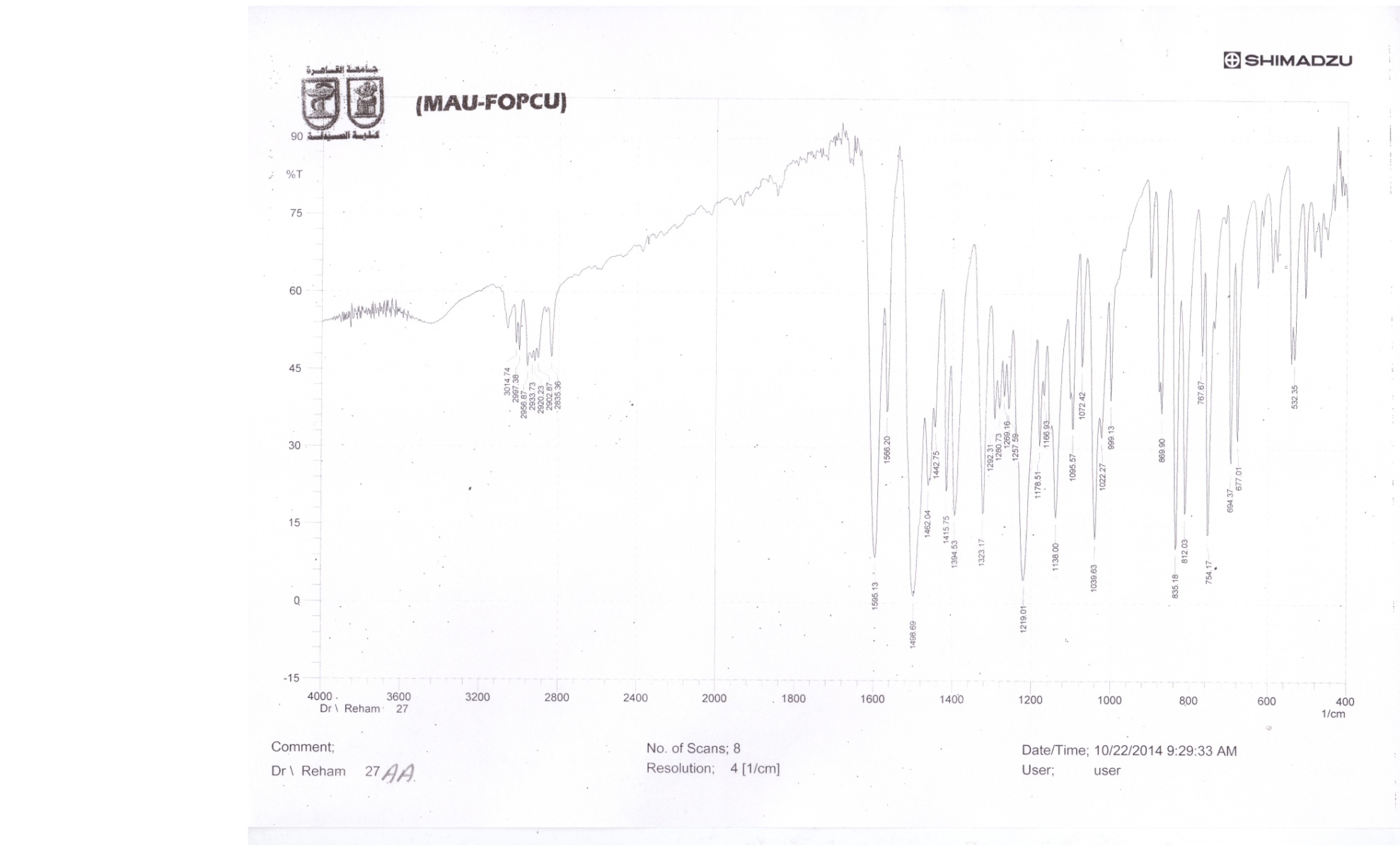


**Fig. S41.** IR spectrum of compound **31** (KBr pellet).


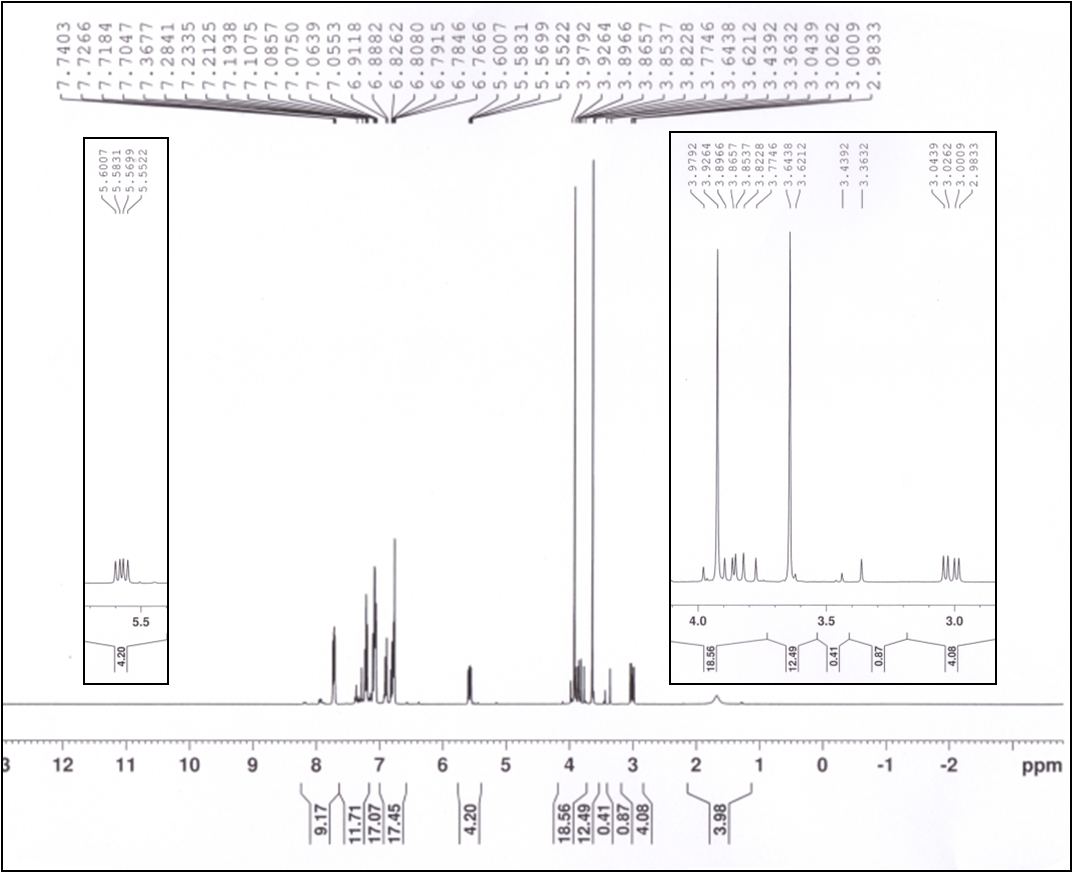


**Fig. S42.** ^1^H-NMR spectrum of compound **31** in CDCl_3_.


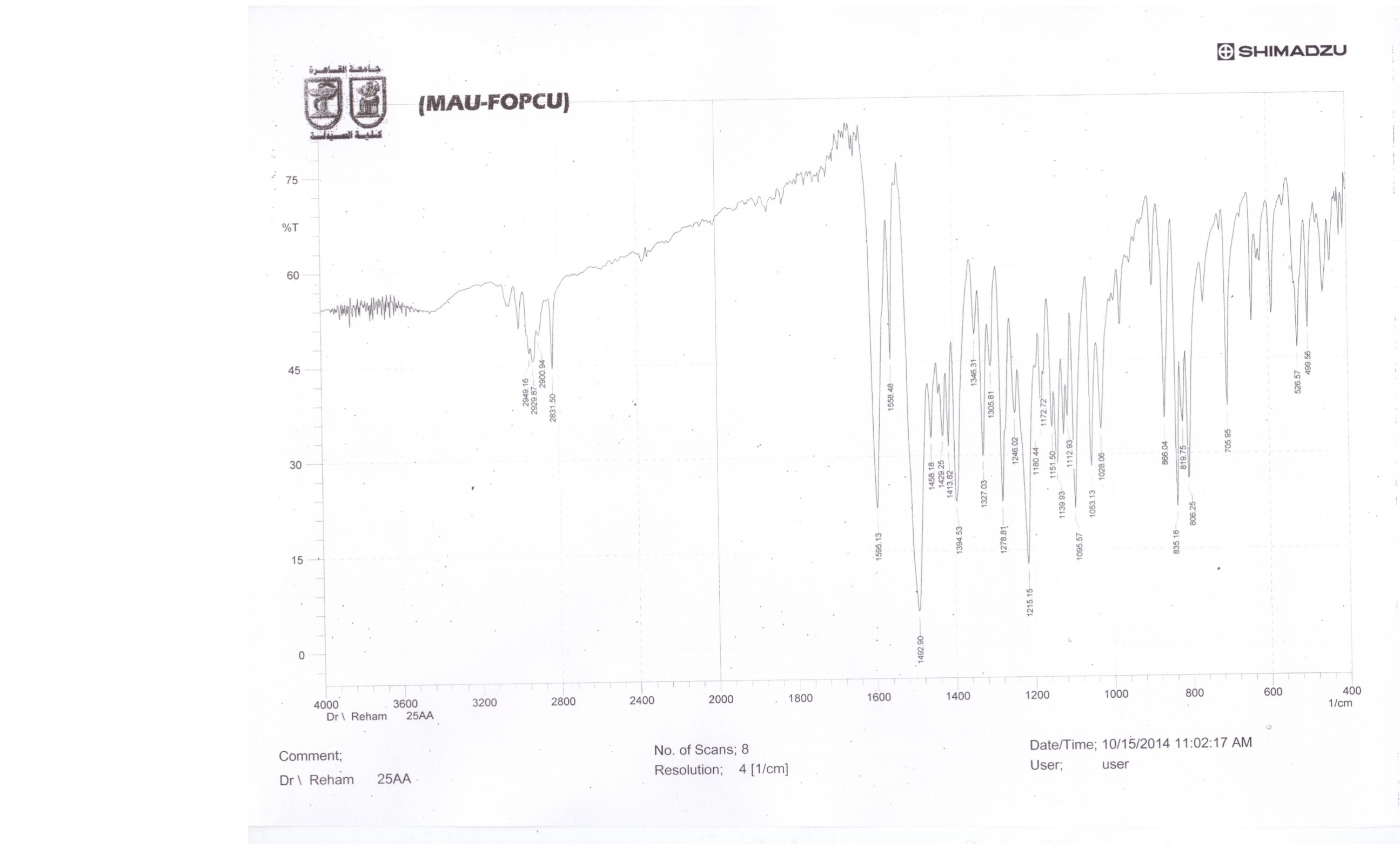


**Fig. S43.** IR spectrum of compound **32** (KBr pellet).


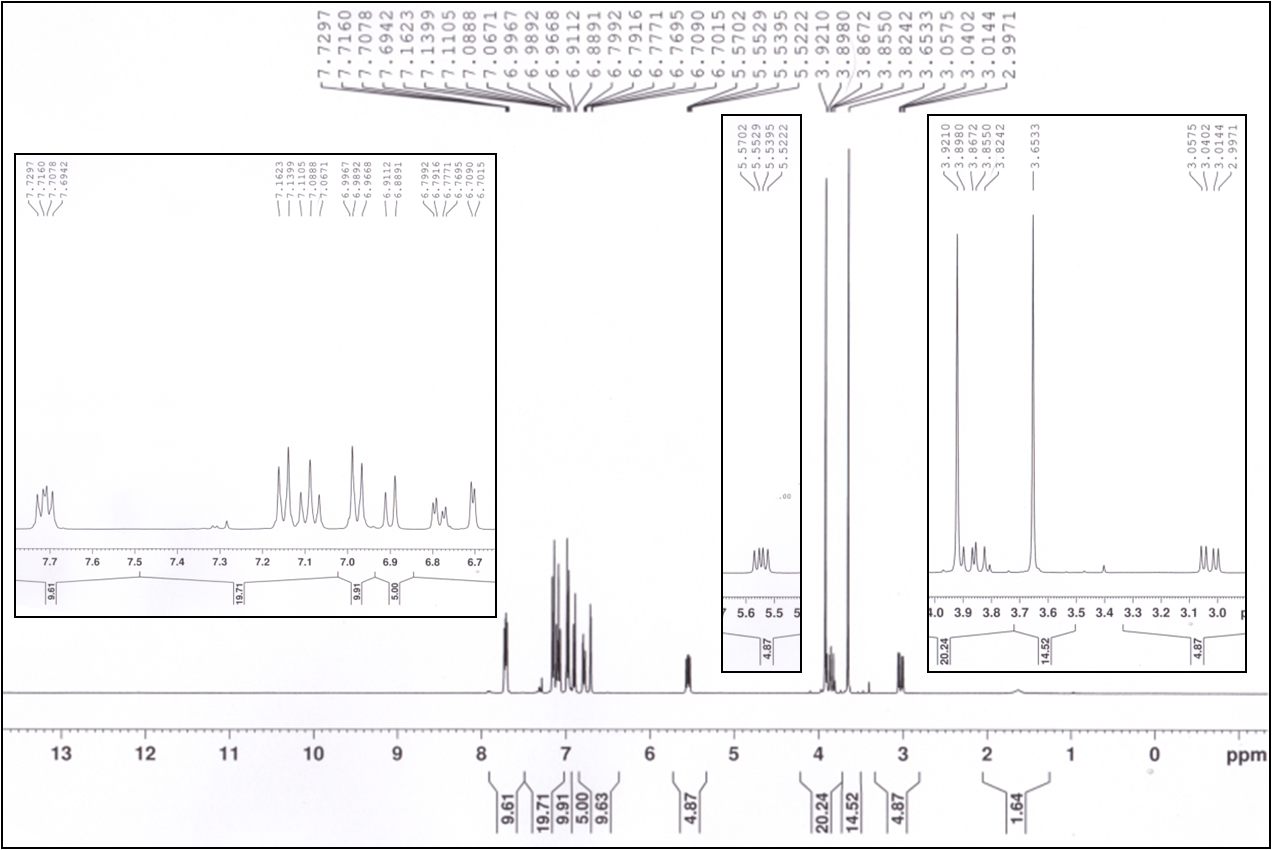


**Fig. S44.** ^1^H-NMR spectrum of compound **32** in CDCl_3_.


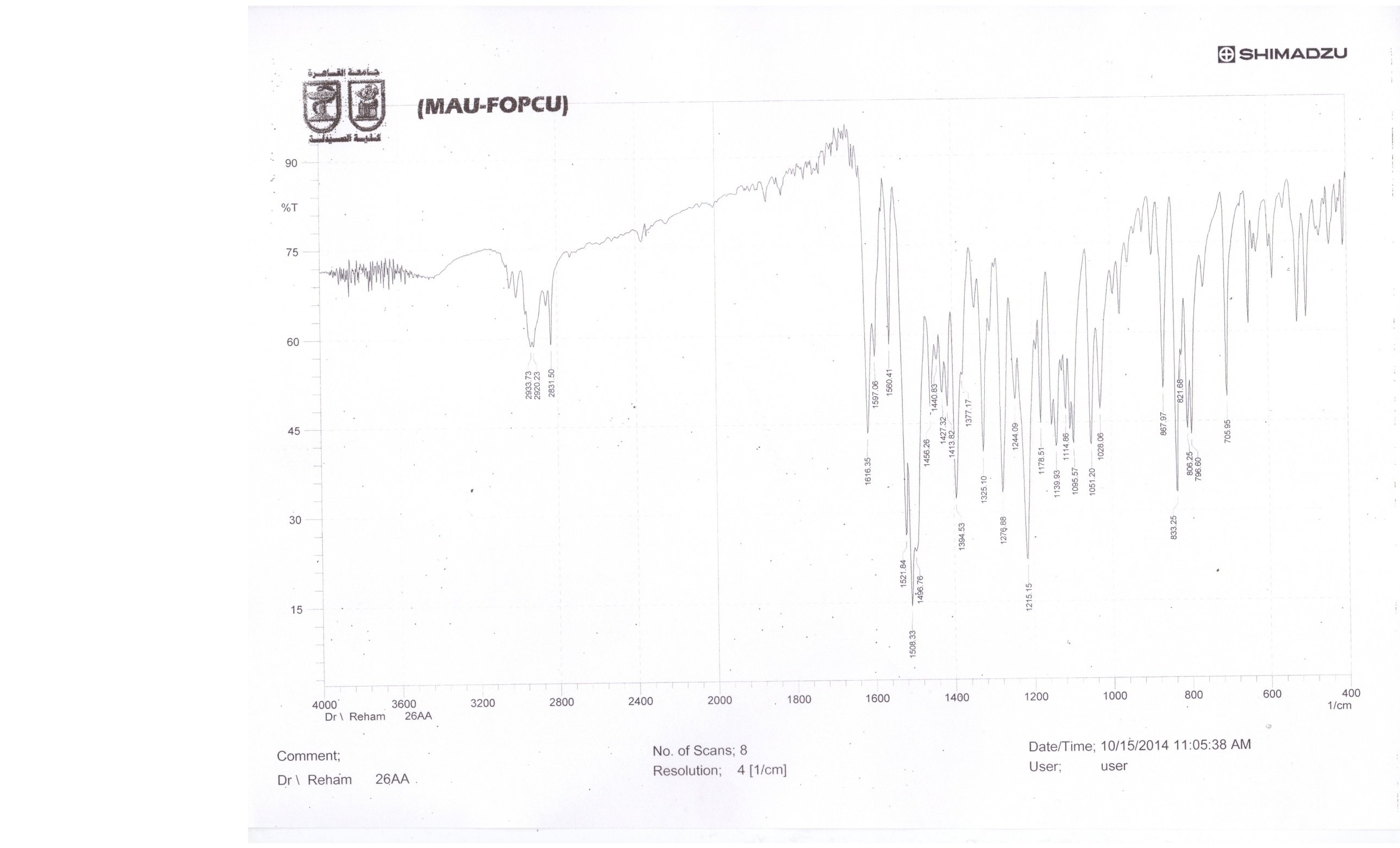


**Fig. S45.** IR spectrum of compound **33** (KBr pellet).

**
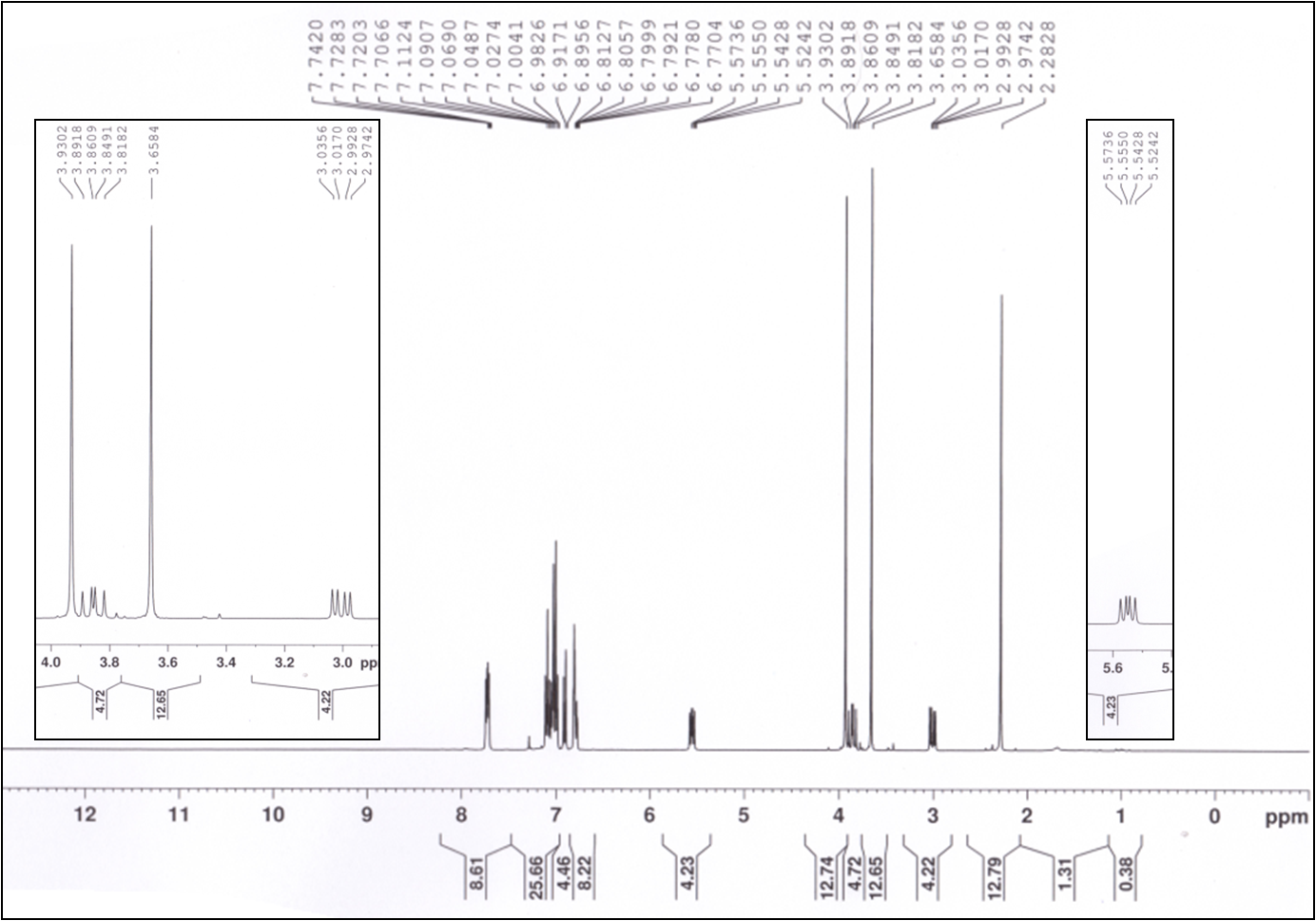
**

**Fig. S46.** ^1^H-NMR spectrum of compound **33** in CDCl_3_.


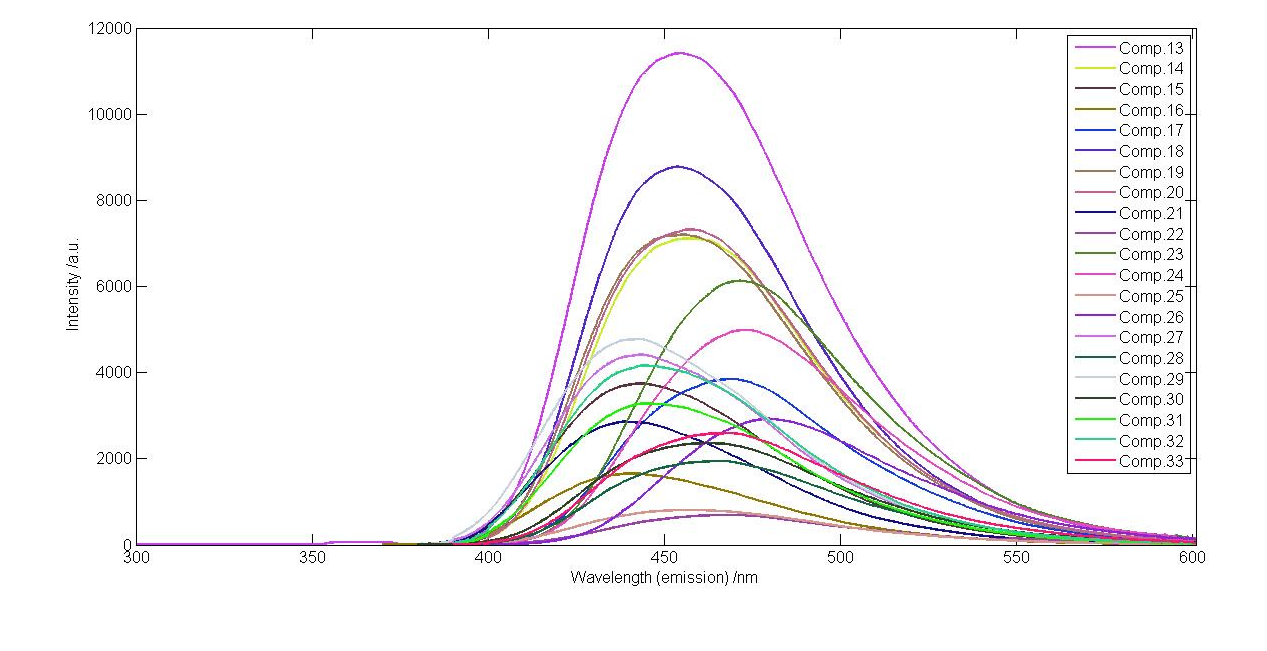


**S47.** Emission spectra of fluorescence compounds **13-33..**


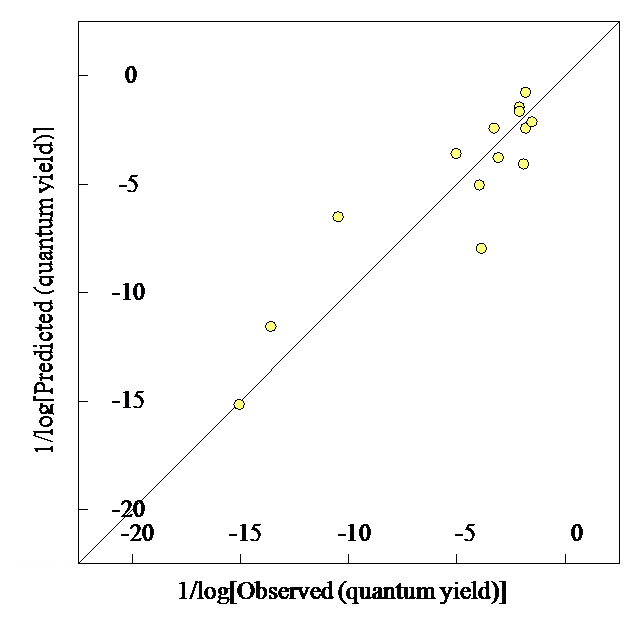


**Fig. S48.** BMLR-QSPR model plot of correlations representing the observed *versus* predicted 1/log(quantum yield, (Ф_s_) values for the subset group (A+B).


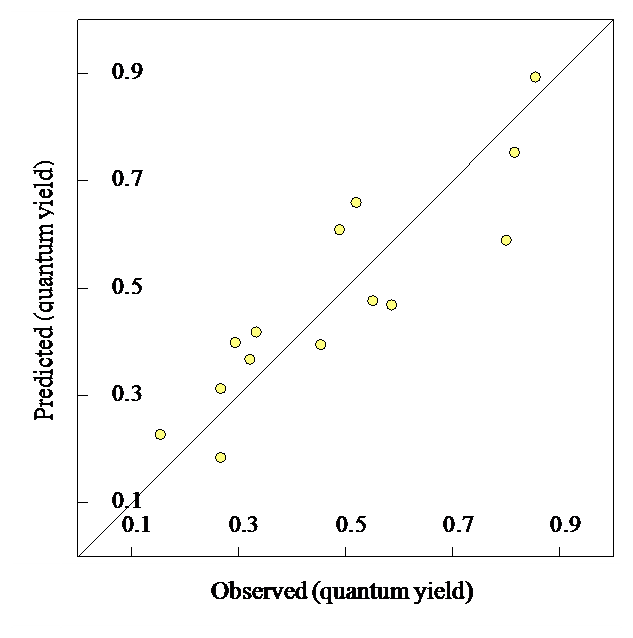


**Fig. S49.** BMLR-QSPR model plot of correlations representing the observed *versus* predicted quantum yield (Ф_s_) values for the subset group (A+C).


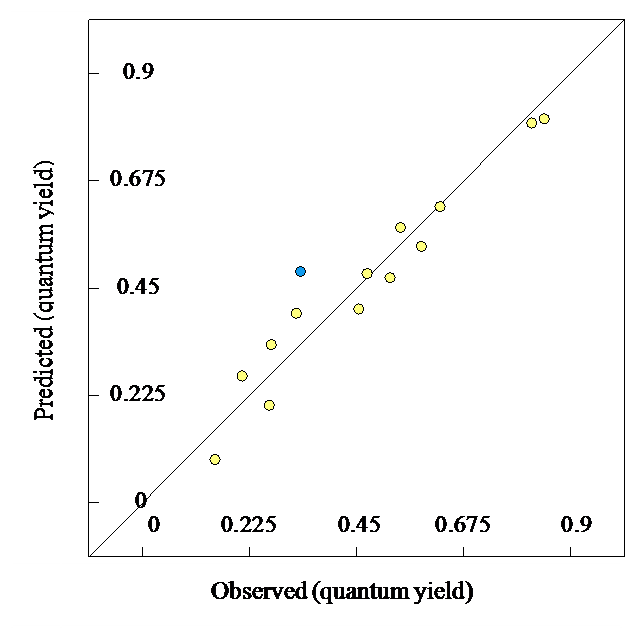


**Fig. S50.** BMLR-QSPR model plot of correlations representing the observed *versus* predicted quantum yield (Ф_s_) values for the subset group (B+C), compound **26** is an outliar.
